# Single-step fabrication of liquid gallium nanoparticles via capillary interaction for dynamic structural colours

**DOI:** 10.1038/s41565-024-01625-1

**Published:** 2024-02-22

**Authors:** Renu Raman Sahu, Alwar Samy Ramasamy, Santosh Bhonsle, Mark Vailshery, Archana S, Hemant Kumar, Tapajyoti Das Gupta

**Affiliations:** 1 Laboratory of Advanced Nanostructures for Photonics and Electronics, Department of Instrumentation and Applied Physics, Indian Institute of Science, Bengaluru, India; 2 Advanced Facility for Microscopy and Microanalysis, Department of Materials Engineering, Indian Institute of Science, Bengaluru, India

**Keywords:** Nanoparticles, Nanophotonics and plasmonics, Synthesis and processing, Sensors

## Abstract

Incorporating structural coloured materials in flexible and stretchable elastomeric substrates requires numerous steps that compromise their scalability and economic viability for prospective applications in visual sensors and displays. Here we describe a one-step approach for fabricating plasmonic Ga nanostructures embedded in a polydimethylsiloxane substrate exhibiting tunable chromaticity, in response to mechanical stimuli. The process exploits the capillary interactions between uncrosslinked oligomeric chains of the substrate and Ga metal deposited by thermal evaporation, as elucidated by a theoretical model that we developed. By tuning the oligomer content in polydimethylsiloxane, we attain a range of colours covering a substantial gamut in CIE (Commission Internationale de l’Éclairage) coordinates. This mechanochromic flexible substrate shows reversible response to external mechanical stimuli for ~80,000 cycles. We showcase the capabilities of our processing technique by presenting prototypes of reflective displays and sensors for monitoring body parts, smart bandages and the capacity of the nanostructured film to map force in real time.

## Abstract (web summary)

Exploiting capillary interactions of oligomers in polydimethylsiloxane and Ga allows single-step formation of closely spaced Ga nanodroplets in which gap plasmon resonances lead to mechanoresponsive structural colours, bypassing multiple chemical or lithographic steps.

## Main

Structurally coloured materials with mechanochromic responsiveness^1,2^ to external stimuli are the central component for visual communication^3^ and sensing^4^ applications. However, the numerous manufacturing steps necessary to fabricate them, make it difficult to scale up performances demonstrated at lab-scale level, hampering their widespread use in industrial and household settings^5^. Despite the several existing methods to fabricate structural coloured materials (spin coating^6,7^, 3D printing^8–10^, lithography and rubbing^11^), most of them require a rigid substrate, which remain inert (they are not mechano-chromic)^12,13^; additionally, top-down approaches are not viable for elastomeric substrates^14^. Lately, there have been some examples of chromogenic structures fabricated with mechanoresponsive properties^15^, where mechanical responsiveness is incorporated to elastomeric substrates by pattern transferring^16^ or by laser printing^17^; unfortunately, these steps tend to make the process even less scalable. Other proposed methods include using a photosensitive polymer with a periodic refractive index variation^18^, nanoimprinting^19^, or a combination of lithographic steps^20,21^. However, they still require lithographically fabricated masters and are not readily amenable to large-area fabrication. On the other hand, bottom-up, self-assembly fabrication approaches^22–24^ involve numerous processing steps, making them still largely unsuitable for a cost-effective fabrication with high fidelity^22,23^. These limitations necessitate a scalable, single-step fabrication technique that allows control of the nanoscale morphology with dynamically tunable structural colour.

A particularly popular substrate to incorporate or embed active materials for soft, flexible and deformable devices is polydimethylsiloxane (PDMS), due to its bio-compatibility and optical transparency in the visible region^25^. However, incorporating structural colours components in PDMS resulted in multiple chemical procedures^26–28^.

Recent advances have shown that Ga, a liquid metal with plasmonic properties on par with gold and silver^29,30^, and its alloys^31,32^ allow for the fabrication of flexible, bendable, and mechanically reconfigurable devices^33,34^, including electronic devices^35^. Thus far, however, scaling down Ga structures to the nanoscale has remained challenging, owing to its large surface tension (708 mN m^−1^) at room temperature^36^, which prevents precise fabrication of sub-100 nm feature dimensions required for application in the visible domain. The few attempts to fabricate sub-100 nm size Ga nanoparticles still require chemical methods, and the resultant nanoparticles are dispersed in a liquid medium^37–39^.

In this Article, we present a single-step fabrication of multiple chromogenic structures, achieved through the thermal evaporation of Ga onto PDMS substrates. We take advantage of the fact that thermally cured PDMS contains uncrosslinked oligomer chains that remain in a liquid-like state within the polymer matrix^40^. By exploiting the large difference in the surface tensions of Ga and liquid oligomers, we attain Ga nanodroplets with the polymer substrate filling the gap between each droplet. By controlling the external strain on the PDMS, we can manipulate their relative positions, which in turn changes the gap plasmon resonances. The plasmonic response of the Ga nanodroplets produce mechanically tunable structural colours that cover a substantial portion of chromaticity coordinates with excellent reproducibility over an 80,000 strain cycles. Further, we introduce a mathematical model that allows for the determination of the Ga nanostructure through its fluidic interaction with the liquid oligomers of PDMS.

## Fabrication of Ga-based nanostructures for colouration

**Fig. 1:**
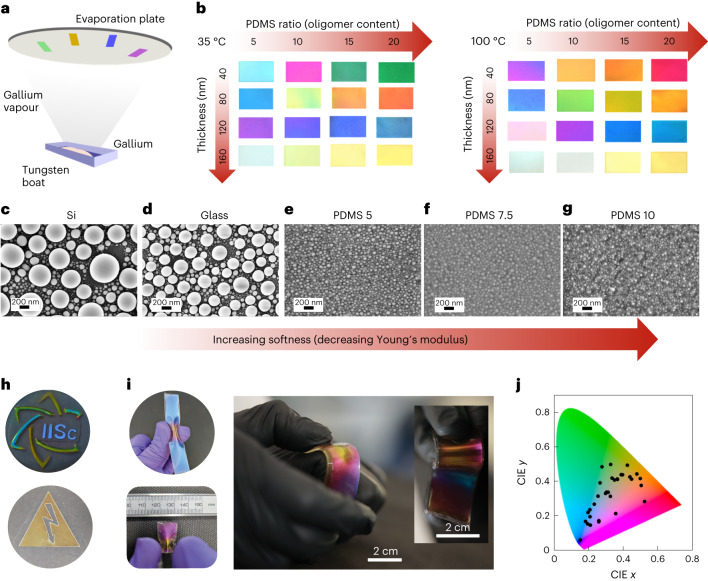
Fabrication and top-view morphology of Ga-deposited samples. **a**, Thermal evaporation of gallium. **b**, Colour palette (cropped images of Ga-deposited PDMS) obtained by adjusting oligomer content under different deposition parameters. **c**–**g**, Top-view SEM images of Ga deposited onto Si (**c**), glass (**d**), PDMS 5 (**e**), PDMS 7.5 (**f**) and PDMS 10 (**g**). Scale bar, 200 nm. **h**, Chromogenic structures on large areas (30 cm^2^) for IISc logo and symbols. **i**, Mechanoresponsiveness of structural colours. **j**, Chromaticity coordinates of fabricated samples on CIE diagram. Source data

The fabrication approach we employ is depicted in Fig. 1a and detailed in Methods and Supplementary Information (Supplementary Fig. 1a–c). PDMS ratio *X* is made by proportionate mixing of *X* g of base and 1 g of curing agent (Sylgard 184, Dow Corning) during the substrate preparation. The proportion of the curing agent in PDMS governs crosslinking and substrate softness (Supplementary Fig. 1b). It also influences the volume of oligomers in the crosslinked PDMS matrix, crucial for the final optical properties^41^.

As we vary the PDMS ratio (from PDMS 5 to 20), substrate temperature, and deposited Ga thickness, a distinct colouration is observed (Fig. 1b). Additionally, adjusting substrate softness (Si), glass and different PDMS proportions (Fig. 1c–g) reveals varied Ga nanoparticle size distributions on the substrates (Fig. 1c–g and Supplementary Fig. 1d,e). On typical rigid substrates such as Si (Young’s modulus approximately 150 GPa)^38^ and glass (Young’s modulus 60 GPa)^42^, the top-view scanning electron microscopy (SEM) images (Fig. 1c,d) show large size distribution of Ga droplets around 70 nm and 40 nm, respectively, which are poly-dispersed. While in PDMS substrates (Fig. 1e–g and Supplementary Fig. 1d), droplets are narrowly dispersed (radii with a mean and standard deviation of around 20 nm and 10 nm, respectively) as depicted in the histogram plot (Supplementary Fig. 1e). The average sizes of the Ga nanodroplets, as well as their dispersity, decrease as we increase the substrate softness (Fig. 1c–g and Supplementary Fig. 2), indicating a positive correlation between the nanostructure’s sizes and the elastic modulus of the substrate.

The elastic modulus of PDMS substrates ranges from 0.3 to 1.3 MPa (Supplementary Fig. 2), considerably less than rigid substrates. Despite this, colour variations prompt further exploration of factors influencing Ga nanostructure formation beyond substrate softness. Furthermore, the wide variation of colour despite the slight change in size distribution observed from top-view SEM images (Fig. 1c–g), as shown in Fig. 1b, necessitates a detailed study of cross-sectional morphology of the Ga nanostructure. At the foresight, one can infer from the processing of the PDMS substrate that the oligomers remaining in the PDMS substrate participate in the nanostructure formation more prominently than the elastic modulus of the substrate does, as will be explained in the following sections. Having identified that the processing step of mixing the PDMS base with the curing agent determines the softness and oligomer content in the substrate, the fabrication approach is simple and scalable. As evident from Fig. 1h, large areas order of a few centimetres squared can be fabricated, such as the IISc logo structurally coloured over 30 cm^2^, an area similar to those of Petri dishes. The PDMS substrate, being flexible, allows for dynamic tuning of the structural colour via mechanical deformation (Fig. 1i). The wide array of structural colours, thus fabricated, spans a large fraction of chromaticity coordinates (Fig. 1j) by leveraging on two key factors: (1) substrate softness and, more prominently, (2) oligomer content in PDMS.

## Role of substrate properties: softness and oligomer content

**Fig. 2:**
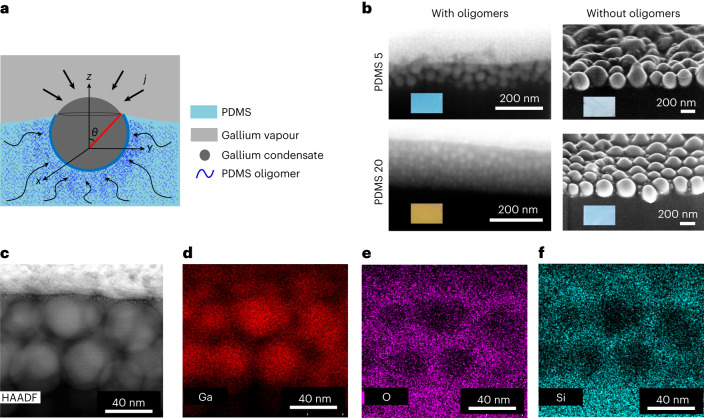
Effect of oligomers from cross-section of Ga-deposited PDMS. **a**, Schematic of oligomer extraction by Ga nanodroplet on PDMS. **b**, Cross-section SEM images of PDMS 5 and 20, with and without oligomers. Inset: image of the fabricated sample. **c**, HAADF image of the cross-section of Ga deposited on PDMS 5. **d**–**f**, EDAX map of Ga (**d**), Si (**e**) and O (**f**).

The interaction between capillary and elastic forces in the nanoscale is more prominent in soft substrates than hard substrates^43^. The micron- or nano-sized droplets on soft substrates form a dimple on the substrate due to Laplace pressure inside the droplet and the surface tension force at the three-phase contact line of the droplet pulls the substrate^44–46^ (Fig. 2a). The cross-sectional SEM image of PDMS 5 and 20 reveals the multi-layers of Ga nanodroplets beneath the top surface rather than forming a single layer with deformation on the substrate (Fig. 2b, left). We observe that increasing the PDMS ratio leads to more layers and smaller Ga particle size, mainly due to volume conservation. Additionally, Ga nanodroplet radius decreases with depth into the PDMS substrate (Fig. 2b, left).

Although the oligomers do not have an important role in determining the elastic modulus of the substrate (Supplementary Fig. 2), their presence determines the Ga nanostructures that form upon deposition on the substrate. On removal of the oligomers from PDMS by toluene treatment (Supplementary Information section 1.2) before Ga deposition, we observe a monolayer of Ga nanodroplets with dimple formation on the substrate (Fig. 2b, right, and Supplementary Figs. 3 and 4). In addition, removing the oligomers eliminates the difference in the size distribution of Ga nanodroplets on different PDMS ratios (Fig. 2b, right, and Supplementary Fig. 4). The resulting colours resemble bluish-white (Fig. 2binset and Supplementary Fig. 5) as observed in glass and Si substrates. Thus, the importance of oligomers in PDMS to form distinct structural colours, as shown in Fig. 1a, is substantiated.

## Multi-layer formation of Ga nanodroplets in PDMS

The embedding of Ga nanodroplets (Supplementary Information section 1.5) in the substrate suggests the existence of a method for the oligomers to encapsulate them. Toluene treatment of the samples to remove oligomers after the fabrication results in removal of structural colour (Supplementary Fig. 6a,b) and regaining of transparency of PDMS. This can be explained if fluidic nature of oligomers is assumed. To quantitatively understand Ga nanodroplets on PDMS, we assume oligomers to be in in the liquid phase and spread on the nanodroplets to minimize the surface energy of the system.

Ga is in a liquid state during the fabrication (Supplementary Information section 1.6 and Supplementary Figs. 7 and 8). The spreading of oligomers occurs over Ga to minimize the surface energy, which occurs when the spreading parameter is positive. The spreading parameter is defined as

1 $$S={\gamma }_{{{\mathrm{Ga}}}}-\left({\gamma }_{{{\mathrm{oligomers}}}-{{\mathrm{Ga}}}}+{\gamma }_{{{\mathrm{oligomers}}}}\right)$$

where ${\gamma }_{{{\mathrm{Ga}}}}$ and ${\gamma }_{{{\mathrm{oligomers}}}}$ are the surface tension of the Ga and oligomers, respectively, and ${\gamma }_{{{\mathrm{oligomers}}}-{{\mathrm{Ga}}}}$ is the interfacial tension between the Ga and oligomers. For the configuration of the Ga droplet on PDMS, interfacial tensions ${\gamma }_{{{\mathrm{Ga}}}}$, ${\gamma }_{{{\mathrm{oligomers}}}}$ and ${\gamma }_{{{\mathrm{oligomers}}}-{{\mathrm{Ga}}}}$ are 650, 20 and 590 mN m^−1^, respectively (Methods). With a spreading coefficient of 40 mN m^−1^, PDMS oligomers can create a cloaking layer around Ga droplets, confirmed by high-angle annular dark field (HAADF) image (Fig. 2c). The energy-dispersive X-ray (EDAX) map of Ga (Fig. 2d) reveals droplet spatial distribution, while Si (Fig. 2e) and O (Fig. 2f) EDAX maps indicate the presence of PDMS oligomers between droplets. Oligomers resist droplet coalescence, allowing successive layer formation as observed in HAADF image and EDAX maps. Slower encapsulation of subsequent layers results in larger droplet growth time, leading to decreasing radii with layer depth, aligning with experimental observation (Fig. 2b, left).

Temperature considerably influences Ga nanostructures (Supplementary Figs. 9–12), with the samples remaining robust against wide temperature variations (Supplementary Figs. 13 and 14). Substrate temperature during Ga evaporation (Supplementary Information section 1.7) determines the equilibrium between Ga vapour and liquid states. Higher substrate temperatures favour the vapour phase, inhibiting condensation and nanodroplet growth, resulting in a smaller size distribution (Supplementary Fig. 12). Consequently, substrate temperature is maintained constant during deposition. In subsequent discussions, we assume a constant temperature throughout.

## Modelling: Ga nanodroplet layer formation on PDMS substrates

To understand nanostructure formation interactions, we developed a mathematical model replicating experimentally observed trends:

- Ga nanodroplet sizes vary with PDMS ratios: higher PDMS ratio yields smaller nanodroplet radii for the same deposition parameters.
- Ga nanodroplet radii decrease with depth.
- Higher PDMS ratios lead to an increased number of layers of Ga nanodroplets.

**Fig. 3:**
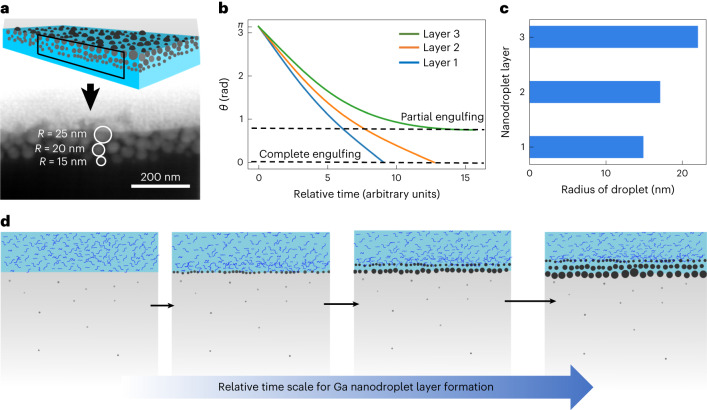
Modelling of Ga nanodroplet layer formation. **a**, Schematic cross-section view of Ga deposited on PDMS and the cross-section SEM image from a thin slice cut with FIB. **b**, Relative time scale of engulfing of the Ga nanodroplet layers. **c**, Radii of Ga nanodroplets as predicted from the SGE equations. **d**, Schematic depiction of the formation of layered nanodroplets when Ga is thermally evaporated onto PDMS. The length of the black arrows depicts the relative time it takes to form one layer to the next. Source data

Figure 3a illustrates the structure schematic and cross-section SEM image, depicting radii trends with depth. Thermal evaporation produces Ga vapours that condense, forming spherical condensates on the substrate to minimize surface energies (Supplementary Information section 2.1 and Supplementary Figs. 15 and 16). Upon reaching the substrate, two phenomena occur: (1) substrate deformation due to Laplace pressure (Supplementary Information section 2.2), and (2) liquid oligomers extracting to engulf condensate droplets (Supplementary Information sections 2.3 and 2.4). Engulfing depends on oligomer volume, quantified by the rate of change of the engulfing angle (Supplementary Figs. 17 and 18). Nanodroplet growth, defined by the increase in radius over time from condensed Ga atoms (Supplementary Fig. 19), determines sizes and layer number along PDMS depth (Supplementary Figs. 20–23). Interplay of events is detailed in Supplementary Information section 2.5, describing oligomer migration (Supplementary Information section 2.6), Ga nanodroplet growth (Supplementary Information section 2.7) and engulfing (Supplementary Information section 2.8) through the following three equations:

2 $$\frac{{\rm{d}}f}{{\rm{d}}t}=-{k}_{{\mathrm{s}}}\left(\;f-2\pi {R}^{2}w\left(1+\cos \theta \right)\right)\left(2\pi R\sin \theta \right)$$

3 $$\frac{{\rm{d}}R}{{\rm{d}}t}={k}_{{\mathrm{\rho}}}\left(1-\cos \theta \right)$$

4 $$\frac{{\rm{d}}\theta }{{\rm{d}}t}=-{k}_{{\mathrm{e}}}\left(\;f-2\pi {R}^{2}w\left(1+\cos \theta \right)\right)$$

where, $f$ is the volume of liquid oligomers present in a given substrate volume, in units of nm^3^, $R$ is the radius of the Ga droplet, and $\theta$ is the engulfing angle. The flux of Ga atoms condensing onto the droplets is given by $J$, and $w$ is a thin layer of oligomers encapsulating the nanodroplet during engulfing. The constants ${k}_{{\mathrm{s}}}$, ${k}_{{\mathrm{\rho}}}$ and ${k}_{{\mathrm{e}}}$ are constants of proportionality for equations (2), (3) and (4), respectively (Supplementary Information sections 2.5–2.8 for details).

These equations describe three simultaneous events for a single Ga nanodroplet: the presence of oligomers at the Ga–PDMS interface, the growth of the Ga nanodroplet during engulfing, and the engulfing of the nanodroplet by liquid oligomers (Fig. 2a). Equations (2), (3) and (4) are termed the substrate (S), growth (G), and engulfing (E) equations, respectively, and are solved iteratively for each layer (Supplementary Information section 2.9). Figure 3b illustrates the relative time scale of immersion for the first and second layers of Ga nanodroplets, supporting the explanation that the first layer forms the fastest, with subsequent layers taking longer. The third layer is partially immersed due to insufficient oligomers, halting further engulfing (Fig. 3b). Final droplet sizes in each layer are determined by the available growth time before engulfment. The parameters *w* and SGE constants ${k}_{{\mathrm{s}}}$, ${k}_{{\mathrm{\rho}}}$, and ${k}_{{\mathrm{e}}}$ are determined by fitting for a given PDMS substrate and deposition parameters (Supplementary Fig. 24). ${k}_{{\mathrm{\rho}}}$ depends on deposition parameters, while ${k}_{{\mathrm{e}}}$ and ${k}_{{\mathrm{s}}}$ depend on substrate–liquid metal interaction. With a specific set of parameters, one can obtain constants ${k}_{{\mathrm{s}}}$, ${k}_{{\mathrm{e}}}$ and ${k}_{{\mathrm{\rho}}}$ (Supplementary Fig. 24). These equations predict the number of layers and size trend of Ga droplets on PDMS with oligomer content, rate, and deposition thickness. These equations offer practical utility in designing process steps for obtaining desired Ga nanodroplet size and layers (see Supplementary Figs. 25 and 26, showing the use of SGE equations to determine structural dependence of nanodroplets on deposition rate).

Based on the assumption of the fluidic nature of oligomers, the model explains the above three experimental trends together, supporting the picture of fluidic oligomers infused in the crosslinked PDMS matrix. Thus, it provides a framework for the layered nanodroplet structure formation mechanism via fluidic interactions.

## Spectral trend caused by oligomer content in the substrate

**Fig. 4:**
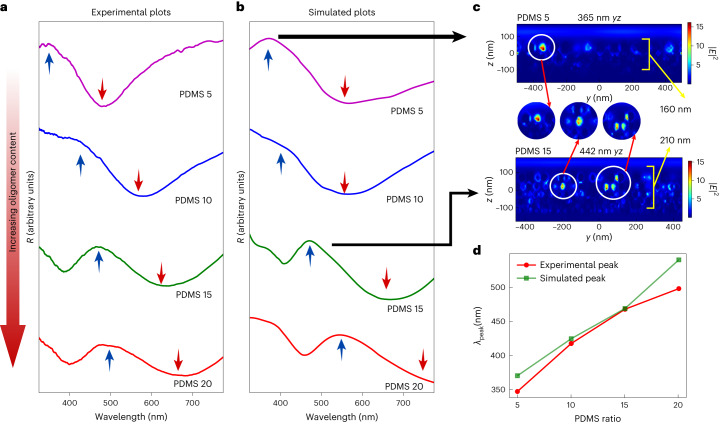
Spectral trend with oligomer content. **a**, Reflectivity spectrum obtained experimentally. **b**, Reflectivity spectrum from FDTD simulations. **c**, The electric field intensity (${\left|{E}\right|}^{2}$ with units V^2^ m^−2^) of simulated PDMS 5 structures at 365 nm and PDMS 15 at 442 nm. **d**, Wavelength at which the reflectivity peaks versus PDMS ratios. Source data

Ga nanodroplet morphology on PDMS, influenced by oligomer content, impacts the spectral properties. Experimentally, as PDMS ratio increases, reflectivity spectra red shifts (Fig. 4a). The optical properties of Ga are determined by its wavelength-dependent complex refractive index (Supplementary Fig. 28). The spectra obtained from the simulation match the experimental trend (Fig. 4b, Supplementary Information section 3.1 and Supplementary Fig. 29).

A minimum wavelength exists for a given structure with a specific number of layers, exciting fields between inter-layer and intra-layer Ga nanodroplets (Supplementary Fig. 30). The highest reflectivity spectrum peak corresponds to the wavelength interacting with all layers (Fig. 4c). Despite the anticipated blue shift from smaller Ga nanodroplets with increased oligomer content, we observed a red shift (Fig. 4d). This occurs as the layer thickness containing Ga nanodroplets increases with adjusted oligomer content (refer to Supplementary Figs. 31 and 32). Thus, the collective effect from all layers determines the spectral features of the sample.

## Dynamic tuning of the optical properties

**Fig. 5:**
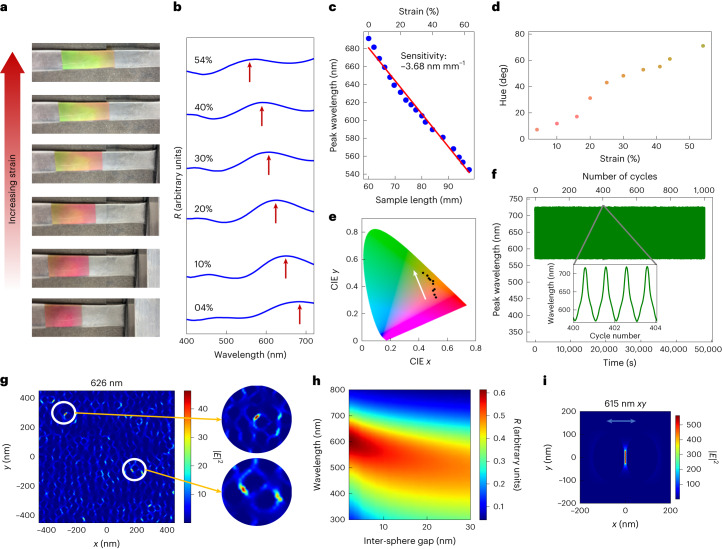
Uniaxial strain on Ga-deposited PDMS. **a**, Colour of the sample from pink to green. **b**, The blue shift of the reflectivity spectrum on applying linear uniaxial strain. **c**, The peak wavelength of reflectivity versus length of the sample and strain (%). **d**, Change of hue with respect to applied strain. **e**, Change of chromaticity coordinates with strain. **f**, Peak reflectivity versus time of a periodic uniaxial linear strain for 1,000 cycles. **g**, Electric field intensity (${\left|{E}\right|}^{2}$ with units V^2^ m^−2^) plot across one of the layers of simulated structure. The circularly zoomed regions depict the occurrence of gap plasmon resonances. **h**, Simulation of reflectivity from a dimer structure of two spheres of radius 50 nm placed near each other with a varying gap. **i**, Enhanced field intensity (${\left|{E}\right|}^{2}$ with units V^2^ m^−2^) between two Ga nanospheres with a 2 nm surface-to-surface gap in between them. Source data

Stretching induces change in interparticle gaps along the stretching direction. We examined Ga nanoparticle interaction in a single layer to understand optical properties during stretching. Local hotspots in electric field intensity plots (circularly zoomed regions in Fig. 4c) represent plasmonic resonances from gaps between closely positioned Ga nanodroplets. Exciting the entire structure involves a contribution from all intra and inter-layer gap plasmons, crucial for determining spectra in the visible region. Applying uniaxial strain experimentally induces a colour change (Fig. 5a) due to a blue shift in reflectivity spectra (Fig. 5b and Supplementary Videos 1 and 2). Spectral features shift by 3.68 nm per millimetre of sample length change (Fig. 5c). The hue changes from 6° to 65° (Fig. 5d), reflecting a discernible shift in colour on uniaxial stretching, evident in chromaticity coordinates (Fig. 5e). The repeatable and reversible spectral change over 1,000 cycles of applied periodic strain (Fig. 5f) of at least 60% (Supplementary Fig. 33, Supplementary Information section 3.2 and Supplementary Video 3) establishes it as a reliable, long-lasting chromatic sensor.

Gap plasmon resonances, arises from plasmonic excitation in dimeric pairs (Fig. 5g). Uniaxial strain widens the gap between closely placed droplets, resulting in a blue shift of reflectivity spectra (Fig. 5h) when polarization vector is along the gap (Fig. 5i, Supplementary Information sections 3.3 and 3.4, and Supplementary Fig. 34). Random distribution of Ga nanodroplets in the plane of the layer allows unpolarized light to excite the gap plasmons. Whenever the interparticle gap increases due to mechanical deformation, reflectivity spectra blue shifts leading to colour change.

On the other hand, the size of the Ga nanodroplets decides the scattering contribution to the spectra. No spectral shift in reflectivity is observed on changing interparticle gap from the dimer structure when polarization is perpendicular gap axis (Supplementary Figs. 35 and 36). Hence, the scattering component of spectra is identified from the spectral features unresponsive to mechanical deformation. As expected from the Mie theory, the reflectivity peaks occurring due to scattering occurs in the ultraviolet (UV) region (Supplementary Fig. 37). Thus the UV spectral features obtained experimentally (Supplementary Fig. 38). and corroborated by simulations (Supplementary Fig. 39) are attributed to scattering by the Ga nanodroplets.

After the samples are taken out of the evaporation chamber, atmospheric oxygen diffuses into the PDMS substrate, forming a 2–3-nm-thin native Ga oxide layer around nanodroplets. Despite its presence, chromaticity changes are visually undetectable. Stretching simulations show optical trends like those without an oxide layer. The oxide layer enhances structural stability against deformations without affecting optical performance (Supplementary Figs. 40–45). Encapsulation with an additional PDMS layer further enhances structural stability and protects against environmental deterioration, exhibiting endurance against deformations, mechanical strain, ageing, washability, high temperatures, static stretch and physical contact deformations, making them versatile for various applications (Supplementary Figs. 46–50).

## Applications

As shown above, any mechanical deformation resulting in the change of inter-droplet gap will result in the corresponding colour change, thus making the sample responsive to local and global strain variation. The minimum strain detected via chromaticity change is subject to the lowest stress required to effect a change in the interparticle gap. Mechanoresponsiveness of colour enables the qualitative gauging of strain and stress and quantitative measurements when images are coupled to the hue channel of colour space or the chromaticity coordinates (Supplementary Information section 4.1 and Supplementary Figs. 51 and 52). Our experiments show our sensors detect local and global strains (Supplementary Video 4), monitor healthcare (Supplementary Video 5) and detect body motion (Supplementary Video 6). Results suggest potential applications in prosthetics and soft robotic systems.

**Fig. 6:**
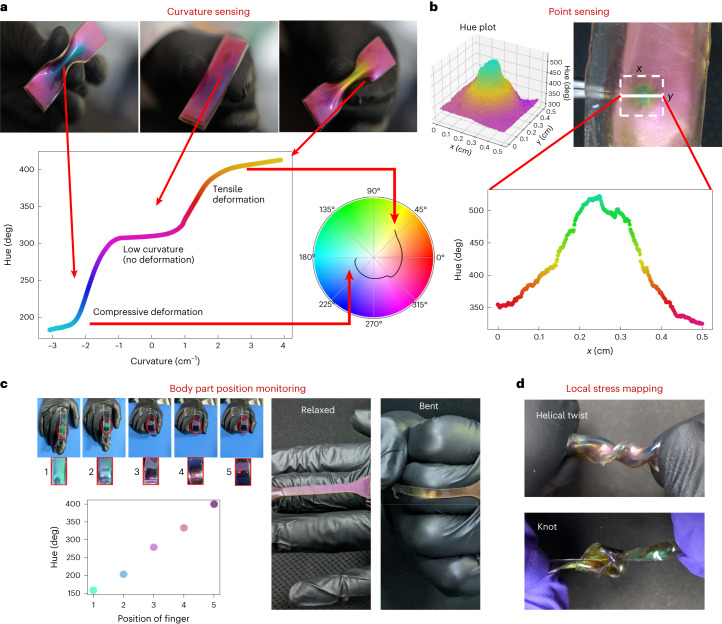
Mechanoresponsive applications of Ga-deposited PDMS. **a**, Curvature sensing, hue (deg) versus curvature of the sample. Inset: hue circle depicting the line along which the curvature varies. **b**, Colour change due to point stress applied by a tweezer tip. Top: the 2D surface plot illustrates hue’s spatial variation in the sample region marked by the dashed white boundary. Bottom: hue versus distance plot along the line drawn by solid white on the sample. **c**, Chromatic responsiveness applied in detecting the bending of fingers. **d**, Local variation in colour of the sample due to a helical twist (top), and a knot (bottom), indicating local strain map. Source data

Figure 6a shows curvature mechanoresponsiveness. Bending laterally shifts colour from pink to yellow (tensile, positive curvature) and pink to blue (compressive, negative curvature). Local surface curvature changes where Ga is deposited leads to colour variation (Supplementary Fig. 53), illustrated in the hue plot (Fig. 6a, bottom). The hue circle in Fig. 6a highlights large hue variation through dynamic curvature tuning of the sample.

Figure 6b exhibits the sensitivity of the sample to the point stress (Supplementary Video 7). Here the sample is subjected to point force by a tweezer, thus resulting in a visibly distinct colour change from pink to green. The visibly large change in the hue, as depicted in the surface plot, holds the potential to obtain the local stress varying in a region within 25 mm^2^ or lower, determined by the source of the force, as shown in the plots (Fig. 6b).

The wide structural colour range suits static reflective displays, while mechanoresponsive property is valuable for sensing applications. In soft substrates, mechanoresponsiveness is ideal for wearable interfaces like a body movement sensor (Fig. 6c and Supplementary Video 6). The sample’s curvature change yields different colours, useful for manoeuvring systems with varied bending angles of the finger. Additionally, flexible device colour analysis detects local strain variations, enabling real-time stress mapping on mechanically strained samples, such as a helical twist (Supplementary Video 8) or a knot (Supplementary Video 9) (Fig. 6d).

## Conclusions

By exploiting the capillary interaction between liquid Ga and the oligomers interspersed in the PDMS crosslinked matrix, we have presented a scalable fabrication technique to realize multiple chromogenic and mechanochromic structures via a single-step physical vapour deposition process. Our technique demonstrates that liquid metals, like Ga and its alloys, can find its way in applications in visible photonics, thanks to the control in the formation of the nanoparticle on an elastomeric substrate (Supplementary Tables 3 and 4). Additionally, the wide span of chromaticity coordinates, the stability of our samples against ageing, temperatures, washability in soap and solvents, physical deformations and strain cycles can be useful for potentially numerous applications in reflective displays, aesthetic crafts, decorations and smart windows as well as, wearable, flexible and stretchable photonic devices. Our processing technique demonstrates substrate selectivity as a distinctive feature, providing versatility for specific requirements. Additionally, PDMS can be effectively employed in various processes, including roll-to-roll^47^ thermal evaporation. It must be noted that including additional process steps, such as harnessing template-assisted dewetting^19,48^, can lead to metasurfaces of Ga nanostructures that should improve the spectral purity of the structural colours^49^, and potentially enhance sensitivity. Consequently, our proposed technique can seamlessly integrate with subsequent processing steps to achieve enhanced functionalities and properties.

## Methods

### Fabrication of samples

#### Preparation of PDMS and deposition of Ga

PDMS soft substrates are prepared by mixing the liquid PDMS base and crosslinking agent in various ratios (Dow Corning, Sylgard 184). The following notation PDMS XX represents 1 part of the curing agent is added to *XX* parts of liquid PDMS base in weight ratio. Substrate softness increases with an increase in the proportion of liquid PDMS base. To fabricate structural colours, PDMS 5, PDMS 10, PDMS 15 and PDMS 20 are chosen as soft substrates. The mixture is stirred thoroughly and desiccated to remove the bubbles. The solution is poured onto a polystyrene Petri dish and cured at 80 °C in an oven for 2 h to attain a cured soft substrate, which inherently has some uncrosslinked liquid PDMS chains. Liquid gallium (Thermo Scientific Chemicals, 99.999% metal basis, packaged in polyethylene bottle) is then thermally evaporated (HHV thermal evaporator) and deposited onto these substrates to form nanodroplets. The thickness and temperature of the substrates are monitored via inbuilt thickness and temperature monitoring sensors, respectively.

#### PDMS superstrate encapsulation for protection of Ga-nanodroplet structures

The fabricated Ga-nanodroplet structure is protected against direct physical contact by another layer of PDMS as a superstrate. The pre-thermal cured degassed mixture of PDMS base and the curing agent is poured on the fabricated structural colours. The PDMS ratio of the superstrate is the same as the substrate. The samples are then thermally cured at 80 °C in oven for 2 h.

#### Toluene treatment of PDMS substrates

To examine the role of uncrosslinked liquid PDMS chains, cured PDMS substrates are stirred in a toluene bath for a specific duration of time. This process dissolves the uncrosslinked liquid PDMS chains since toluene acts as a suitable solvent. During this period, toluene is changed in an interval of 24 h. Subsequently, the PDMS substrate is immersed in ethanol for 12 h and kept in a vacuum oven at 70 °C for 12 h to remove the solvent in the sample.

### Simulation of gallium nanostructures on PDMS

Finite difference time domain algorithm in the commercially available software Lumerical is used for simulations. The size distribution of gallium nanodroplets is determined from the SEM images. The radii of the Ga nanodroplets in a given layer are sampled from the size distribution, while their location is sampled from the uniform distribution spanning the top (*xy* surface, +ve *x* normal) of the PDMS substrate. The PDMS substrate is a homogeneous material with a refractive index of 1.4. The optical constants for Ga are imported as the real and imaginary parts of the refractive index. The finite-difference time-domain (FDTD) region is chosen as 1 μm × 1 μm, with periodic boundary conditions along the *x* and *y* directions. The area chosen is large enough to encompass randomly distributed Ga droplets so that periodicity effects are negligible.

The total field scattered field source is used for the dimer simulation with an interparticle gap. The frequency-domain field and power monitor are put behind the injection plane of the total field scattered field source to obtain the back-scattered power for understanding the spectral trends. For the FDTD boundary condition perfectly matched layer is used on all sides. To obtain intensity plots, a frequency-domain field profile monitor is placed parallel to the injection plane, containing the centres of both spheres.

### Mechanical stretching of gallium nanostructures on PDMS

Gallium-deposited PDMS substrate is cut into a 5 cm × 1 cm rectangular shape. Both the sample ends are clamped onto stretching equipment operated through a Stepper motor controlled by Arduino. It is programmed for linear stretching as well as for applying a periodic strain.

### Measurement of Interfacial Tension in interfaces

The interfacial tensions are measured through the Pendant drop method with Goniometer, Dataphysics OCA 25.

### Microscopy analysis

#### SEM

The SEM images are taken with Zeiss-Gemini SEM, ULTRA 500 model in the inlens and secondary electron mode. The samples are sputtered with 10 nm gold to form a conducting layer for electronic charge dissipation.

#### Focused ion beam

Thermo Fisher Scientific Scios Dual Beam focused ion beam (FIB)–SEM system is used to make TEM lamella of Ga-deposited PDMS, which were placed on a copper grid for cross-section imaging in secondary electron mode.

#### Scanning transmission electron microscopy

ThermoFisher Titan Themis scanning transmission electron microscope is used to obtain HAADF scanning transmission electron micrographs using a field emission gun with an extractor voltage of 300 kV. The elemental composition of Ga embedded in PDMS was obtained by EDAX elemental mapping using a super X detector in scanning transmission electron microscopy nanoprobe mode in TITAN.

### Optical characterization

The Ocean Optics FLAME series UV–visible spectrophotometer is used to obtain the reflectivity at normal incidence for each sample. The package python-Seabreeze is used to access the spectrophotometer in a Python program. The reflectivity thus obtained is used for real-time computation of the sample’s CIE *x* and *y* coordinates and hue. The packages numpy and matplotlib are used for array manipulation and visualization (graphs and animations) respectively.

The reflectivity spectra obtained are smoothened by box averaging around a small window of the raw data points to remove device-sourced noises.

### Stability analysis

#### Washability

The samples with PDMS superstrate encapsulation were put in a soap solution and magnetically stirred for 12 h. The samples were dried on a Petri dish, allowing the solution to evaporate before the reflectivity spectra were acquired.

#### Stability against solvents (toluene and ethanol)

The samples with PDMS superstrate encapsulation were put in a toluene and magnetically stirred for 6 h, followed by 3 h of stirring in ethanol. The solvents infuse into the PDMS substrate and superstrate making them swell and enlarge in size. The samples were put on a Petri dish till the solvents evaporate and the samples regain their initial shape, following which reflectivity spectra were obtained.

#### Stability in high temperature

The samples are put on hot plate on top of black-powder-coated mild steel. Spectrum acquisition was started when the temperature of the hot plate reached 180 °C. The extremum values of the spectra were obtained in real time, while the temperature was maintained at 180 °C for 12 h.

#### Stability of stretched samples

The sample was pre-stretched before the spectrum acquisition. While maintaining the strain, the reflectivity spectra were obtained in real time.

## Online content

Any methods, additional references, Nature Portfolio reporting summaries, source data, extended data, supplementary information, acknowledgements, peer review information; details of author contributions and competing interests; and statements of data and code availability are available at 10.1038/s41565-024-01625-1.

### Supplementary information

Supplementary information Supplementary Figs. 1–53, discussion and Tables 1–4.

Supplementary Video 1 Mechanoresponsivity on strain.

Supplementary Video 2 Response to uniaxial strain in different samples.

Supplementary Video 3 Real time acquisition of spectral data and chromaticity coordinates.

Supplementary Video 4 Response to bending.

Supplementary Video 5 Structurally coloured smart bandages.

Supplementary Video 6 Finger position monitoring.

Supplementary Video 7 Point strain detection.

Supplementary Video 8 Local strain detection in a helical twist.

Supplementary Video 9 Local strain detection in a knot.

Supplementary Video 10 Fingerprint detection.

Supplementary Video 11 Washability and stability of samples in soap solution.

### Source data

Source Data Fig. 1 Statistical data.

Source Data Fig. 3 Statistical data.

Source Data Fig. 4 Statistical data.

Source Data Fig. 5 Statistical data.

Source Data Fig. 6 Statistical data.

## Data Availability

The data supporting the findings of this study are available within the article, Supplementary Information and Source data files. Source data are provided with this paper.

## Code availability

The relevant in-house codes applied in this study are available in Supplementary Information.
