# Supplementary material for: Single-step fabrication of liquid gallium nanoparticles via capillary interaction for dynamic structural colours
Source: Nat Nanotechnol. 2024 Feb 22;19(6):766–74. doi: 10.1038/s41565-024-01625-1 (PMC11186779; doi:10.1038/s41565-024-01625-1)
Supplement: Supplementary file 1 — Supplementary Figs. 1–53, discussion and Tables 1–4. [file 41565_2024_1625_MOESM1_ESM.pdf]

nature nanotechnology

Article

https://doi.org/10.1038/s41565-024-01625-1

Single-step fabrication of liquid gallium nanoparticles via capillary interaction for dynamic structural colours

In the format provided by the authors and unedited

SUPPLEMENTARY INFORMATION

TABLE OF CONTENTS

1. MECHANICAL AND STRUCTURAL CHARACTERIZATION 3

1.1 Preparation of PDMS 4

1.2 Toluene Treatment of PDMS substrates 4

1.3 Elastic Modulus of PDMS substrates: with and without Oligomers 4

1.4 Effect of oligomers of PDMS on the Gallium nanostructures 5

1.5 Liquid state of oligomers in PDMS 6

1.5.1 Oligomer-content in higher PDMS ratios 8

1.6 Liquid state of Gallium in the fabricated structure 9

1.7 Effect of temperature on the Ga nanodroplet structure 10

1.7.1 Effect of curing temperature of the PDMS 10

1.7.3 Effect of temperature after the structural color is fabricated 12

2. MATHEMATICAL MODELING OF GA NANOSTRUCTURE FORMATION ON PDMS: SUBSTRATE, GROWTH AND ENGULFING EQUATIONS 14

2.1 Nucleation and formation of droplets of critical radii 14

2.2 Initial deformation of the substrate due to Laplace pressure 14

2.3 Assumption of spherical geometry of partially immersed Gallium nanodroplets 14

2.4 Positive spreading Parameter: Engulfing of Ga droplets layer 16

2.5 Hypothesis of engulfing mechanism and Substrate-Growth-Engulfing (SGE) equations 16

2.6 Substrate Equation 17

2.7 Growth equation 18

2.8 Engulfing Equation 19

2.9 Iteration Procedure to obtain the number of immersed layers. 20

2.10 Results from SGE equations 20

2.11 Comparison of the results from SGE equations with that from the experiments 22

2.12 Prediction from the SGE equation 24

2.13 Determining structural color **25**

3. PHYSICS OF OPTICAL SPECTRA OF GA-DEPOSITED PDMS 28

3.1 Variation of the spectrum with oligomer content **29**

3.2 Mechanoresponsive on stretching the sample (uniaxial linear stretch) **31**

3.3 Study of two droplets of the same radius in an environment of PDMS **31**

3.3.1 For polarisation vector along the Gap between the spheres **31**

3.3.2 For polarisation vector perpendicular to the gap between the spheres **32**

3.4 Effect of native-oxide on Ga nanodroplets layer on its optical properties Ga-on-PDMS **35**

3.4.1 Presence of native oxide on Ga nanodroplets **35**

3.4.2 Effect of native oxide on the optical properties **35**

3.5 Structural and optical stability **36**

4. COLOR CHARACTERIZATION AND OTHER APPLICATIONS 43

4.1 CIE coordinates from Reflectivity Spectra **43**

4.2 Determination of Curvature from the image. **46**

5. NOVELTY OF THE WORK 47

5.1 Novelty in the material processing: Active Substrate **47**

5.2 Novelty in the material processing: Tuneable Plasmon-coupled Ga nanostructure **48**

6. REFERENCES 50

1. Mechanical and structural characterization

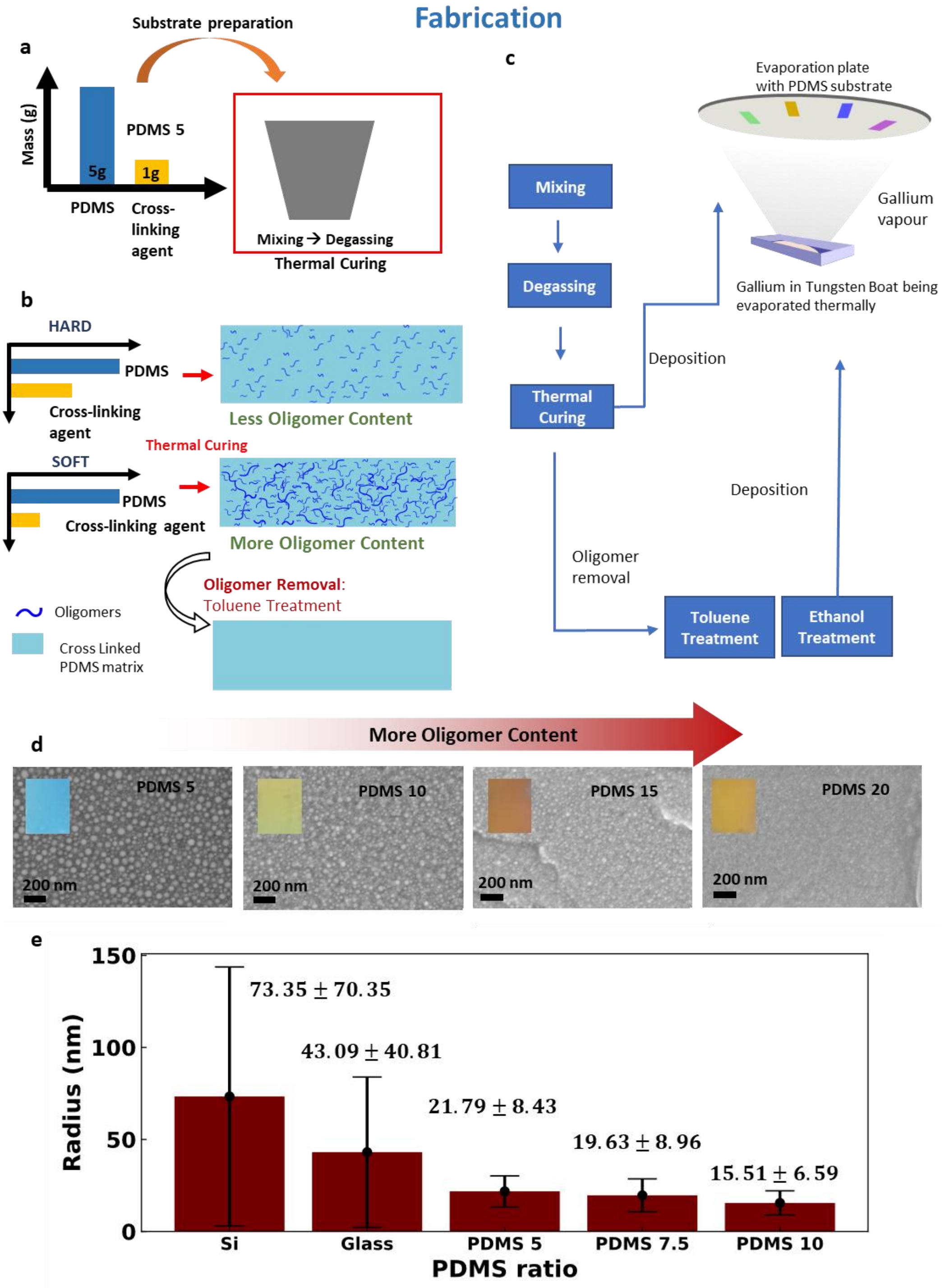

Figure 1: a, Schematic of Fabrication of different PDMS ratios. b, Schematic diagram of the PDMS substrates fabricated with different ratios of PDMS base and curing agent, and the effect of Toluene treatment on the PDMS substrate. c, Thermal evaporation of Gallium. d, The top view SEM images of PDMS ratios 5, 10, 15, 20 (from left to right). The optical images of Ga deposited on the substrates are shown in the inset. e, Average and standard deviation of radii (nm) of Ga nanodroplets formed on the substrates Si (73.35±70.35 nm, N = 350), Glass (43.09±40.81 nm, N = 290), PDMS 5 (21.79±8.43 nm, N = 908), 7.5 (19.63±8.96 nm, N = 1153) and 10 (15.51±6.59 nm, N = 742) after thermal evaporation (N is the number of nanodroplets analysed to obtain the statistics).

1.1 Preparation of PDMS

Polydimethylsiloxane (PDMS) soft substrates are prepared by mixing the liquid PDMS base and curing agent in various ratios (Dow Corning, Sylgard 184). The following notation PDMS XX represents 1 part of curing agent is added to XX parts of liquid PDMS base in weight ratio. Substrate softness increases with an increase in the proportion of liquid PDMS base. To fabricate the structural color from Ga, PDMS 5, PDMS 10, PDMS 15, and PDMS 20 are chosen as substrates. The mixture is stirred thoroughly and desiccated to remove the bubbles. The solution is poured onto a Polystyrene (PS) petri-dish and cured at 80°C in an oven for 2 hours to attain a cured soft substrate, which inherently has some uncrosslinked liquid PDMS chains. Liquid Gallium is then thermally evaporated (HHV thermal evaporator) and deposited onto these substrates to form nanodroplets. The thickness and temperature of the substrates are monitored via inbuilt thickness and temperature monitoring sensors, respectively.

1.2 Toluene Treatment of PDMS substrates

To examine the role of uncrosslinked liquid PDMS chains, cured PDMS substrates are stirred in a toluene bath for a specific duration of time. This process dissolves the uncrosslinked liquid PDMS chains since toluene acts as a suitable solvent. During this period, toluene is changed in an interval of 24 hours. Subsequently, the PDMS substrate is immersed in ethanol for 12 hours and kept in a vacuum oven at 70°C for 12 hours to remove the solvent in the sample.

1.3 Elastic Modulus of PDMS substrates: with and without Oligomers

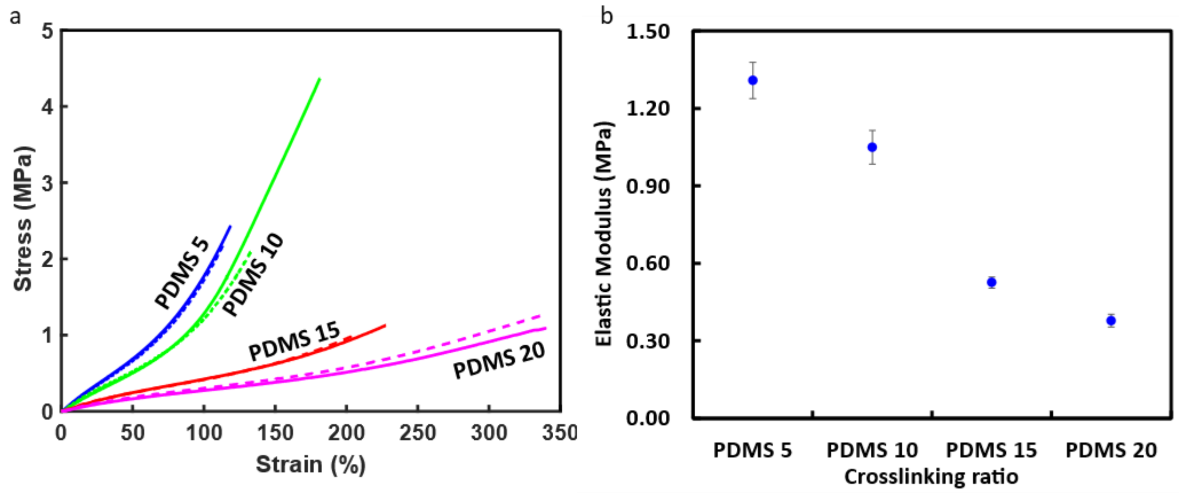

Figure 2: a, Stress-Strain plots of different PDMS samples with oligomers (solid line) and without oligomers (dotted line) subjected to a tensile strain in the Universal Testing Machine (UTM). b, Elastic modulus of substrates PDMS 5 (1.31±0.07 MPa, N = 3), PDMS 10 (1.05±0.06 MPa, N = 4), PDMS 15 (0.53±0.02 MPa, N = 4) and PDMS 20 (0.38±0.02 MPa, N = 4) measured from the stress-strain curve with upto 40% strain, where the stress and strain are linear, where N is the number of samples for which stress-strain curve was obtained.

1.4 Effect of oligomers of PDMS on the Gallium nanostructures

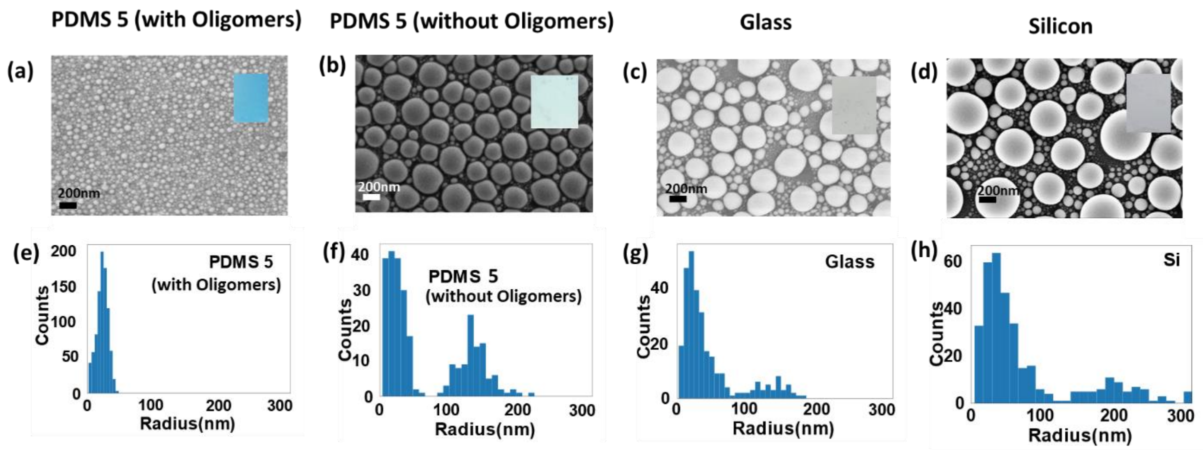

Figure 3: Top view SEM images when Ga is deposited onto (a) PDMS (with oligomers), (b) PDMS 5, (without oligomers), (c) Glass and (d) Silicon. The size distribution of Ga nanodroplets formed on the substrates (e) PDMS 5 (with oligomers), (f) PDMS 5, (without oligomers), (g) Glass and (h) Silicon.

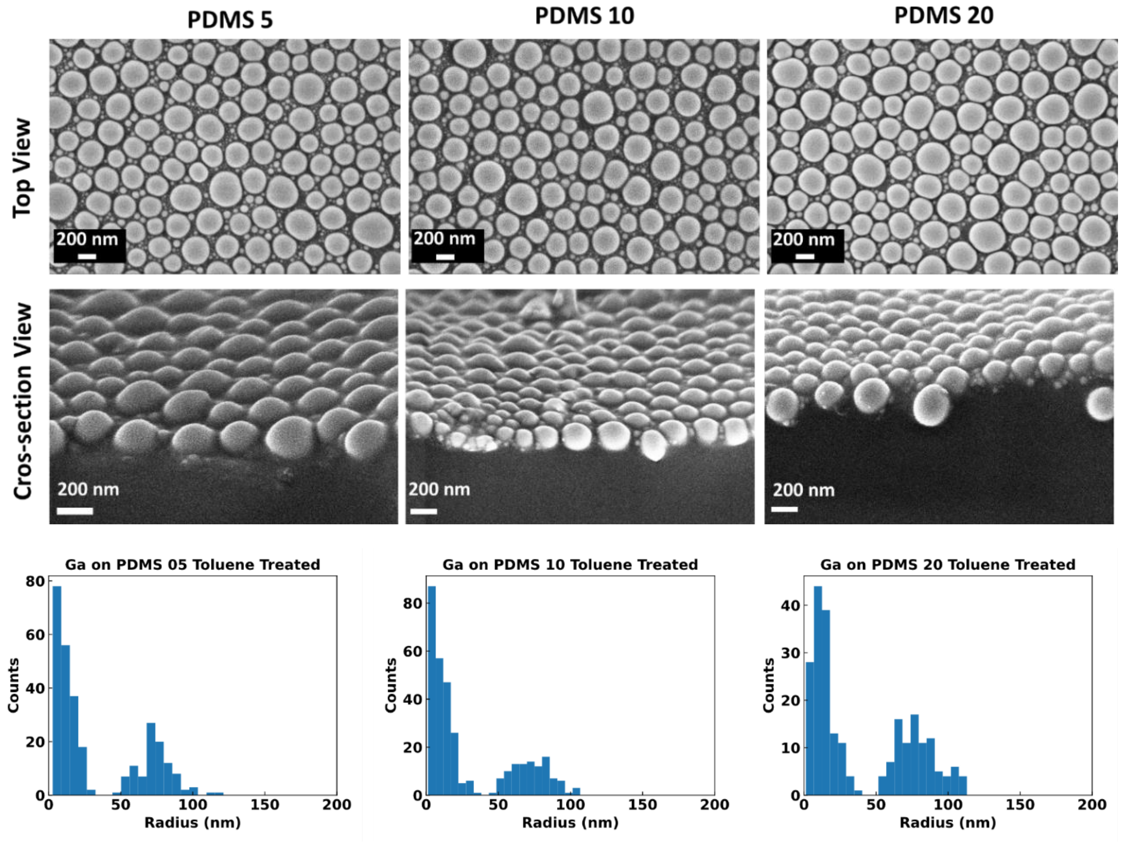

Figure 4: Top-view (top panel) and cross-sectional (middle panel) SEM images of Ga nanostructures on PDMS 5, 10, and 20 after removal of oligomers by toluene treatment and their corresponding size distribution is shown in the bottom panel.

The size distribution of Ga nanoparticles on PDMS substrates without oligomers is similar, characterized by large droplets of radii varying between 50 and 100 nm, and small droplets with radii less than 50 nm. Although Young's modulus of the substrates varies (SI Figure 2), the absence of liquid oligomer content eliminates the difference in the size distribution of the Ga nanodroplets. This establishes that the fluidic interaction between the liquid oligomers and liquid Ga droplet results in a layered nanodroplets structure, thus producing colors.

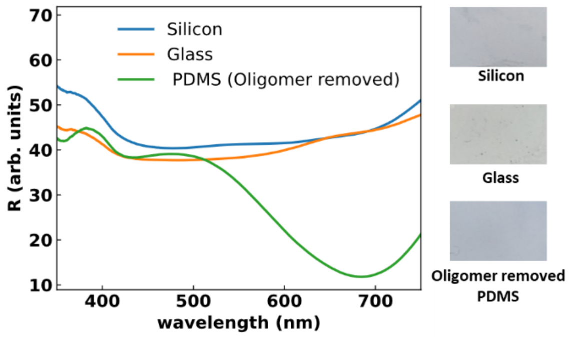

Figure 5: Reflectivity spectra of Ga deposited Si, Glass and Toluene treated PDMS. (Right column) Optical images of Ga deposited simultaneously on Si, glass and PDMS substrates without oligomers (Toluene treated).

Thus, we conclude that removing oligomers eliminates the distinction between different PDMS ratios. Therefore, oligomers play a crucially significant role in the determination of nanostructures.

1.5 Liquid state of oligomers in PDMS

The oligomers of the PDMS that encapsulate the Ga droplets are in liquid state and remain liquid even after Ga droplets get enclosed. To verify this Gallium deposited samples were treated with Toluene to remove the oligomers. We observe the absence of the Gallium layer, leaving behind the transparent PDMS substrate, as shown in Figure 6. Figure 6a is the optical image of the Gallium deposited samples, whereas the toluene treatment removed the oligomer layer, which was holding the Gallium nanodroplets, thereby making the sample transparent.

The structural morphology exhibited by Ga nanostructures are explained by the fluidic interactions between the oligomers and Gallium (detailed in SI section 2). The following experimental trends are a result of capillary forces between the two liquids, Gallium and liquid oligomers:

1. Radius of Gallium decreases with oligomer content.
2. Number of layers of Gallium increases with oligomer content.
3. Radius of Gallium decreases with depth.

The depth into which the droplets can embed depends on the oligomers available in the bulk, and the relative ease at which the oligomers can migrate to the Gallium-PDMS interface for engulfing the nanodroplets. Figure 6c and 6d shows that PDMS 20 and 30 have a total cross-section of 139nm and 215nm, drastically different given that the same thickness of Ga was deposited. The sizes of Gallium nanodroplets at the innermost depths are similar, with a diameter of order less than 10 nm. However, since the amount of oligomers is more in PDMS 30, we have a large number of layers when compared with PDMS 20. The fact that the droplets are immersed in the oligomers (Figure 6e,

6f) is confirmed by Energy Dispersion Spectroscope (EDS) images, as shown below in Figure 6g. The oligomers are extracted to the top from the bulk by the capillary forces at the interface of the oligomers and Ga droplet.

Nevertheless, the oligomers will be present in bulk as well, due to the following reasons:

(1) the Gallium nanodroplet layers cannot extract the oligomers entirely owing to the slowing migration rate with an increase in the number of nanodroplet layers
(2) The number of Ga nanodroplets are insufficient to extract out all the oligomers from the bulk.

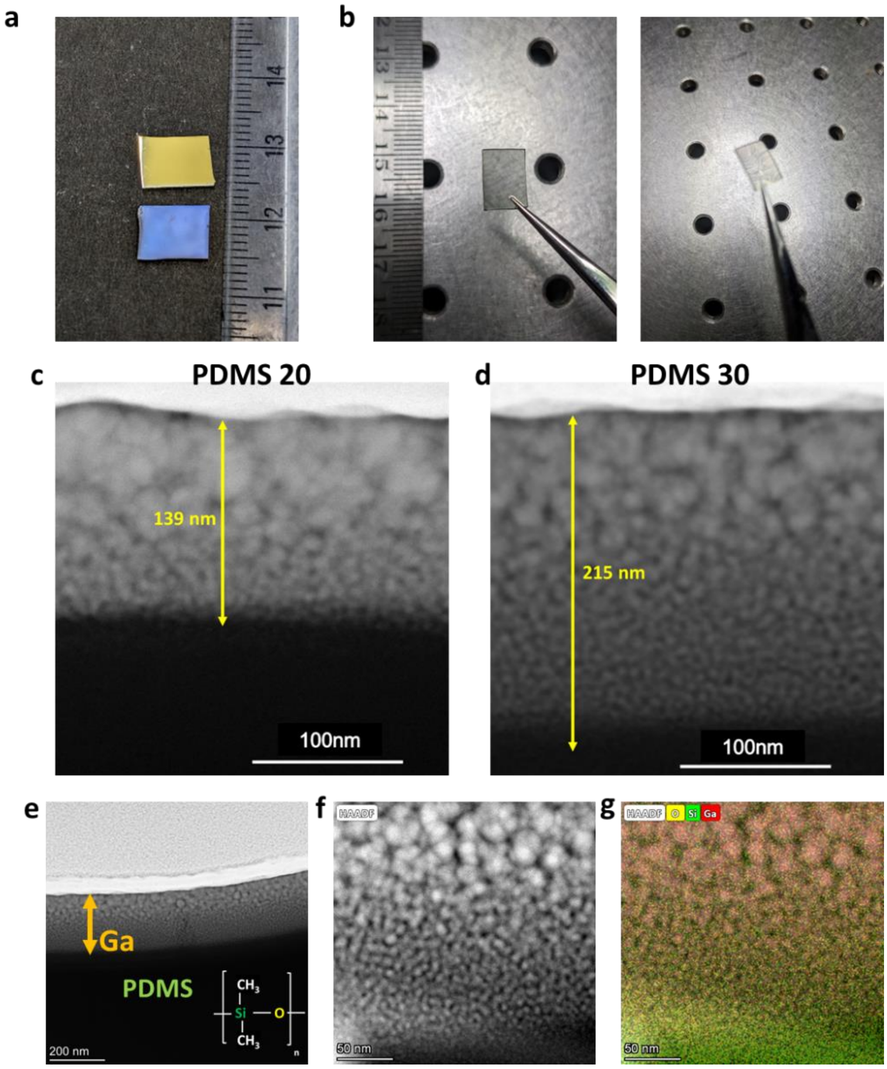

Figure 6: Effect of toluene treatment on Ga-deposited PDMS samples. a, Gallium deposited PDMS 05 (yellow) and 10 (blue), b, Optical images of the sample after toluene treatment. High Angle Annular Dark Field (HAADF) images of Ga deposited on (c) PDMS 20 and (d) PDMS 30. e, HAADF image at low magnification showing the Gallium nanodroplet layers with the substrate. f, HAADF image at high magnification, showing only the Gallium nanodroplet layer. g, EDS mapping of Oxygen, Silicon and Gallium. Oxygen, shown in yellow, and Silicon, shown in green indicate the presence of PDMS and oligomers.

From this, we conclude that the PDMS on the surface is in the liquid state, however, viscous enough to hold the layer of Gallium nanodroplets.

1.5.1 Oligomer-content in higher PDMS ratios

The color of the sample gets more and more saturated as the PDMS ratio (oligomer-content) increases. This can be clearly seen in the samples shown in Figure 2.

PDMS 40:1 and 50:1 amount to 2.5 % and 2.0 % of the curing agent in the PDMS base, respectively. This results in quite less curing and leaves a significant amount of oligomers uncured. For a significant change in resulting color, a considerable difference in the amount of oligomers needs to be there, as explained below:

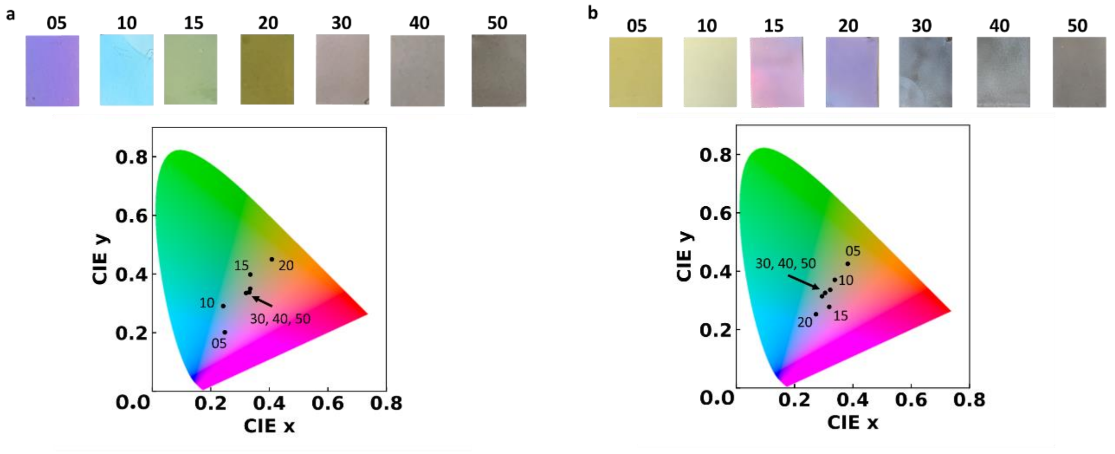

Figure 6h | Optical images of Ga deposited on PDMS 5, 10, 15, 20, 30, 40 and 50 under identical conditions. a, Deposition of 100nm Ga onto the substrate. b, Deposition of 150nm Ga onto the substrate.

As the Gallium nanodroplets contact the substrate, the PDMS cross-linked matrix with liquid oligomers infused in it, the droplet is stopped by the force exerted by the PDMS cross-linked matrix. The rate of this engulfing determines the time for which the surface area of the droplet is exposed to Gallium vapor. If the oligomer chains are short, they will be more mobile and less viscous, thus making the process of engulfing faster. For example, if we consider PDMS 30 and PDMS 5, the former will have shorter oligomers with higher mobility and a faster rate of engulfing. Since the engulfing rate is faster, the time for which the droplets are exposed to the Ga vapor is smaller than that in the case of PDMS 5. Hence, the growth of the droplets in PDMS 30 ceases faster. Hence, they are of smaller sizes as compared to PDMS 5. But when we consider PDMS 40 and PDMS 50, the proportion of the curing agent tends to come closer in these substrates. So, the relative sizes of the nanodroplets are similar in higher PDMS ratios. The difference between the nanodroplet sizes in higher PDMS ratios would not be significant enough for a visual change in structural color. Therefore, the higher PDMS ratios exhibit similar chromaticity when deposited with Ga under identical conditions.

As depicted in Figure 6h, the PDMS 30, 40, and 50 colors do not have significant differences. We observe a wide variation of color when PDMS substrate contains a lower proportion of PDMS base, such as PDMS 5 and PDMS 10.

1.6 Liquid state of Gallium in the fabricated structure

During the deposition process, the chamber is at a temperature of 35°C or more, which is above the melting point of Ga (30°C). This leads us to conclude that the Ga is in a liquid state during fabrication. Another signature implying the liquid state of Ga is that the droplets are in spherical shape, the most stable hydrodynamic shape configuration. While there are reports of Gallium being in a liquid state well below its melting point [1,2] the following results confirms the supercooling effect oof Ga in our samples as well.

Had Gallium been in a solid state, it would have been in a crystalline state, the signature of which would be there in selected area electron diffraction (SAED) pattern taken from a droplet as discrete points. However, as shown in Figure 7, we observe a diffused ring in the SAED pattern depicting absence of crystallinity. From this we conclude that Gallium nanodroplets are in liquid state.

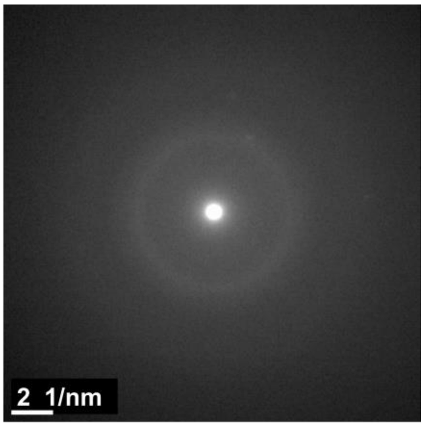

Figure 7: Selected Area Electron Diffraction (SAED) Pattern taken from Gallium nanodroplet.

Differential scanning calorimetry, wherein a small drop of bulk Gallium (89mg) was put in the crucible, indicates that Gallium exists in liquid state supercooled state till -15°C. As we still lower the temperature, the temperature of the crucible along with Gallium increased (see Figure 8a) suddenly, due to a sudden release of heat (see Figure 8b) as Ga changed its phase from liquid to solid. The small time interval indicates that the solid state of Ga is energetically more favourable, due to the crystallinity of its solid phase. We thus conclude that Gallium nanodroplets are in a liquid state as the temperature at which we conducted our experiments was at 25°C.

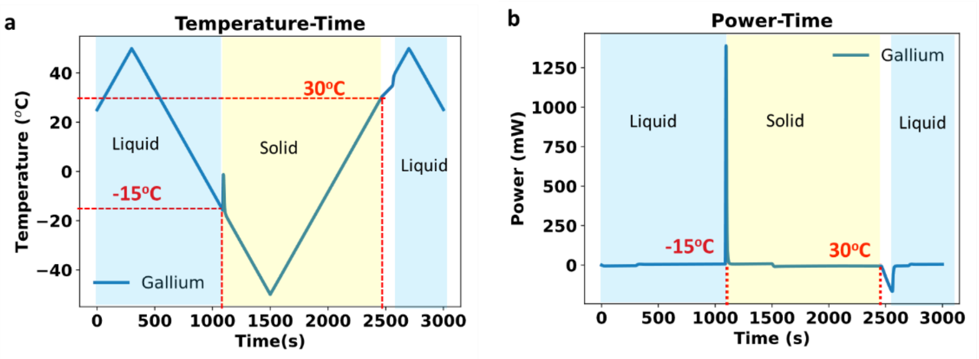

Figure 8: Differential scanning calorimetry data of Gallium. a, Temperature of Gallium as a function of time. b, Power released from Gallium with respect to time.

1.7 Effect of temperature on the Ga nanodroplet structure

the following sets of experiments to understand the effect of temperature on the formation of chromogenic Ga nanodroplet structures. We investigated the following effects of temperature:

i) Effect of curing temperature of the PDMS, during its preparation which affects the rate of cross-linking.
ii) Effect of temperature of the substrate during the deposition of Ga onto PDMS, which affects the growth rate of Ga nanodroplets and hence effects their size.
iii) Effect of temperature after the structural color is fabricated and is sample is exposed to the environment.

1.7.1 Effect of curing temperature of the PDMS

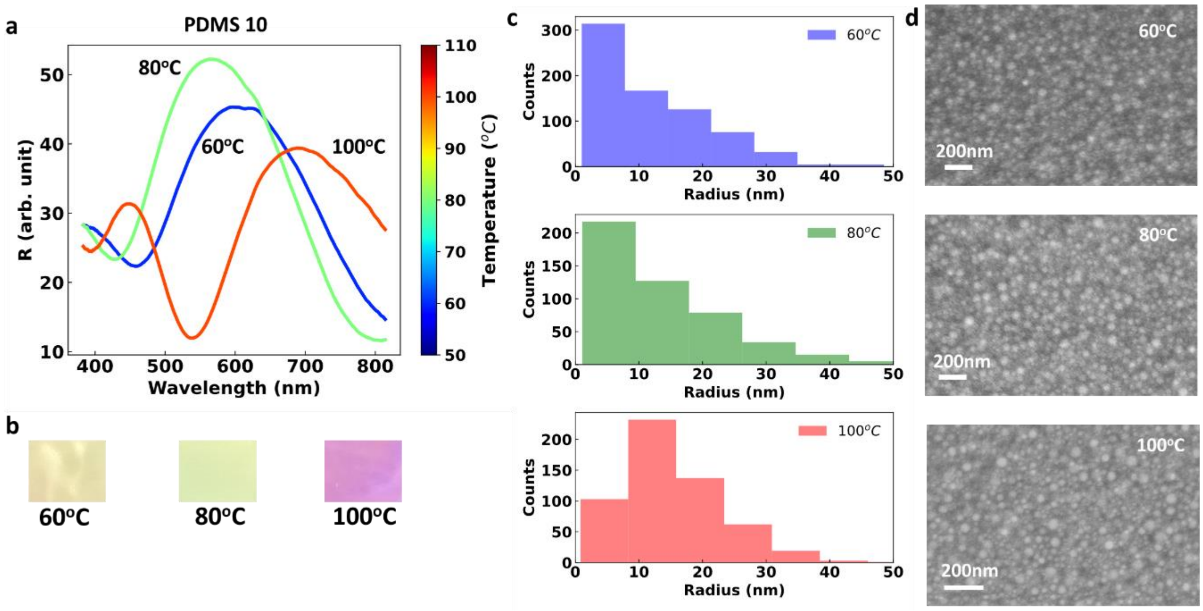

Figure 9: Effect of curing temperature of the PDMS 10 substrate. a, Reflectivity spectra of Ga deposited PDMS 10 which was cured for 2 hours at 60°C, 80°C and 100°C. b, Optical images of the Ga deposited PDMS 10 samples. c, Radii distribution of to Ga nanodroplets as viewed from the (d) top-view SEM images of PDMS 10 cured at 60oC, 80oC and 100oC.

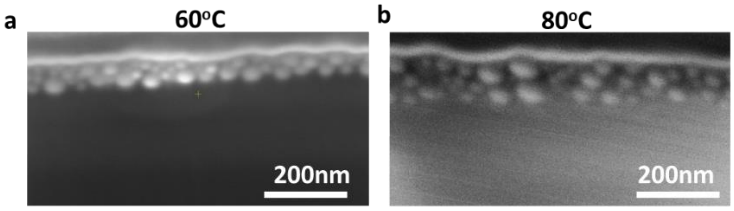

Figure 10: Cross-section SEM images Ga-deposited PDMS cured at (a) 60°C and (b) 80°C

To study the effect of temperature on cross-linking of the PDMS, we cured PDMS 10 for 2 hours at temperatures 60°C, 80°C, and 100°C respectively. The samples thus-prepared were deposited under identical conditions, simulaneously on the same evaporation plate. The reflectivity spectrum Figure 9a obtained the three samples are different, indicating different optical color as shown in Figure 9b. Figure 9c is the size distribution of the top-view SEM image of the corresponding samples, as shown

in Figure 9d. With increase in cross-linking ratio, we observe an increase in the size of the nanodroplets, as shown in Figure 9c. The drastic difference in colors exhibited is a result of structural change, resulted due to difference in cross-linking density due to curing temperature. The variation in sizes of Ga nanodroplets is more clearly observed in the cross-section view (Figure 10). Curing PDMS at higher temperature resulted in larger Ga-nanodroplet sizes.

1.7.2 Effect of temperature of the substrate during the deposition of Ga onto PDMS

The temperature of the substrate during the process of deposition results in blue-shift of the reflectivity spectrum and, hence a color change. Figure 11a, 11b and 11c correspond to the different deposition parameters and substrate, each showing a similar trend of blue shift with reflectivity spectrum.

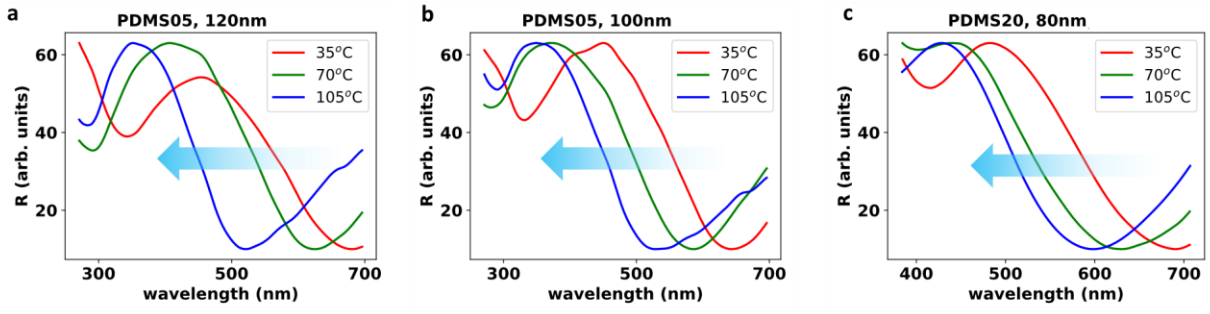

Figure 11: Reflectivity spectra of the samples for different parameters | a, PDMS 5, 120nm Ga. b, PDMS 5, 100nm Ga. c, PDMS 20, 80nm Ga.

With the increase of this temperature results in the reduction of the growth rate, because the Gallium atoms tend to be in vapor phase. Thus, the sizes of Ga nnaodroplets will be smaller, as depicted in the following figure. Since scattering blue-shifts with the size of scatterers, a blue shift in the scattering is expected as long as the number of layers of Ga nanodroplets are same in the samples.

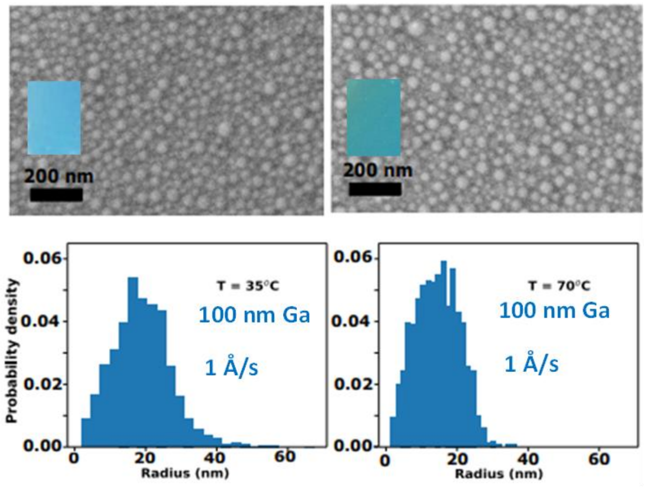

Figure 12: Size distribution of Ga nanodroplets as obtained from view image of samples deposited under identical conditions but different deposition temperatures 35°C (left) and 70°C (right).

In the previous case where the cross-linking density changes, a trend was not observed due to variation in the number of layers of Ga nanodroplets, although there is a small variation sizes as viewed from top.

1.7.3 Effect of temperature after the structural color is fabricated

To check the reversibility of the device with respect to heat cycles, we heated to fabricated samples to a particular temperature and then cool back to room temperature for optical measurements. The overlapping reflectivity spectra of the samples as shown in the following figure, and similar values of Chromaticity coordinates as shown in the table below, shows the reversibility of the device.

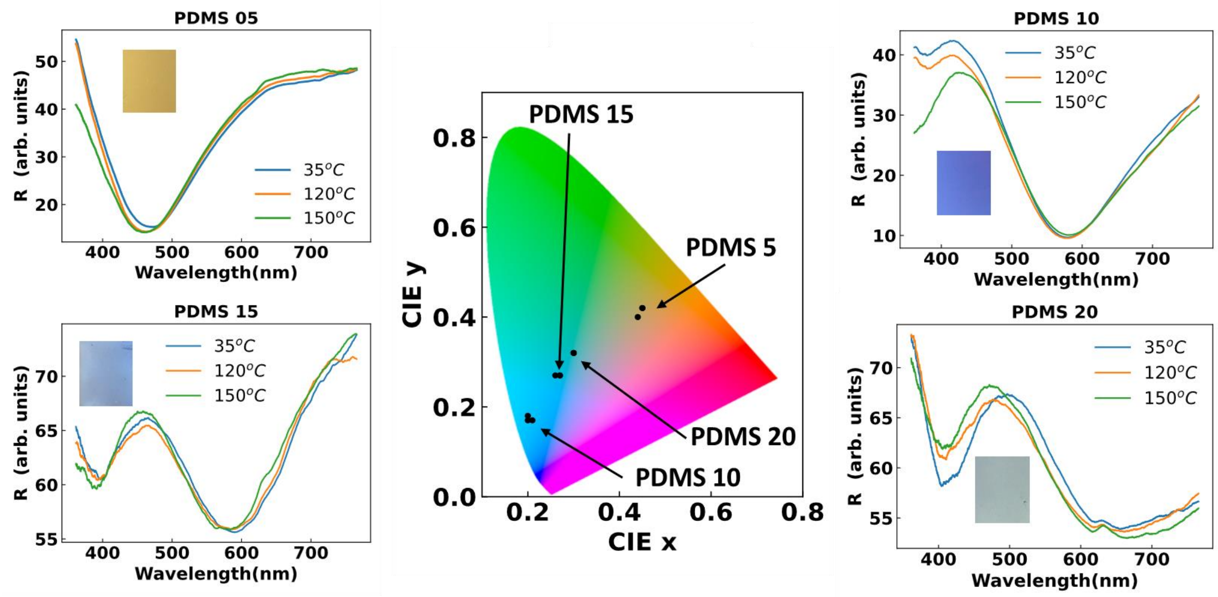

Figure 13: *Experiment to show the reversibility with temperature. The reflectivity of the samples were measured after one cycle of heating to the labelled temperature and cooling back to the room temperature. The fact that the chromaticity coordinates do not change indicates stability of the device.*

Temperature	PDMS 5		PDMS 10		PDMS 15		PDMS 20	
(°C)	CIE x	CIE y	CIE x	CIE y	CIE x	CIE y	CIE x	CIE y
35	0.44	0.40	0.20	0.17	0.26	0.27	0.30	0.32
120	0.45	0.42	0.21	0.17	0.27	0.27	0.30	0.32
150	0.45	0.42	0.20	0.18	0.27	0.27	0.30	0.32

Table 1: Chromaticity coordinates of the samples obtained at room temperature after one heating cycle.

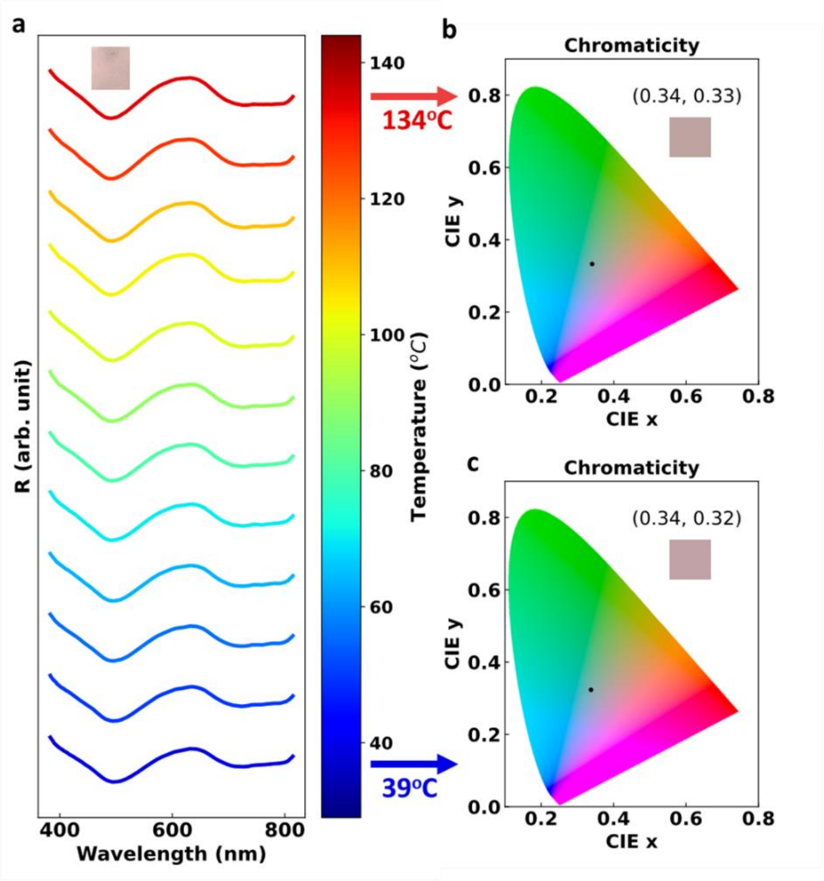

Figure 14: a, Reflectivity spectrum of the sample as a function of temperature. The inset is the optical image of the sample. b, Chromaticity of the sample at 134°C. The color of the inset is the color corresponding to the chromaticity coordinate obtained from the reflectivity spectrum. c, Chromaticity of the sample at 39°C. The color of the inset is the color corresponding to the chromaticity coordinate obtained from the reflectivity spectrum.

The samples also show, stability with respect to temperature during the heat cycle as well, as shown in the Figure 14, as shown below. The reflectivity spectra of the sample does not change its spectral features with temperature, and the chromaticity coordinates remain similar both at higher (Figure 14b) and lower (Figurer 14c) ends of temperature.

2. Mathematical modeling of Ga nanostructure formation on PDMS: Substrate, Growth and Engulfing Equations

In this section, we hypothesize the formation mechanism of Ga nanospheres and their embedding into the PDMS. Also, we propose a set of differential equations based on a few assumptions to model the experimentally observed size distribution trends of Ga nanodroplets.

2.1 Nucleation and formation of droplets of critical radii

Thermal evaporation of Gallium onto the substrate results in Ga vapors near the PDMS substrate, which condenses to form nanospheres. The surface tension of Ga renders the droplets to be spheres. The formation of a Liquid Ga-air interface costs energy proportional to the surface area of the sphere, whereas condensation into liquid droplets releases its vaporisation energy proportionate to its volume. Therefore there would be a critical radius which would be the minimum radius of Ga nanodroplets that form upon condensation. Once the Ga nanodroplets form, they grow due to further condensation of Ga vapor to the liquid droplet of a few nanometers radius before it comes into contact with the PDMS substrate.

2.2 Initial deformation of the substrate due to Laplace pressure

When the nanodroplets come in contact with the substrate, the initial speed it and its Laplace pressure will result in the substrate's initial deformation, leading to a small immersion of the nanodroplet into PDMS. Once infinitesimal immersion happens, the contact line of Ga droplet and PDMS occurs, and the following sequence of events occur.

Young's law governs the wetting of droplets on rigid substrates and Neumann's triangle on liquid substrates, which is infinitely soft[3]. In between the rigid and liquid substrates called the soft solids, the contact angle for larger droplets follows Young's law, whereas, with the smaller droplets, the contact angle is obtained by the balance of Neumann's triangle. In soft solids, the balance between capillarity and elastic forces is governed by the elastocapillary length scale $l_{el} = \frac{\gamma_{Ga}}{E}$, where γ_{Ga} is the surface tension of the gallium droplet, and E is Young's modulus. For the magnitude of radius $R > l_{el}$, ridge formation at the contact line is small, and macroscopically droplets follow Young's law like a rigid substrate. Whereas for small droplets $R < l_{el}$, high Laplace pressure inside the droplet can deform and form a dimple on the substrate. In such cases, the droplet profile takes the shape of a liquid lens which is given by the balance of γ_{Ga}, Υ_{PDMS} and $\Upsilon_{PDMS-Ga}$. The elastocapillary length for a Ga droplet on PDMS 5 is 496. During typical evaporation, the radius of the Ga droplet is observed to be around 25 nm. Hence the droplet will resemble the case of a liquid lens on PDMS substrates.

2.3 Assumption of spherical geometry of partially immersed Gallium nanodroplets

The Ga droplet on PDMS can be considered to be placed equivalently on a liquid substrate from the elastocapillary length scale analysis (Sec 2.2). As a result, the shape of a Ga droplet on a PDMS substrate is governed by the balance of interfacial stresses (surface tension forces) at the three-phase contact line, which is well characterized by Neumann's triangle[4]. The balance of forces in horizontal and vertical directions gives,

$$\gamma_{Ga} \cos\alpha + \gamma_{Ga-PDMS} \cos\beta - \gamma_{PDMS} \cos\sigma = 0 \qquad (1)$$

$$\gamma_{Ga} \sin\alpha - \gamma_{Ga-PDMS} \sin\beta + \gamma_{PDMS} \sin\sigma = 0 \qquad (2)$$

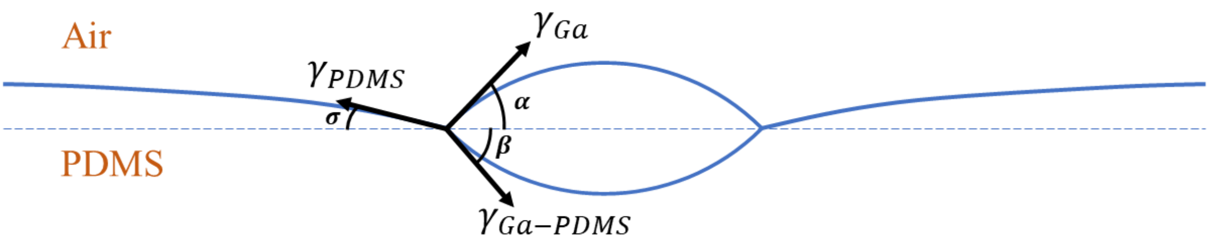

Figure 15: Schematic of Ga droplet on PDMS substrate for very high elastocapillary length compared to the droplet radius.

From the cosine law of the triangle, relation between the angle made by the different liquid with horizontal and surface energies can be expressed as,

$$\alpha + \beta = \cos^{-1}\frac{\gamma_{PDMS}^2 - \gamma_{Ga-PDMS}^2 - \gamma_{Ga}^2}{2\gamma_{Ga}\gamma_{Ga-PDMS}} \tag{3}$$

$$\alpha + \sigma = \pi - \cos^{-1}\frac{\gamma_{Ga-PDMS}^2 - \gamma_{Ga}^2 - \gamma_{PDMS}^2}{2\gamma_{Ga}\gamma_{PDMS}} \tag{4}$$

From the geometrical analysis, the volume occupied by Ga with the Gallium-air and PDMS-Gallium interfaces can be calculated as,

$$V_1 = \frac{\pi R_d^3}{3\sin^3\alpha}(1-\cos\alpha)^2(2+\cos\alpha) \tag{5}$$

$$V_2 = \frac{\pi R_d^3}{3\sin^3\beta}(1-\cos\beta)^2(2+\cos\beta) \tag{6}$$

Equating the volume in the Ga of occupied with two different arcs of radius to the volume of the initial spherical droplet $V_1 + V_2 = V$, contact radius R_d can be found. With the contact radius, radius of the lens formed by the Ga droplet can be determined by,

$$R_1 = \frac{R_d}{\sin\alpha} \tag{7}$$

$$R_2 = \frac{R_d}{\sin\beta} \tag{8}$$

Since the surface energies of Ga-air and Ga-PDMS interface are close, the exposed and immersed radius of curvature is almost the same. For example, for a Ga droplet of 200 nm diameter, the exposed and immersed radii are 102.4nm and 100nm. This enables us to assume a spherical geometry of Ga nanodroplet even during immersion into the PDMS.

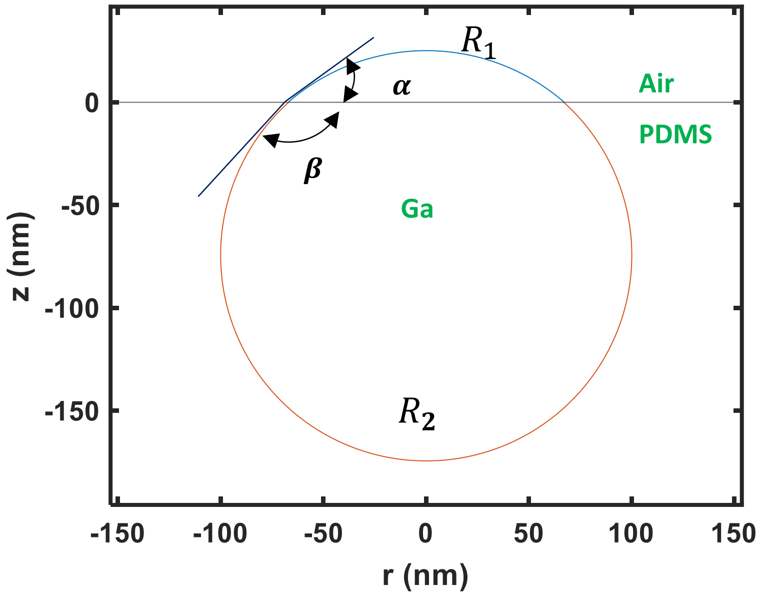

Figure 16: The shape of the Ga droplet (200 nm diameter) on the PDMS substrate is calculated analytically. Gravity is neglected at the nanoscale, leading to equal pressure across both air and PDMS phases and hence the curve remains a flat line between both phases. From Neumann equilibrium conditions, contact angles are obtained, $\alpha = 40.8°$ and $\beta = 138°$. Using the angles and volume conservation, the radius of the spherical arcs are $R_1 = 102.4\ nm$ and $R_2 = 100\ nm$.

2.4 Positive spreading Parameter: Engulfing of Ga droplets layer

The phenomena of fluid separation and ridge formation occur when materials with a positive spreading parameter (defined in the manuscript) are used to swell PDMS[5,6]. The spreading parameter determines the minimum energy configuration of three interfaces. For our case, the spreading parameter S turns out to be

$$S = \gamma_{Ga-air} - \gamma_{Ga-oligomers} - \gamma_{oligomers-air} = 650 - 590 - 20 = 40\ mN/m \qquad (9)$$

Here γ_{Ga-air}, $\gamma_{Ga-oligomers}$ and $\gamma_{oligomers-air}$ denote the surface tensions between Ga and air, Ga and oligomers, and oligomers and air. A positive spreading parameter of 40mN/m implies that the surface energies of Ga-air interface is 40 milliJoule per square meter more energy than that of the two interfaces Ga-oligomers and oligomers-air, combined. To minimize the surface energy of the system (for a positive spreading parameter), the oligomers will separate from the PDMS network and cloak the Ga nanodroplets. The mechanism of Gallium nanodroplets penetrating the PDMS is hypothesized here using the result for positive spreading parameter and a few assumptions made about the substrate and its interaction with Ga nanodroplets. The positive spreading coefficient ($S > 0$) of the system results in the extraction of oligomers from the cross-linked PDMS network and tends to cloak the nanodroplet. During engulfing, the nanodroplet pulls the oligomers to cloak it up. The surface undulations of the oligomers are energetically unfavorable as compared to a planar surface. Therefore, the surface tends to be planar, thus extracting more oligomers out of the cross-linked PDMS network. Moreover, the initial velocity of the nanodroplet into the PDMS contributes to their immersion.

2.5 Hypothesis of engulfing mechanism and Substrate-Growth-Engulfing (SGE) equations

Engulfing of the Ga nanodroplet is an interplay of the following three events co-occurring.

a) Separation of Oligomers from the PDMS network

Let f be the volume of liquid oligomers present in a given substrate volume in units of nm^3. The rate at which f changes when a Ga droplet-oligomer interaction takes place dictates the phenomena of oligomer separation and will be described by the expression $\frac{df}{dt}$.

b) Growth of Ga droplet

The rate of increase of radius R (in units of nm) Ga nanosphere describes its growth and is depicted by the expression $\frac{dR}{dt}$.

c) Engulfing of Ga nanodroplet

We define the engulfing angle θ (radian) as the polar angle of the contact line Ga-air-PDMS interface if one assumes the center of the sphere to be the origin and positive z-axis as the normal of undeformed PDMS-air interface from PDMS to air (SI Figure. 8). The rate $\frac{d\theta}{dt}$ describes the engulfing of Ga-droplet.

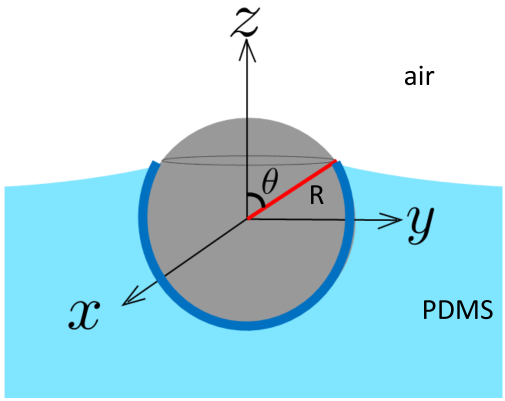

Figure 17: A partially immersed and engulfed Ga nanodroplet with an engulfing angle θ. Note that the exposed surface area is $S = 2\pi R^2(1 - \cos\theta)$ and that immersed is $2\pi R^2(1 + \cos\theta)$. When $\theta = 0$, it is the case of complete immersion. A completely immersed nanodroplet cannot grow, and hence the rate of change of radius vanishes. No,te that $S = 0$ at $\theta = 0$, indicating complete immersion. At $\theta = \pi, S = 4\pi R^2$, the entire surface is exposed and hence the rate of increase of radius is maximum.

In the following sections, we present the proposed model of these events in terms of differential equations. This set of differential equations describes the substrate-droplet interaction of a single nanodroplet.

2.6 Substrate Equation

Oligomers possess fluidic properties and are embedded homogeneously in the PDMS network. When a droplet touches the PDMS, the Ga-air-PDMS interface is formed and oligomers tend to diffuse from the bulk PDMS to the droplet interface and engulf it. This requires migration of the oligomers to the interface. Note that, at a given instant f is the total volume of oligomers present in the PDMS network available for migration towards interface and contribute to droplet-engulfing. The unit of f is that of volume (nm^3). The volume of oligomers used to engulf the nanodroplet will no longer be available in the PDMS network. We assume that a thin layer of thickness w nm of oligomers will engulf the nanodroplet in the

immersed area uniformly. We hypothesize that the rate at which f will deplete is proportional to the volume of oligomers left in the PDMS network after an engulfing of θ is,

$$\frac{df}{dt} \propto f - 2\pi R^2 w(1 + \cos\theta)$$

The migration of oligomers toward the interface occurs due to the contact force and we assume it to be proportional to $2\pi R \sin\theta$,

$$\frac{df}{dt} \propto 2\pi R \sin\theta$$

With κ_s as the proportionality constant, we obtain

$$\frac{df}{dt} = -\kappa_s\big(f - 2\pi R^2 w(1 + \cos\theta)\big)(2\pi R \sin\theta) \tag{10}$$

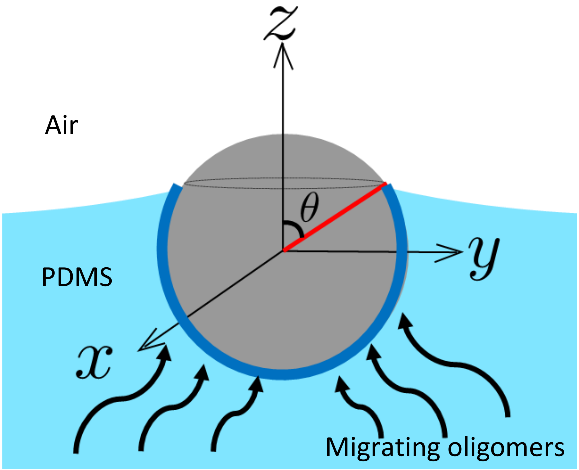

Figure 18: Schematic of oligomers migrating from the PDMS network to the interface of PDMS and Gallium. The Ga nanodroplet will be cloaked by a thin liquid oligomer layer.

Here κ_s is known as substrate constant and describes the ease of migration of oligomers through the PDMS network. It is a dimensional constant with units $nm^{-1}s^{-1}$. It does not vary during the process of engulfing because the cross-linked network is assumed not to change during engulfing. The negative sign on the right-hand side signifies the reduction of oligomers as they are used up for engulfing.

Intuition dictates that higher PDMS ratios (those with a lower proportion of curing agent) have a more volumetric fraction of oligomers in a given substrate volume and hence will be characterized by higher values of f.

2.7 Growth equation

The conversion from vapor to liquid occurring at the exposed surface area results in a change in the volume of the nanodroplet. Let ρ be the density of liquid Gallium (5.9 g/cc), and J the flux of liquid Ga entering the droplet through condensation. We can therefore write,

$$\rho\frac{d}{dt}\left(\frac{4}{3}\pi R^3\right) = 2\pi R^2(1 - cos\,\theta)J \tag{11}$$

or,

$$\frac{dR}{dt} = \frac{J}{2\rho}(1 - cos\,\theta) \tag{12}$$

or,

$$\frac{dR}{dt} = \kappa_\rho(1 - cos\,\theta) \tag{13}$$

Here $\kappa_\rho = \frac{J}{2\rho}$ is the growth constant with unit $nm\,s^{-1}$.

The growth constant is determined by the flux of Ga atoms condensing into the droplet, which depends on temperature and the density of Ga atoms in the vapor phase. One can experimentally control the number density of Ga atoms in vapor phase by manipulating the deposition rate during thermal evaporation. More is the number density of Ga-atoms in the vapor phase, the faster the condensation into the liquid droplet phase.

Temperature plays a crucial role in determining the vapor-liquid equilibrium of Ga. A higher temperature favors the vapor phase, and hence the rate of condensation decreases, which causes a reduction in J and a lowering of the growth constant κ_ρ.

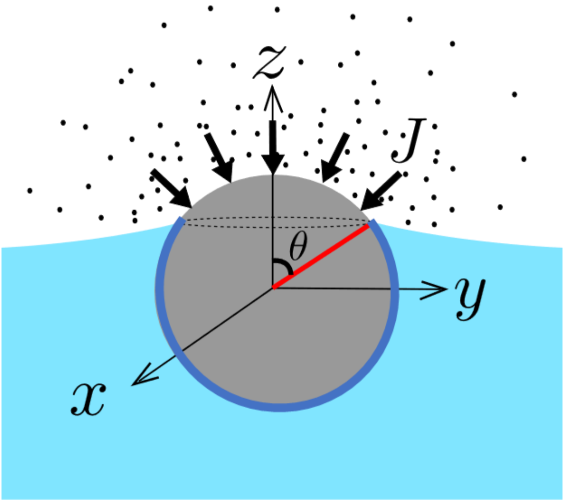

Figure 19: Schematic of the Ga vapor liquefying at the interface of Ga vapor and nanodroplet. The flux of Ga atoms condensing into the droplet is directly proportional to the number density around the exposed surface of the Ga droplet. With the increase in temperature, the probability of Ga atoms being in a vapor state increases, thus reducing the flux of condensing Ga atoms.

2.8 Engulfing Equation

The initial immersion due to the Laplace pressure of the Ga droplet renders the initial value of θ to be $\pi - h$, where $h \ll 1$. As the engulfing occurs, its value decreases towards $\theta = 0$, which is the case of complete engulfing.

We hypothesize that the rate at which engulfing occurs is proportional to the amount of oligomers available for migrating towards the interface and contributing to engulfing. Thus, we can write

$$\frac{d\theta}{dt} = -\kappa_e\big(f - 2\pi R^2 w(1 + \cos\theta)\big) \quad (14)$$

where κ_e is the engulfing constant with unit $nm^{-3}s^{-1}$.

2.9 Iteration Procedure to obtain the number of immersed layers.

The substrate-growth-engulfing (SGE) equations describe the substrate droplet equation for a single droplet. The boundary conditions for the equations are defined by θ, from infinitesimal engulfing ($\theta = \pi - h$) to complete engulfing ($\theta = 0$). During this process, f decreases and the radius R of the nanodroplet increases. There are ample oligomers for engulfing the initial layers (the deepest layer observed in the SEM image). The rate of engulfing would be faster for the droplets in these layers; hence the growth time is relatively less than the droplets from the top layer. Here the engulfing phenomena dominate, and complete engulfing takes place. For the subsequent layers of droplets, the amount of oligomers is depleted, thus reducing the rate of engulfing and increasing the growth time. For the topmost layer, the growth time dominates the engulfing time and partial engulfing occurs.

To model the above picture, we solve the SGE equations for a nanosphere with an arbitrary but reasonable choice of initial radius and a thickness of oligomer coating around it. The initial volume of oligomers f is chosen reasonably as well. The values of SGE constants κ_s, κ_e and κ_ρ, decide complete or partial engulfing. If complete engulfing occurs, the initial value of f for the next layer will be the final value of f in the case of a most recent engulfed droplet. Once the partial engulfing occurs, we stop the iteration and infer the number of iterations required to obtain the partial engulfing, which is the number of layers of Ga nanodroplets formed in the PDMS.

2.10 Results from SGE equations

On imposing the condition that the immersion angle is monotonically non-increasing with time, solutions of the SGE differential equations for a single droplet determine whether complete immersion will occur. The value of R at $\theta = 0$ (complete engulfing) is the final radius of the droplet.

To demonstrate an example, let us consider a PDMS substrate parametrized by $f_0 = 3000\,nm^3$ and substrate constants $\kappa_s = 0.3nm^{-1}s^{-1}$, $\kappa_\rho = 500nm\,s^{-1}$ and $\kappa_e = 0.1\,nm^{-3}s^{-1}$. We choose the initial radius of the droplet $R_0 = 4nm$ and the thickness of liquid oligomers engulfing it to be $w = 1nm$. The choice of the initial infinitesimal engulfing is chosen to be $\theta = \pi - h$ with $h = 10^{-4}rad$. While solving the SGE equations numerically, we deliberately impose θ to be monotonically decreasing and non-negative. Boundary values that satisfy these conditions for θ result in physically acceptable solutions.

1st layer

The first iteration of the numerical solution of SGE equations results in the monotonic decrease of θ from infinitesimal engulfing ($\theta = \pi - h$) to complete engulfing ($\theta = 0$). During this time, the radius of the nanodroplet increases from 4nm to 9.9nm, and the volume of oligomers decreases from $f = 3000nm^3$ to $f = 2691nm^3$.

The radius of the Ga droplet for the first layer is 9.9nm. The remaining volume of oligomers, $f = 2691nm^3$ will be used as the initial oligomer volume f_0 for the next iteration.

:

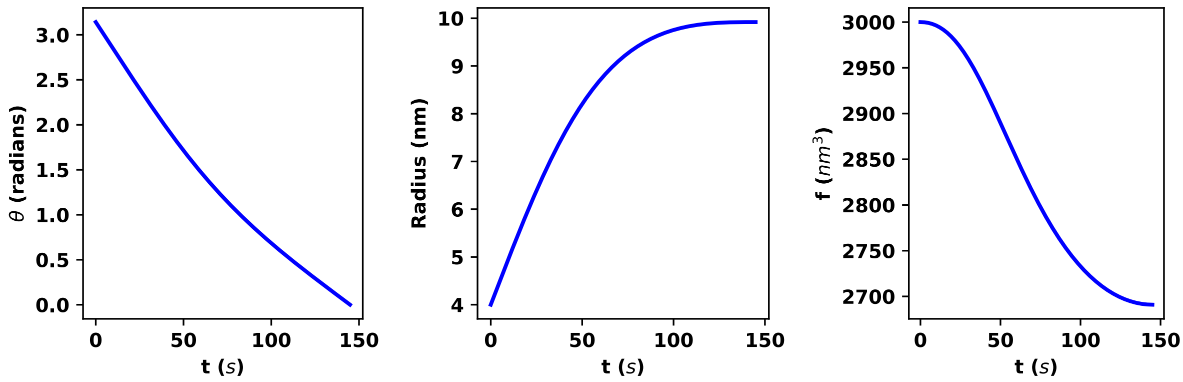

Figure 20: Graphs of (left) engulfing angle θ vs time t. (center) Radius (R) of Ga in the first layer vs. time t. (right) Volume of oligomers f vs. time t. These are the solutions of SGE equations for the first iteration. One can infer that complete engulfing occurs here, leading to the final radius of the Ga nanodroplet being 9.9nm.

2nd layer

The second iteration also results in complete engulfing, with a final Ga nanodroplet radius of 10.9 nm. Note that the radius is greater than that obtained in the previous layer. The remaining volume of oligomers $f = 2360nm^3$ is used as the initial oligomer volume for the next iteration.

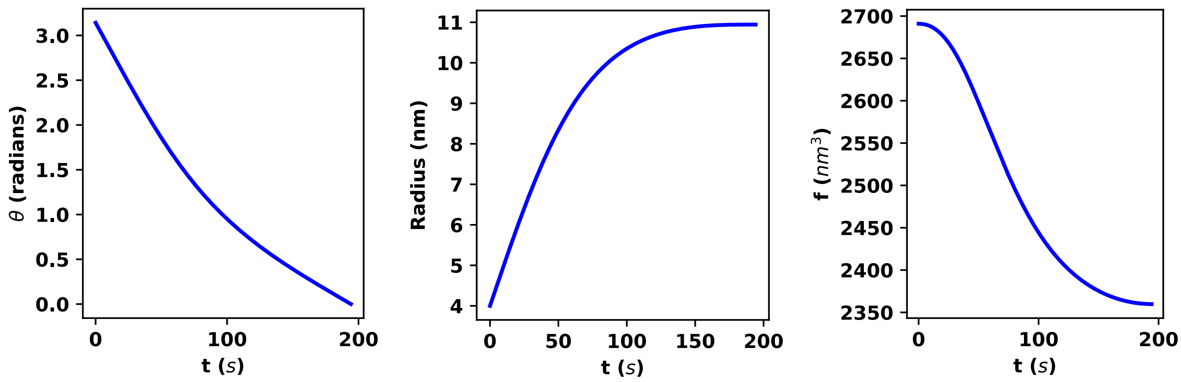

Figure 21: Graphs of (left) engulfing angle θ vs time t. (center) Radius (R) of Ga in the first layer vs. time t. (right) Volume of oligomers f vs. time t. These are the solutions of SGE equations for the second iteration. One can infer that complete engulfing occurs here, leading to the final radius of the Ga nanodroplet being 10.9nm.

3rd layer

In the third iteration, the final engulfing angle is $\theta = 0.6\, rad = 34^o$, thus indicating an incomplete immersion. This would be the topmost layer of the Ga droplets deposited onto the PDMS. The droplets at this layer have a radius of 13.3 nm, which is larger than the radius of droplets in the immersed layers.

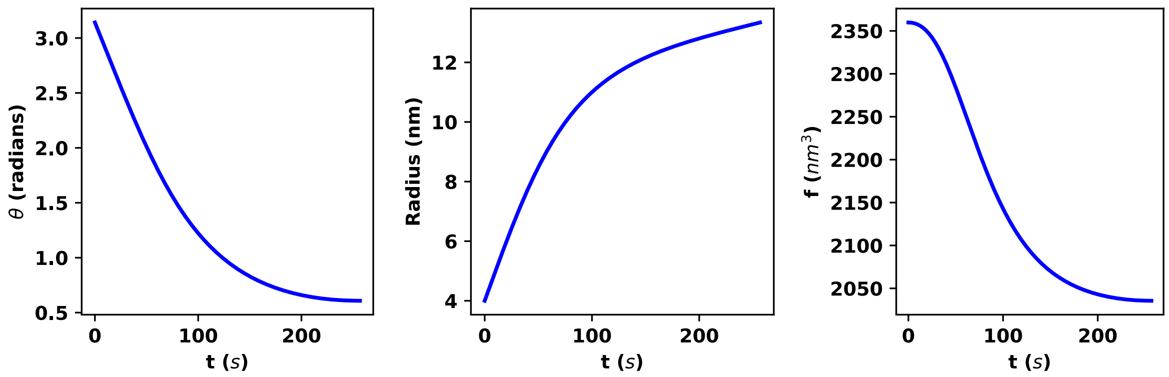

Figure 22: Graphs of (left) engulfing angle θ vs time t. (center) Radius (R) of Ga in the first layer vs. time t. (right) Volume of oligomers f vs. time t. These are the solutions of SGE equations for the third iteration. One can infer that incomplete engulfing occurs here. Hence it would be considered the topmost layer. The radius of the Ga particle formed here is 13.3 nm.

Thus, we obtain the depth profile of the Ga nanodroplet radius, as shown in the following figure.

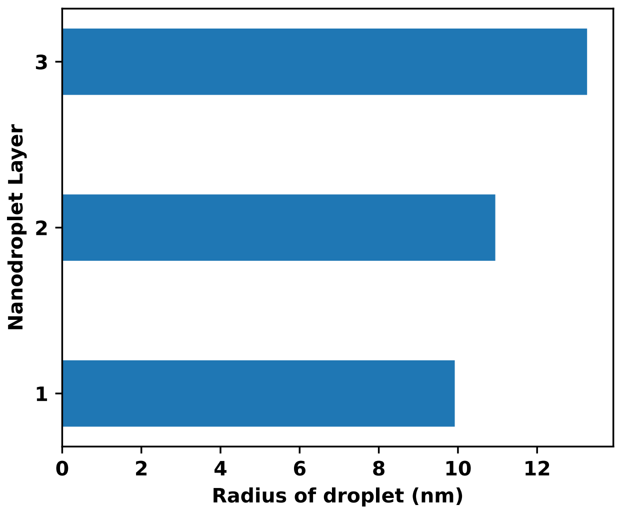

Figure 23: Histogram depicting the depth profile of Ga-radius across the cross-section. The deepest layer (1st layer) is the smallest in size. The size gradually increases with a decrease in depth. The topmost layer is of the highest radius. This trend matches with the observed cross-sectional images from SEM.

2.11 Comparison of the results from SGE equations with that from the experiments

As shown in the following figure, for each size distribution as observed in the cross-section images, there exist parameters of SGE equations which, when used to solve, give the matching results. The parameters used for the following three SEM images are tabulated as follows.

A note about the trend of parameters which gives the matching results for observed SEM images, are in order. The growth parameter $\kappa_\rho(= J/2\rho)$ is the same for all the PDMS substrates because the three samples were deposited simultaneously. The number density of Ga atoms in the vapor and the density of Ga are the same for all substrates during a particular

deposition. The initial radius is chosen arbitrarily but is the same for all three substrates because it is formed before the droplet encounters the substrates; hence it is independent of the substrate.

The substrate constant κ_s increases with the PDMS ratio. It measures the ease of movement of oligomers through the PDMS network. It is physically reasonable to argue that a higher PDMS ratio implies less cross-linking, making it easier for the oligomers to move through the PDMS network. More availability of oligomers means a faster engulfing, which explains the increasing trend of engulfing constant κ_e with PDMS ratio. The thickness of liquid oligomers encapsulating the nanodroplets is inferred to increase with the PDMS ratio from SGE equations. This can be consistently accommodated by the assumption that there is ample supply of the oligomers in higher PDMS ratios and hence a more increased thickness oligomer cloaking is favorable.

Parameters	PDMS 5	PDMS 10	PDMS 20
$\kappa_s (nm^{-1}s^{-1})$	0.4	0.5	0.8
$\kappa_p (nm\ s^{-1})$	1280	1280	1280
$\kappa_e\ (nm^{-3}s^{-1})$	0.1	0.11	0.15
$f_0 (nm^3)$	4500	5000	7000
$R_0\ (nm)$	5.0	5.0	5.0
$w\ (nm)$	0.5	0.7	2.0

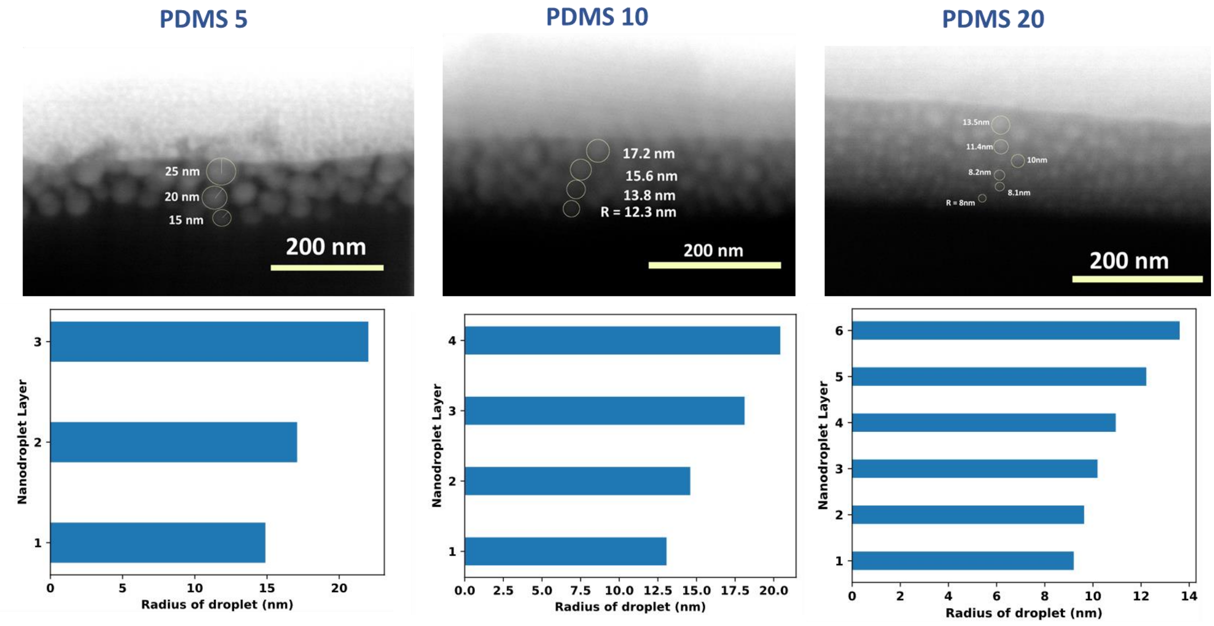

Figure 24: (Top) Table showing the parameters used for substrate-growth-engulfing (SGE) equations, matching the modeling results with the experiment's results. (Middle) The SEM images with representative circles are drawn around Ga nanodroplets to determine their radii. (Bottom) Results from SGE equations are solved using the parameters tabulated above. One can infer a fair agreement with the results from SGE equations and the observed SEM images.

2.12 Prediction from the SGE equation

The SGE equations have successfully explained the size distribution trends as observed experimentally. Once we have found the parameters for a particular deposition process and the PDMS substrates, we can use them and relate them with the experimental parameters to obtain a control on size distribution. For example, the rate of deposition and temperature will affect the growth constant κ_ρ. With the increase in the rate of deposition and decrease in temperature, κ_ρ increases. Though the exact analytical expression relating κ_ρ to these experimental parameters have not been determined yet, we can investigate the size distribution trend of varying them by changing the value of κ_ρ in SGE equations.

For example, consider PDMS 10, with SGE parameters obtained from SEM image analysis. The following figure depicts the effect of increasing the rate of deposition, which is effectively modeled by increasing the value of κ_ρ. We expect to obtain nanodroplets of larger sizes when we increase the deposition rate or lower the temperature. In the cross-section, the number of layers reduces.

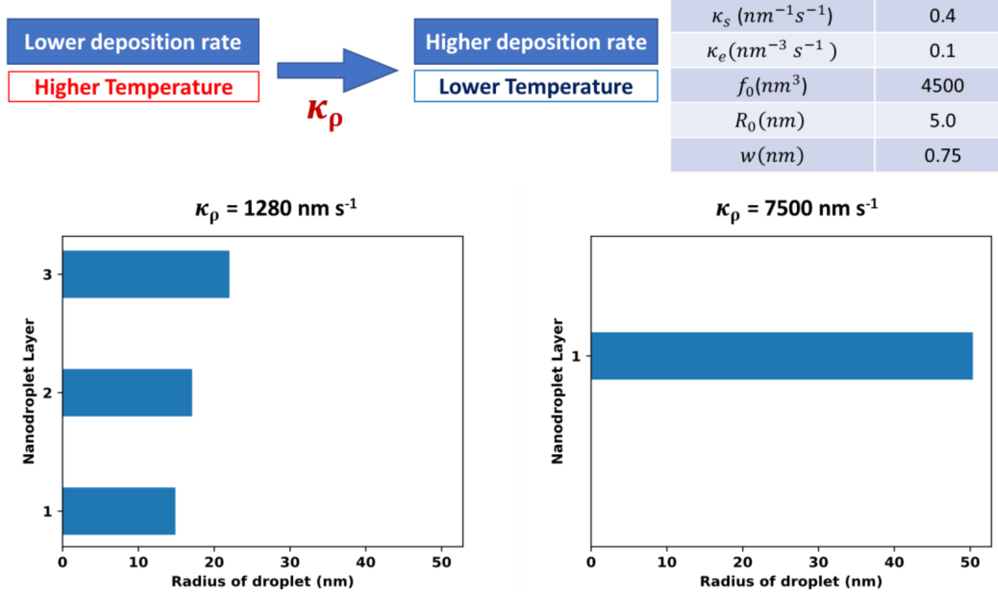

SGE Parameters	PDMS 5
$\kappa_s\ (nm^{-1}s^{-1})$	0.4
$\kappa_e(nm^{-3}\ s^{-1})$	0.1
$f_0(nm^3)$	4500
$R_0(nm)$	5.0
$w(nm)$	0.75

Figure 25: The predicted trend of Ga-radius deposited on PDMS with varying rates of deposition or temperature. The table shows the parameters for PDMS 10 obtained from an SEM image of the sample deposited at the rate of 1Å/s (and $\kappa_\rho = 1280\ nm/s$, the leftmost SEM image). On increasing the deposition rate or lowering the temperature, we expect to get less number of Ga layers and nanodroplets of larger sizes.

Experimentally we observed that increasing the deposition rate to 7 Å/s from 1 Å/s decreased the number of layers of Ga nanodroplets. Moreover, the cross-sectional radius of Ga nanodroplets measured from the cross-section SEM image (see Figure 15) for lower rate deposition is 20.4±3.5 nm, while the higher rate of 7 Å/s is 49.9 ± 7.7 nm. This observation is in tandem with the predictions from SGE equations.

PDMS 5, 100 nm, 35 deg C

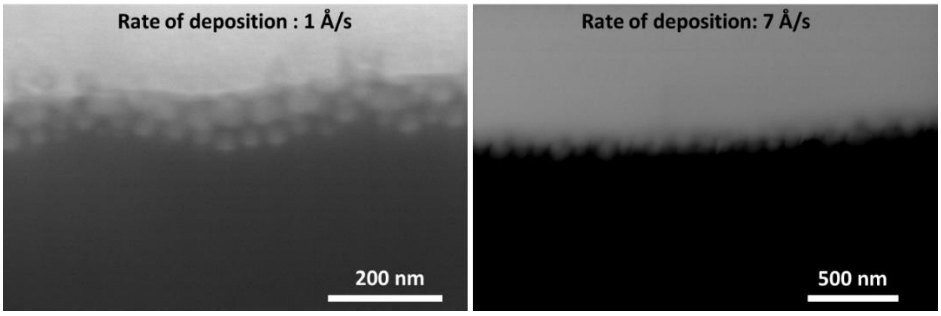

Figure 26: Cross-sectional Image of Ga deposited on PDMS 5 at 35 deg C with deposition rate (left) 1 Å/s, and (right) 7 Å/s.

The PDMS substrate is usually characterized by the ratio of the PDMS base to the cross-linking agent. The SGE equation provides another useful PDMS characterization utilizing SGE parameters. These parameters, as demonstrated in the previous section, describe substrate interaction with the gallium nanodroplets. It would be a novel method to describe the PDMS properties and their behavior with the Ga-deposition parameters.

2.13 Determining structural color

The structure-to-colour relation is many-to-one. The equations come into usefulness in determining the structure and thereby the color, in terms of chromaticity coordinates as shown below in Figure 6. Having determined the number of layers and SGE parameters for a deposition process, one can input the corresponding structure in an FDTD program and determine the chromaticity. In the following, we demonstrate, that the SGE parameters (determined in SI section 2.11) allowed us to determine the structure, whose reflectivity is obtained by FDTD method. The chromaticity obtained from the resulting reflectivity matches with those observed experimentally.

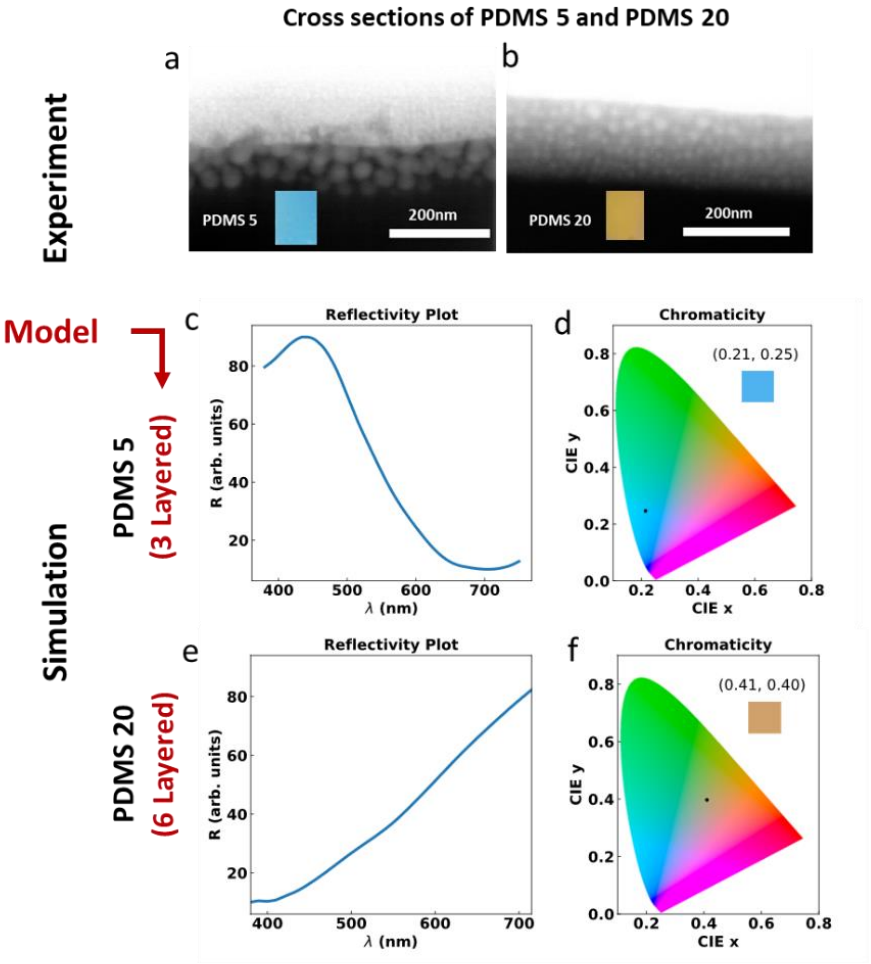

Figure 27: Model used to determine the color. The experimentally obtained cross-section images of (a) PDMS 5 and (b) PDMS 20. Inset shows the optical image of the real samples. c, Reflectivity plot obtained from the structure predicted by the SGE model for PDMS 5 and the corresponding (d) chromaticity coordinates. e, Reflectivity plot obtained from the structure predicted by the SGE model for PDMS 5 and the corresponding (f) chromaticity coordinates.

An outlook on the model

The molecular sizes of the short-length oligomers exhibit a statistical variation. The properties of the oligomers in the PDMS cross-linked matrix such as its mobility and viscosity are dependent mostly on the distribution of these molecular sizes, and the extent of cross-linking of the bulk-PDMS. Experimentally it is difficult to determine these properties, considering the diffiulty of extracting the oligomers from the PDMS bulk as it is. The toluene treatment removes the oligomers from the bulk, but it is extracting the oligomers back from the toluene solution is an experimental challenge. Thus, the properties of the oligomers elude direct experimental determination.

Through the proposed model, one can fit the parameters to match the cross-section z-profile of the radii distribution of the Ga nanodroplets and attribute the parameters as a label to the PDMS. It will be useful, if a microscopic model could be developed pertaining to the dynamics of the oligomers and connect it with the phenomenological and empirical model we propose here. Our model is a step towards connecting the microscopic model from the available experimental data we have gathered.

3. Physics of Optical spectra of Ga-deposited PDMS

By examining the reflectivity spectra of Ga-deposited PDMS, we can experimentally verify how light interacts with nanodroplets and gain insights into the morphology of Ga on PDMS. The results from SEM images confirm that the fluidic interaction of Gallium with the liquid oligomers of PDMS forms layers of Ga nanodroplets with varying radii. Due to encapsulation by oligomers, there will always be a dielectric Gap of refractive index 1.4 between the Ga nanodroplets. Its refractive index determines the optical properties of Ga. The real and imaginary parts of the refractive index of Ga are shown in the following figure[7].

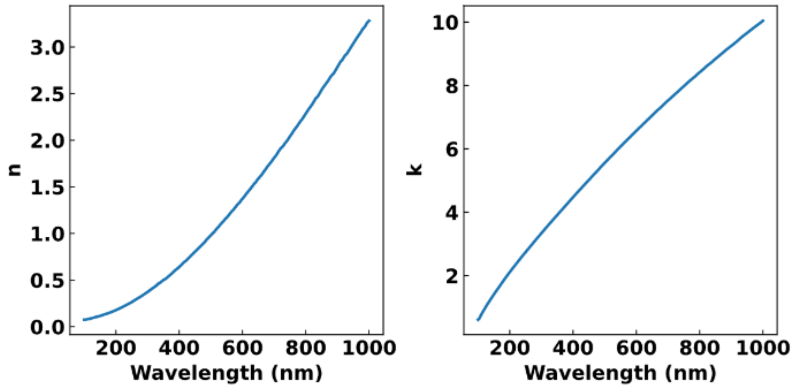

Figure 28: The real (left) and imaginary (right) part of the refractive index of Gallium.

Scanning electron microscopy shows that the structure of Ga nanodroplets on PDMS has the following structural characteristics:

1) The Gallium nanodroplets form several layers of nanodroplets, depending on the oligomer content of the PDMS. The more oligomer content more will be the number of layers.
2) The sizes of the Ga nanodroplets decrease with the depth of the Ga nanolayer into the PDMS.
3) An encapsulation of a thin layer of liquid oligomers separates each Ga nanodroplet.
4) For a given layer, the spatial location of Ga nanodroplets is random.

With the above constraints, we simulated some of the structures in Lumerical, a commercial FDTD software and observed a good match with the experimental reflectivity spectra.

FDTD was used with periodic boundary conditions along the x and y directions to simulate a large area of randomly distributed Ga nanostructures. The x-span and y-span were made large enough to cover a statistically significant number of Ga nanodroplets in each layer. Nevertheless, the periodicity would cause discrepancies between the experimentally obtained and the simulated reflectivity spectra. After achieving a satisfactory correlation between the experimental and simulated reflectivity spectra, the next step is comprehending the patterns observed in the reflectivity spectrum.

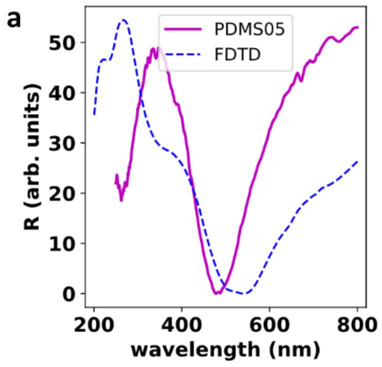

Figure 29: Lumerical simulation of FDTD region and the experimentally obtained reflectivity spectra of (a) PDMS 5, (b) PDMS 10 and (c) PDMS 15.

3.1 Variation of the spectrum with oligomer content

We observe that the spectral features of the reflectivity spectrum red-shift with an increase in the oligomer content (Figure 19). The observation is counter-intuitive given that higher oligomer content results in smaller particles; hence, one would expect a blue-shift. However, the electromagnetic fields in the inter-layer spatial region contribute to the spectrum, thus resulting in a red shift.

Although the sizes of Gallium nanodroplets decrease with the increase in the PDMS substrate's oligomer content, the spectral features consider the number and depth of the Ga nanodroplet layers. The structure with larger Ga nanodroplet layer depth results in red-shifted spectral features.

The intensity plots below depict the interaction of the incident electromagnetic field with different layers of Ga nanodroplets. At lower wavelengths, the electromagnetic field can interact with only the topmost layer of Ga spheres (see Figure 20g). In contrast, the higher wavelength fields can interact with all the layers of the structure (see Figure 20h and 20i).

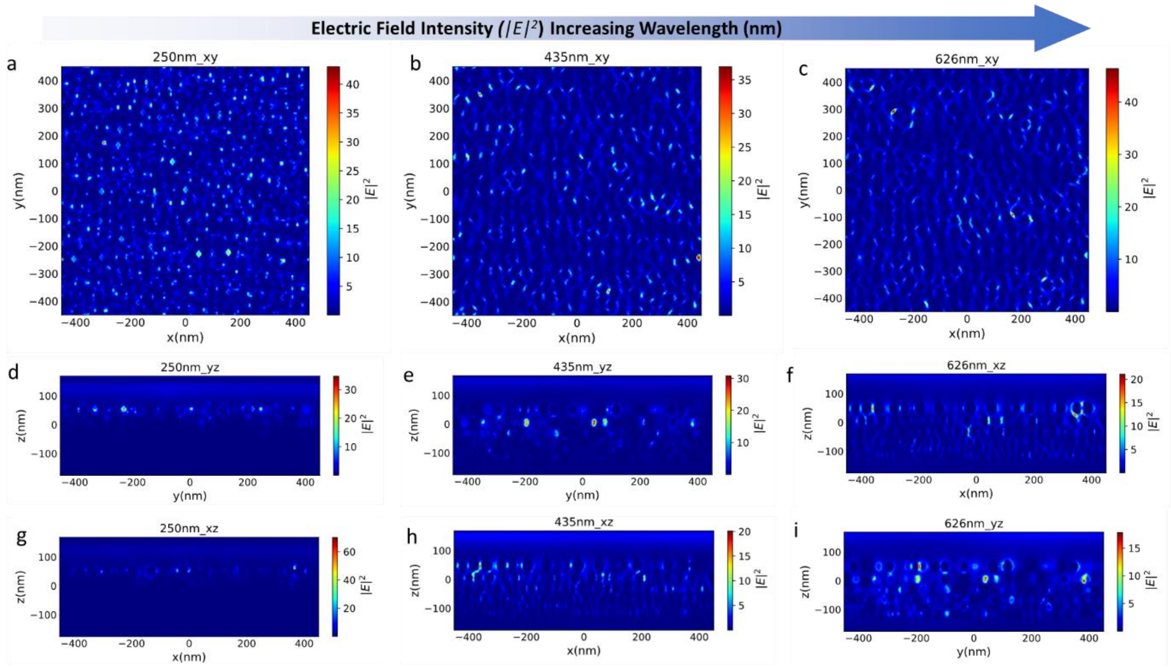

Figure 30: Electric field intensity ($|E|^2$ with units (V^2/m^2)) plots at UV (250nm) and visible (435nm and 626nm) regions for the six-Layered structure of PDMS 15. (a-c) The Intensity plots in the XY plane of the first layer of Ga nanodroplets. (d-f) Field intensity at YZ plane. (g-i) Field intensity at XZ plane.

The following plot depicts the electric field intensity at some localized regions between two different layers of the Gallium nanodroplets. The incident electric field effectively sees a structure of feature size of the order of depth of the excited layers. Therefore the system in which the total thickness of Gallium droplet layers is larger will exhibit spectral features at larger wavelengths.

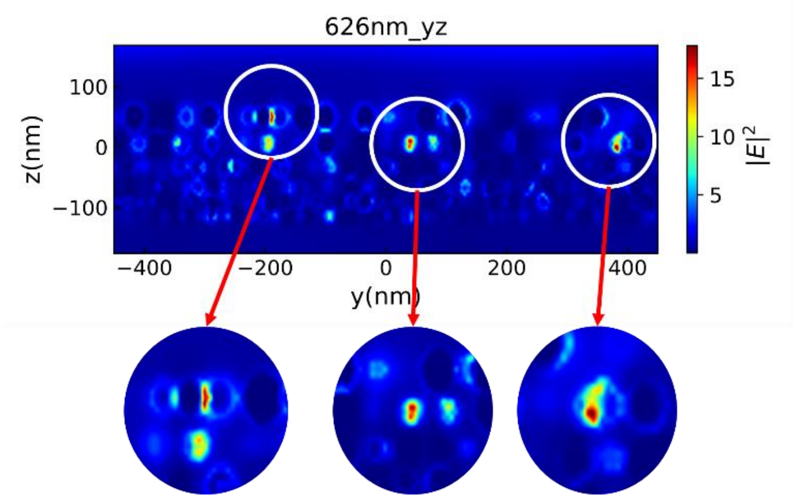

Figure 31: Electric field intensity ($|E|^2$ with units (V^2/m^2)) plots at some localized regions between two different layers of the Gallium nanodroplets. The inter-layer field interaction causes the light to interact with the droplets in all the layers, thus equivalent to the interaction of light with an object of effective length scale equal to the thickness of the Ga embedded region.

The SEM image shown below reveals that the depth of the Ga nanodroplets is higher in PDMS 20. Since it has more layers of Ga nanodroplets, gap plasmon contribution from all the layers of PDMS 20 will occur at a wavelength higher than that in the case of PDMS 5. Therefore, the major spectral features of PDMS 20 are expected to be red-shifted compared to PDMS 5.

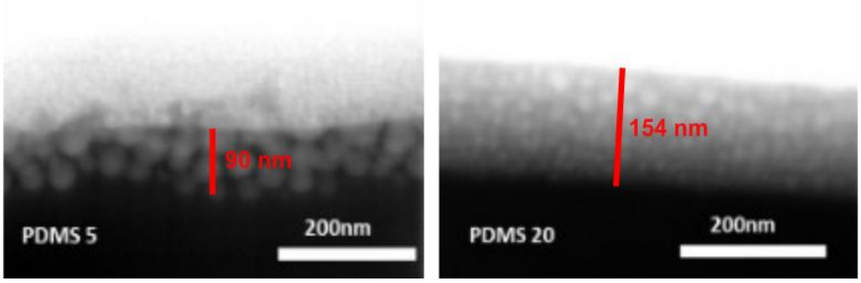

Figure 32: The depth of Ga nanodroplets in the case of PDMS 20 (more oligomer content) is more than that of PDMS 5 (less oligomer content).

3.2 Mechanoresponsive on stretching the sample (uniaxial linear stretch)

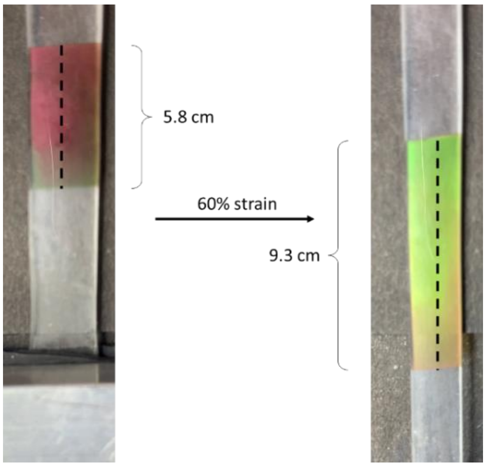

Figure 33: Change of color of Ga deposited PDMS, occurring due to a strain of 60%.

The change of color of the sample is attributed to the change in the chromogenic structure of Ga nanoparticles interacting with the incident light. For two adjacent particles in a given layer, stretching will increase the inter-droplet gap filled with PDMS oligomers with a refractive index of 1.4. Hence, the study is necessary to determine the interaction of electromagnetic fields with two Ga droplets in PDMS placed near each other with varying gaps.

3.3 Study of two droplets of the same radius in an environment of PDMS

For finite difference time domain (FDTD) simulations, the commercial software package Lumerical is used. The two spherical spheres are placed close to each other with a particular gap inside the FDTD region. Perfectly Matched Layer Boundary condition is applied on all sides. The source used is Total Field Scattered Field (TFSF), and the reflectivity monitor is placed outside the TFSF region behind the source.

In this section, we will show the following:

3.3.1 For polarisation vector along the Gap between the spheres

1) The fluidic interactions of liquid oligomers result in Ga nanodroplets of a radius of no more than 50nm. When two Ga nanospheres of the same radius (< 50nm) are embedded in PDMS (dielectric of refractive index 1.4), they exhibit gap plasmon resonance in the visible range if the polarisation vector is along the Gap between the spheres. It is the primary cause of resonance in this configuration.
2) The gap plasmon resonance blue shifts with the increase in the Gap (Figure 24a).
3) The resonances at shorter wavelengths correspond to collective dipole and Quadrupole oscillations of electrons in both spheres.
4) With the decrease in radii, the plasmon resonance blue shifts (Figure 25b).

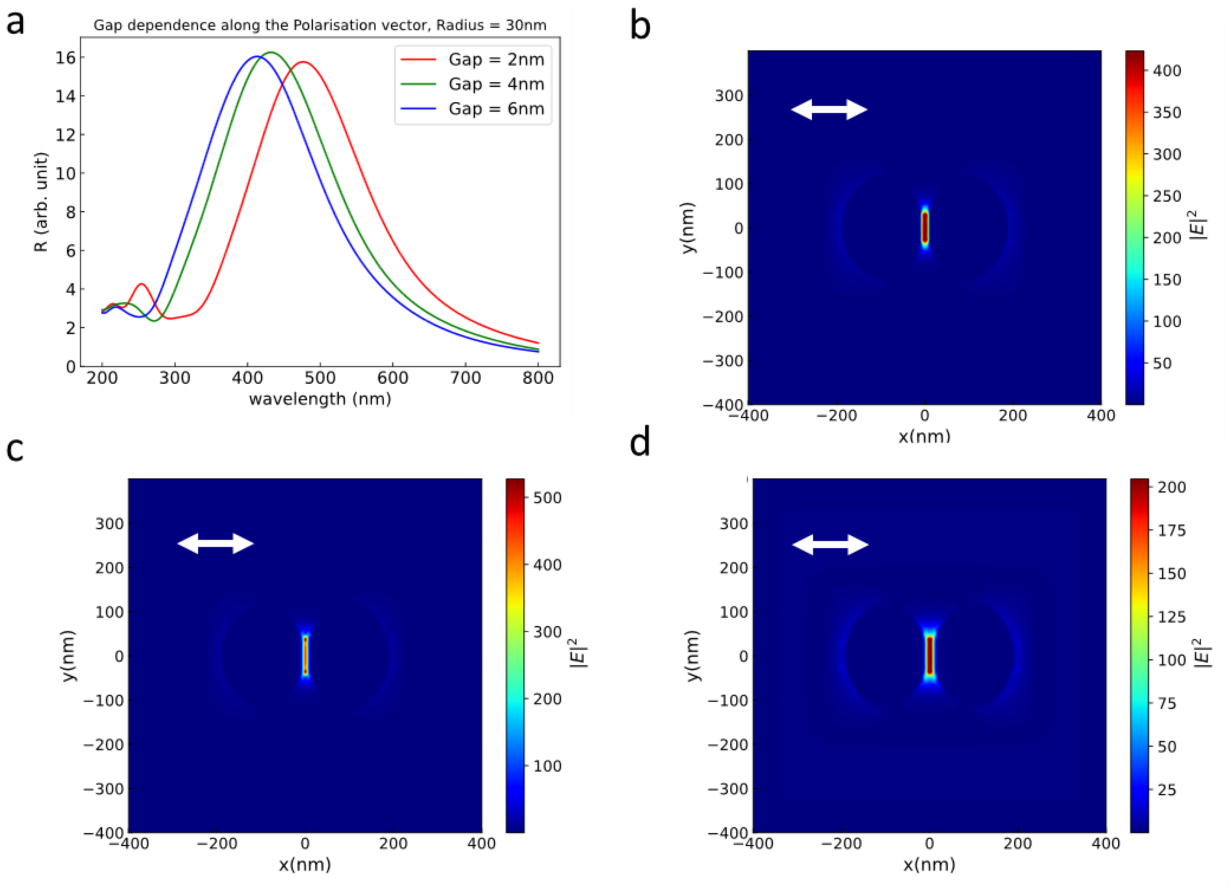

Figure 34: Gap plasmons variation with dielectric Gap. a) Reflectivity plot obtained from FDTD simulation. It indicates the blue-shift of the spectrum with the increase in the Gap between the two spheres. The Gap plasmon resonance occurs in the visible region. b-d) The field intensity ($|E|^2$ with units (V^2/m^2)) plots plotted at the gap plasmon resonance for a gap of 2, 4 and 6 nm respectively.

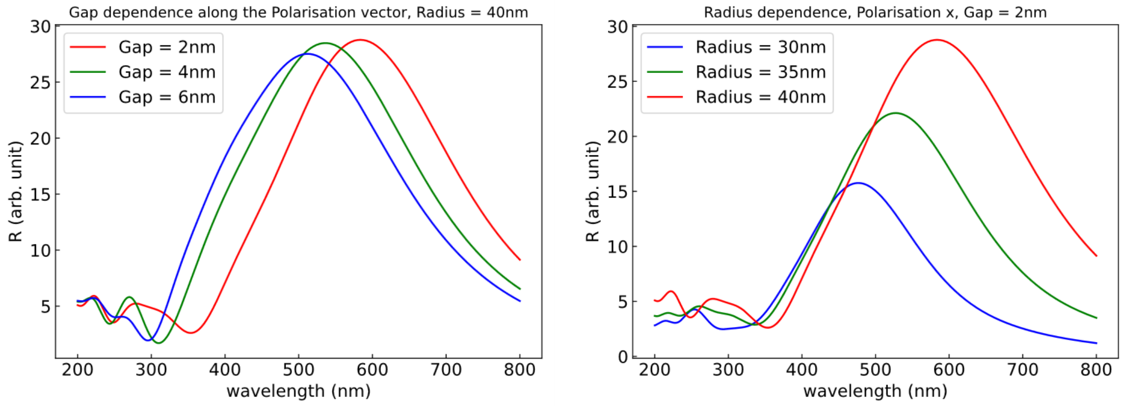

Figure 35: Reflectivity plot from two spheres at the different gap (left) and different radii(right).

3.3.2 For polarisation vector perpendicular to the gap between the spheres

1) The polarization vector perpendicular to the gap shows negligible interaction between the fields scattered by two nanospheres. This phenomenon is effectively a single-particle effect and can be approximated as two independent single-particle scattering events co-occurring, as indicated by the simulation results in Figure 26.

2) Since these scatterings are independent and the Ga droplets are spherical, we can apply Mie theory to understand the spectral features in the UV region.
3) The primary cause of resonance is the collective oscillation of electrons in the dipole mode of the spheres.
4) The reflectivity spectra of these systems of spheres are independent of the gap between them.

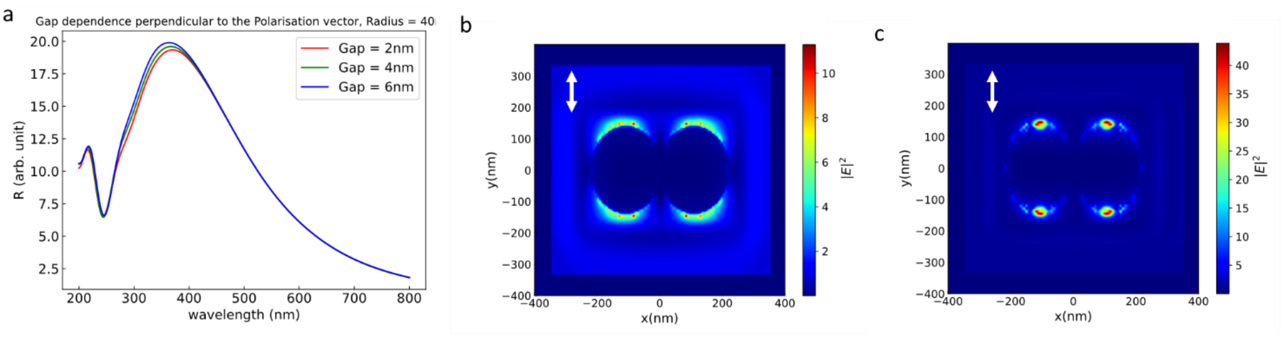

Figure 36: Independent scattering by the two nanospheres in the case where polarisation is perpendicular to the direction of the gap. a) The reflectivity spectrum does not change by increasing the gap between the spheres. The major resonance is due to dipole resonance field distribution ($|E|^2$ with units (V^2/m^2)) at the UV region, as plotted in (b). The minor peak is due to quadrupole resonance field distribution ($|E|^2$ with units (V^2/m^2)) at the extreme UV region, as shown in (c).

The above simulation readily predicts the following spectral behavior observed experimentally on applying a uniaxial strain to the sample. We observe that the spectral features in the UV region do not change, and those in the visible and IR region get blue-shifted (Figure 26a).

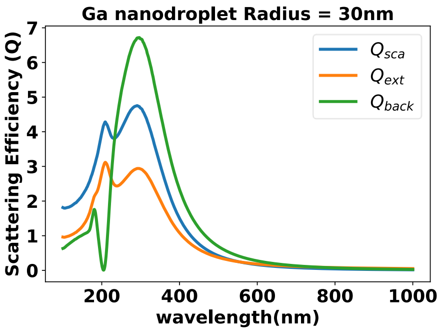

Figure 37: Efficiencies of Scattering, Extinction, and back-scattering of Ga sphere of 30 nm radius embedded in a medium of refractive index 1.4 as calculated from Mie Theory.

The UV spectral features can be attributed to the independent single-particle scattering of light by the individual Ga nanodroplets resulting from the interaction of those electric field components perpendicular to the gap between particles. As shown previously with the two-particle simulation, the spectral features due to single-particle scattering do not exhibit significant change. As shown above, in Figure 27, the scattering by the individual Ga spheres is limited to the UV region. On the other hand, the spectral features in the visible and IR region get blue-shifted, whose cause can be attributed to the increase in the inter-particle gap due to the uniaxial stretching (Figure 26a and 28).

In the following, we show the blueshift trend in the visible region and the null shift in the UV region of reflectivity spectra of different samples with respect to uniaxial stretching (Figure 28). It is

corroborated by multiple and distinct simulations exhibiting the aforementioned spectral trends on applying a uniaxial strain.

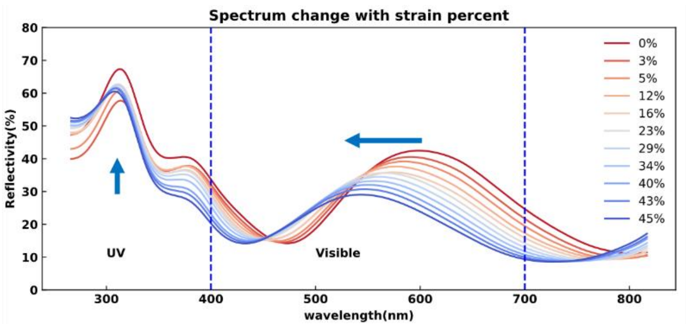

Figure 38: Experimentally obtained spectra of Ga deposited PDMS sample (Thickness: 100nm, Temperature: 35C, PDMS 10) at different strain percentages. The blue shift occurs in the visible and IR regions, whereas no such shift is observed in the UV region.

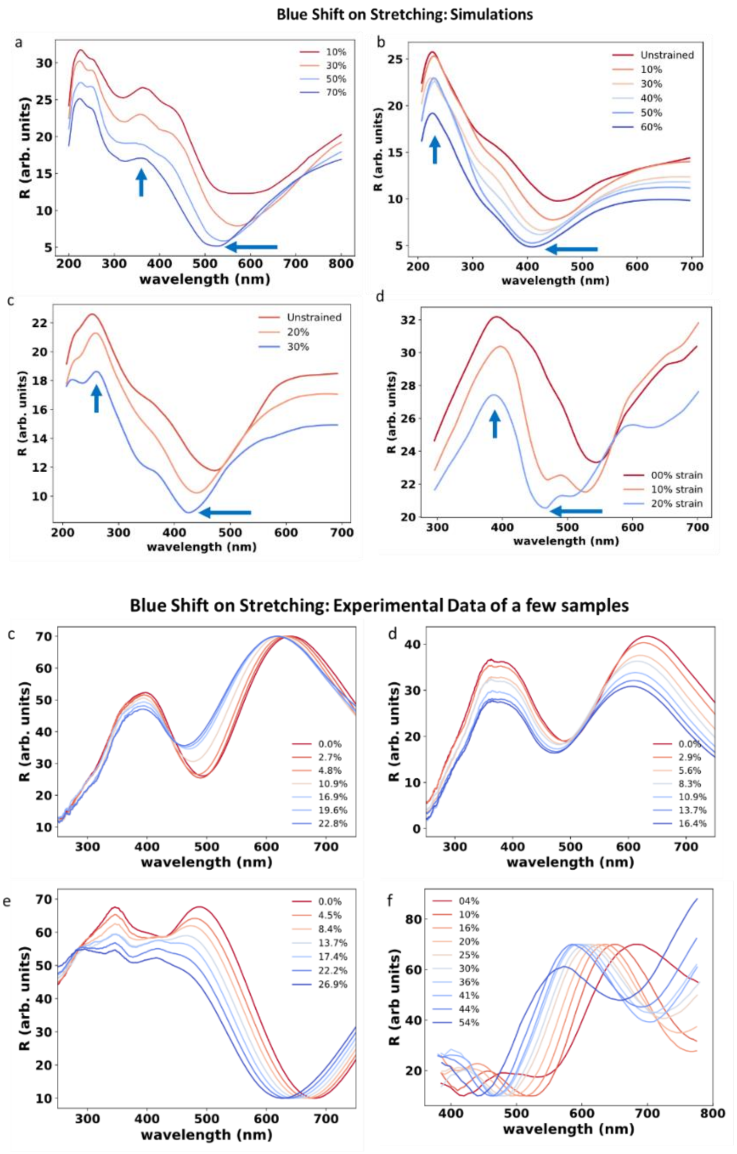

Figure 39: Blue shift in the visible region and null-shift in the UV region of simulated and experimental spectra of Ga deposited PDMS sample at different strain percentage. a, b, c, d, Reflectivity spectra obtained from four different simulated structures varying in number of layers, and size distributions. e, f, g, h, Experimentally obtained reflectivity spectra of four different fabricated samples.

3.4 Effect of native-oxide on Ga nanodroplets layer on its optical properties Ga-on-PDMS

3.4.1 Presence of native oxide on Ga nanodroplets

The deposition of Ga was started only after a vacuum of 9.9×10^{-6} milibar is reached, at which there is negligible chance of Ga oxide layer formation due to the unavailability of oxygen atoms. During the formation, the Gallium is in its elemental form. However, after taking the sample out from the evaporation chamber, atmospheric oxygen diffuse into the PDMS substrate and form the native oxide of Ga around the Gallium nanodroplets. The presence of native oxide is confirmed by X-ray photoelectron spectroscopy[1,8,9] as shown below.

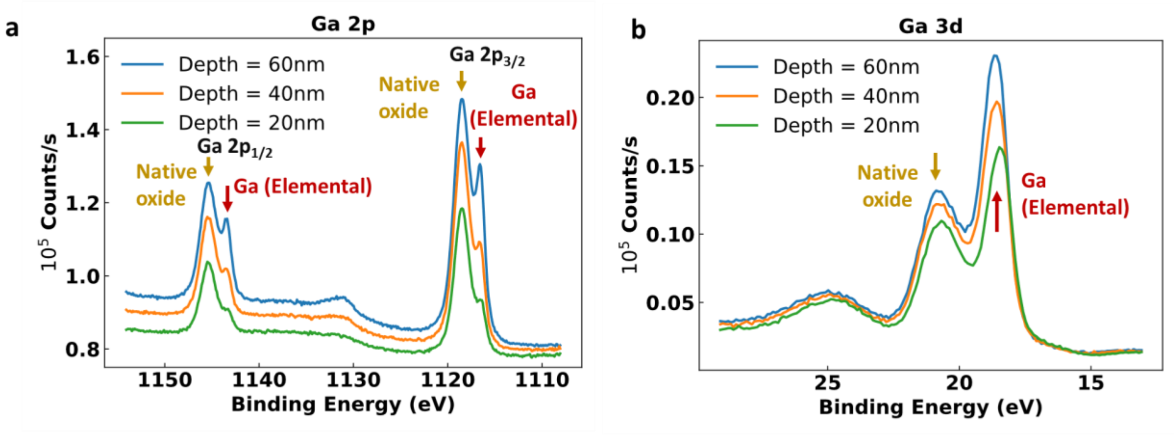

Figure 40 | X-ray photoelectron spectrographs of PDMS 10. High resolution scan of (a) Gallium 2p orbital and (b) Gallium 3d orbital shows the peak corresponding to Gallium native oxide.

3.4.2 Effect of native oxide on the optical properties

In the presence of Gallium Oxide layer, usually around 2-3nm thick [1], our simulations of optical properties show the following:

1. The chromaticity change due to the presence of the oxide layer is insignificant enough for visual discernment.
2. The trend of blue shift with respect to mechanical stretching.

The refractive index of Gallium oxide[10] is shown in the Figure 32a-b. The real part (Figure 32a) of the refractive index is taken from the reference [10], and the imaginary part (Figure 32b) is obtained by the Kramer-König constraint[11]. Figure 31c shows the reflectivity plot obtained in presence of different thicknesses of gallium native oxide layer. From the chromaticity values and the corresponding colors as shown in the inset of Figure 32d-e, we see no discernable change in color in absence (Figure 32d) or presence(Figure 32e) of the oxide layer. The blueshift of reflectivity spectra of increasing gap between the two Ga nanodroplets confirms that plamonic coupling between them still remains even in the presence of oxide layer(Figure 32f). Stretching simulation of multiparticle structure shows the optical trends observed in the absence of oxide layer (Figure 32g), that is, blue shift in the visible region and negligible shift in the UV region.

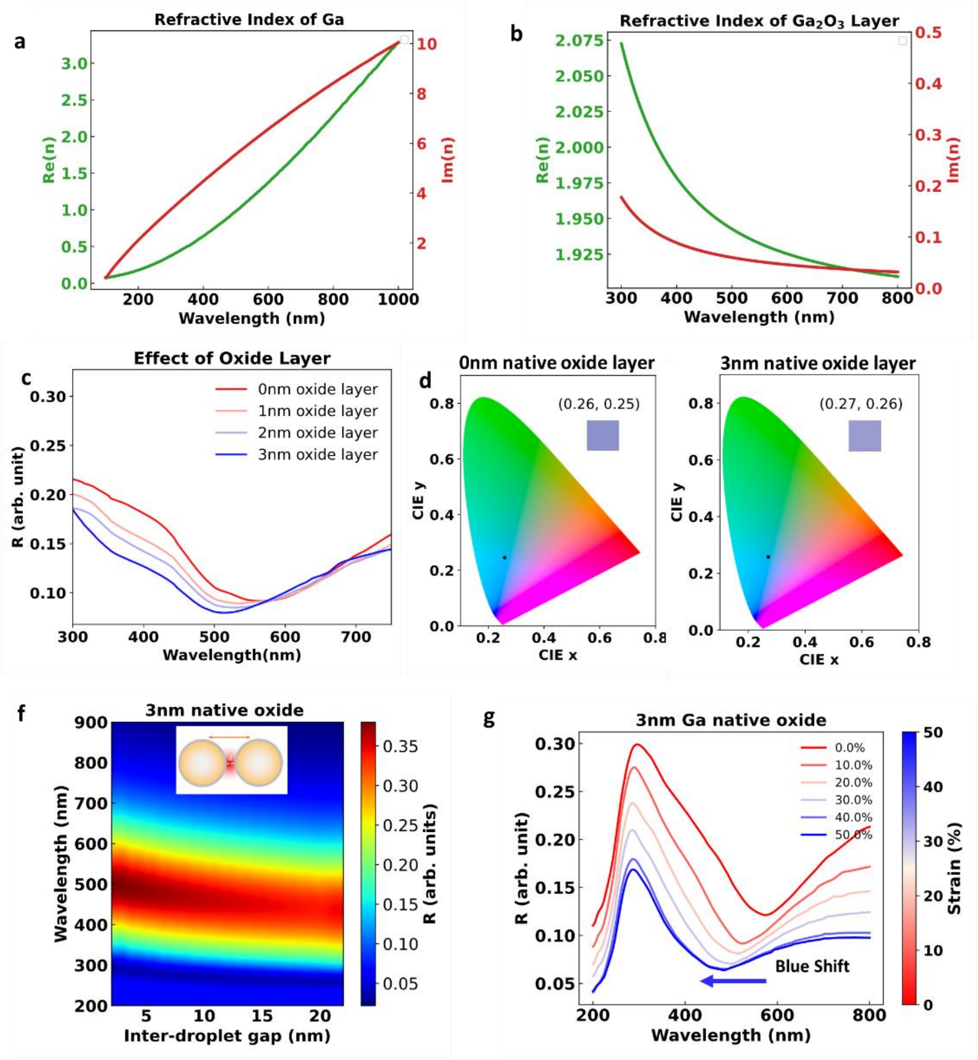

Figure 41: Effect of Oxide Layer, a, Refractive index of Gallium. b, Refractive index of Gallium oxide. c, Reflectivity of a PDMS 10, obtained from simulation, with different thickness of oxide layer around the Gallium droplets. d, Chromaticity of the sample without any oxide layer. e, chromaticity of the sample with 2nm oxide layer on the Gallium droplets. f, Blue shift of reflectivity by a dimer of Gallium droplet with 3nm Gallium oxide as the inter-droplet gap increases. g, Blue shift of the reflectivity of a 3-Layered multisphere structure, with each gallium droplet covered with 3nm Gallium oxide layer.

3.5 Structural and optical stability

During the process of Gallium nanodroplet formation Gallium remains in the liquid state. The high surface tension of Gallium keeps it spherical during the process of encapsulation as well as during the stretching of PDMS substrate in which the droplets are embedded in. The mechanical strain of the PDMS effectively results in the increment of inter-particle gap between the nanodroplets while the shape of the droplets remains fairly spherical thanks to the high surface tension of Gallium. Moreover, the native oxide layer around the Gallium nanodroplet also helps maintaining the spherical geometry.

Being embedded in the PDMS matrix, the relative position of Ga nanodroplets are fixed unless an external mechanical deformation is present. The Scanning Electron Microscope images before and after multiple cycles of periodic strain shows the structural reversibility of the device, as shown in the Figure 42. This reveals the structural stability, indicating that liquid metal leaking or liquids smearing

are highly improbable, still further corroborating the reversibility and stability of the optical performance of the device.

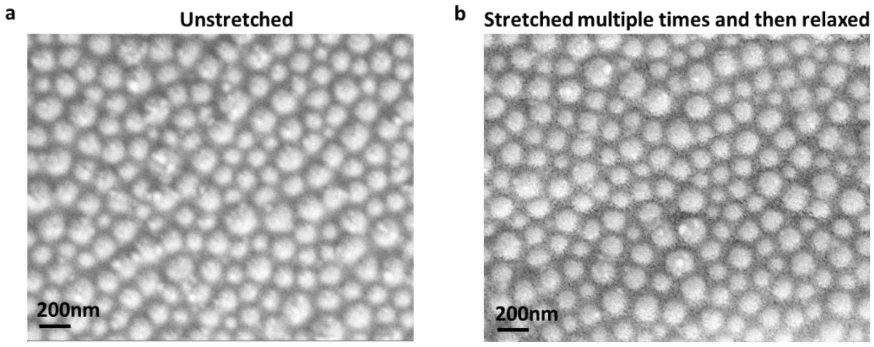

Figure 42: Top-view SEM images a, Sample stretched and relaxed 1000 times. b, As-fabricated sample, unstretched.

Furthermore the samples also show no change in color and spectrum with aging (Figure 43).

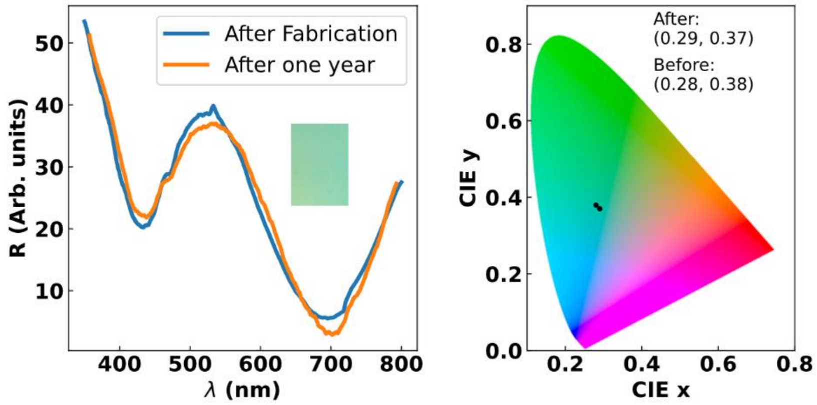

Figure 43: a, The spectra of the unencapsulated sample after fabrication and one year after fabrication. The inset is the optical image of the sample. b, The chromaticity of the sample remains same up to visual discernment.

To enhance the stability we further encapsulate the chromogenic structure with an additional PDMS superstrate layer. To address the reliability and stability of our device, we performed a series of experiments, as elaborated below:

Reliability and performance enhancement depend on the fact that our fabricated samples should show repeatable results in terms of resonance position as well as color (CIE) when subjected to (a) multiple cycles of strains, (b) change in temperature, (c) washability and or exposure to solvents and (d) physical contact.

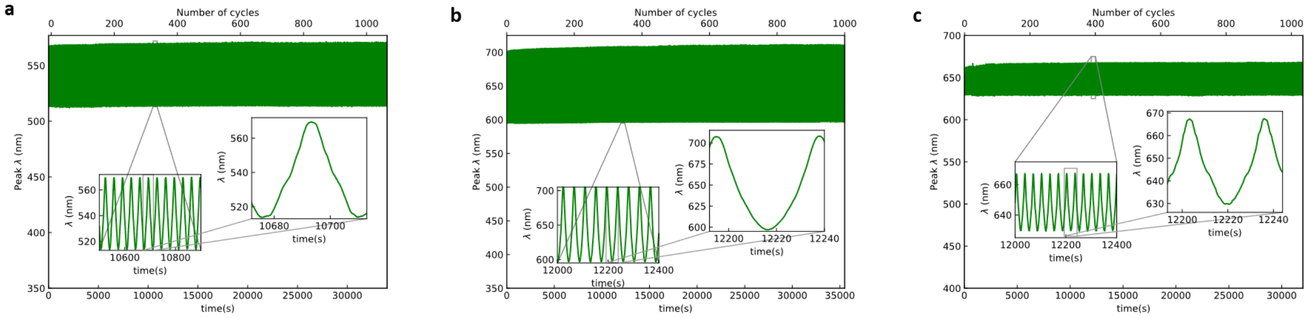

Figure 44: Cyclic test to check the optical stability of the samples. a, b, and c depict the peak position of the reflectivity spectrum as a function of time for different samples.

(a) **Effect of multiple strain cycle**:

The structural stability is results in high reversibility of the mechanochromaticity as shown in Figure 44, for three different samples.

Furthermore, we have done periodic cyclic tests up to more than 80000 cycles (Table 2 and Figure 45), surpassing any other values reported in the literature to the best of our knowledge, showing an improvement of an order of magnitude greater than the previous experiments, which were shown to be stable for 10000 cycles (Table 2).

Sl. No.	Reference	No. of cycles	Strain type	Spectra
a.	[12]	10000	Tensile	Reflection
b.	[13]	1000	Bending	Transmission
c.	[12]	2000	Tensile	Reflection
d.	[14]	20	Tensile	Reflection
e.	[15]	100	Tensile	Reflection
f.	[16]	Not Performed	Tensile	Absorption
g.	[17]	Not Performed	Tensile	Extinction
8	[18]	Not Performed	Tensile	Reflection
**9**	**Our Work**	**80000**	**Tensile**	**Reflection**

Table 2| Performance of our work as compared to others in the literature, showing a robustness of at least 80000 cycles.

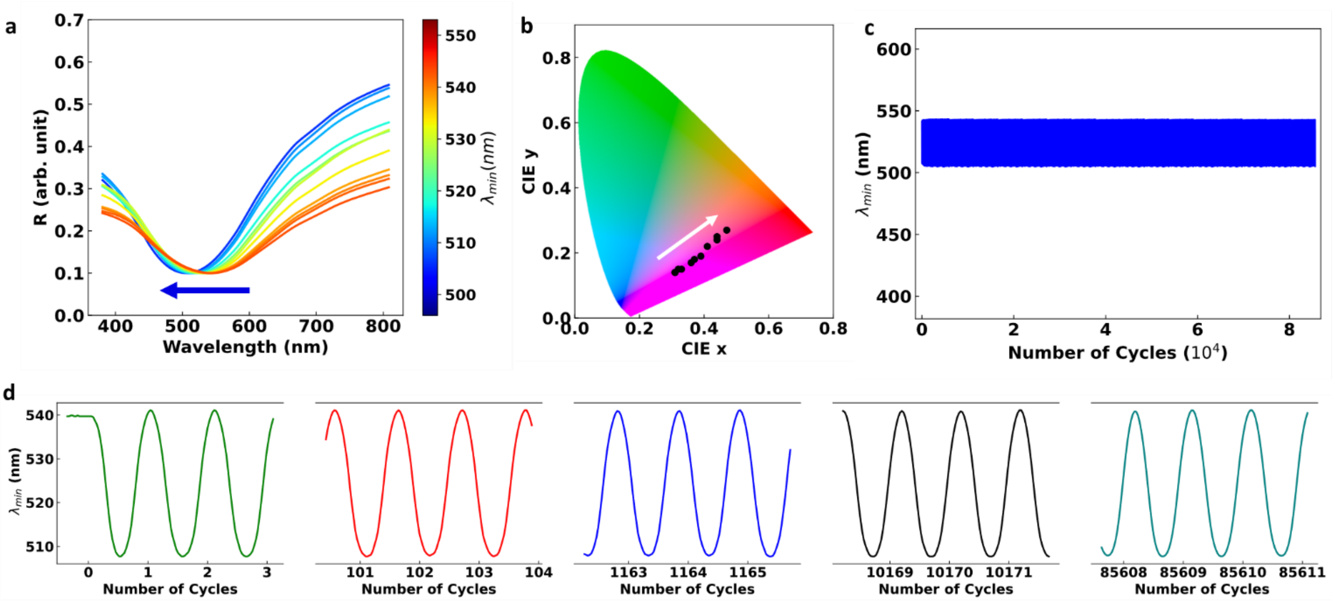

Figure 45: a, Reflectivity data during stretching. The direction of the arrow shows the direction of spectral shift with increasing strain. b, The shift chromaticity coordinates with increasing strain. c, Cyclic test for atleast 8x10⁴ cycles depicting the λ_{min} (wavelength at which the minimum of reflectivity sspectrum occurs). d, λ_{min} vs number of cycles near 1 (green), 10²(red), 10³(blue), 10⁴ (black), and 8x10⁴ (teal) cycles.

The maximum strain we have shown is surpassed to 92% (see Figure 46), only limited by the mechanical properties of PDMS. There is a degree of variability in the elastic properties of PDMS based on different PDMS ratios, as shown in Figure 2. Depending on the PDMS, one can achieve a strain of more than 100% as well.

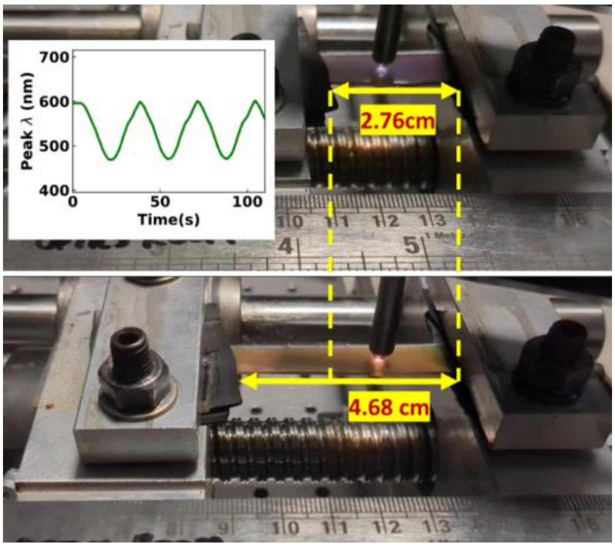

Figure 46: The PDMS samples can stretch to at least 92 %.

In real world-applications, we encapsulate the fabricated chromogenic structure with a superstrate layer of PDMS and thermally cured it to protect it from direct physical contact. Encapsulation with a superstrate PDMS layer maintains the stability of the chromogenic structures even with toluene treatment. We also observe that unencapsulated samples are also stable with respect to (a) mechanical strain (b) aging as well as (c) temperature. Below, we detail the experiments on the stability. Points 1-4 show the robustness and stability against:

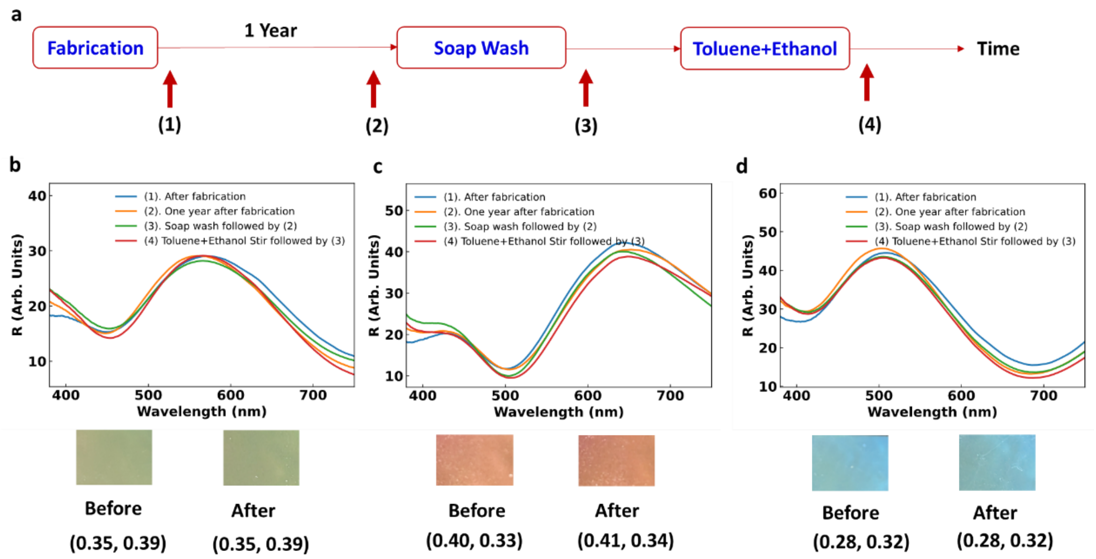

Figure 47: Stability against aging and washing. (a) The timeline of the sample and reflectivity measurements. The numbers in bracted under the upward arrows indicate the event of reflectivity measurement, shown in the legend of the following plots. (b), (c), (d). The reflectivity plot of the samples, (1)after the fabrication, (2) more than a year after the fabrication, (3)after washing with soap solution, (4) after treatment with toluene and ethanol. The color palettes shown are the camera images of the sample taken before the Soap wash (left) and after the toluene+Ethanol treatment (right). The chromaticity coordinates (CIE x, CIE y) of each of the samples are shown below their respective images.

1. **Stability with age**: Samples fabricated one year before had the reflectivity spectra intact and appeared the same in color, owing to the structure preservation, thanks to the additional protective PDMS layer (Figure 47).
2. **Stability and durability with respect to solvents (toluene and ethanol):** 6 hours of magnetic stirring of the sample in toluene followed by 3 hours in ethanol resulted in mechanical deformation and swelling. As the solvent evaporates, the shape and size of PDMS, as well as the structural color, reverts again (Figure 47).
3. **Stability and durability with respect to washing (wearable device)**: We stirred the samples in a commercial soap solution for 6 hours and then dried the sample before taking reflectivity measurements (Figure 47, video SI video 11).
4. **Stability with respect to temperature:**
    i. We have performed a temperature dependence study up to 134°C where the resonance position is shown in Figure 48. The spectrum remains essentially the same with $\Delta\lambda < 2.5nm$.

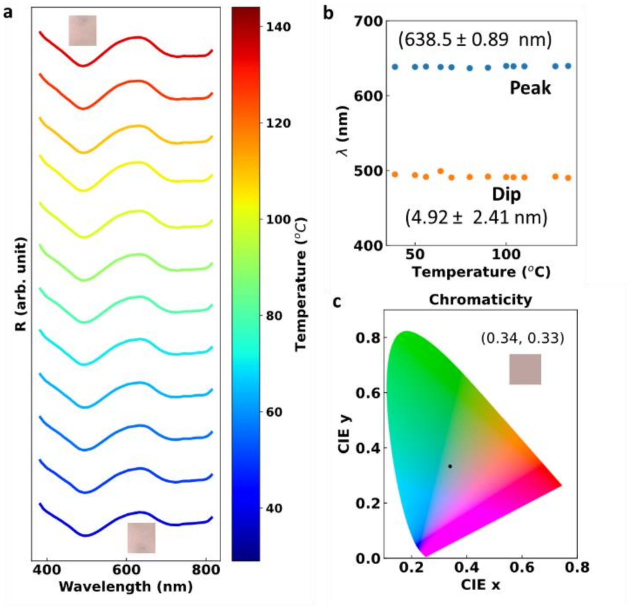

Figure 48: Stability against temperature. (a) Reflectivity plot of the sample with varying temperature. The inset shows an optical image of the sample. (b) Peak (638.5 ± 0.89 nm) and dip (4.92 ± 2.41 nm) of the reflectivity spectrum with respect to temperature. (c) Chromaticity of the sample for these reflectivity spectra.

ii. In addition, we exposed two of the samples to 180°C on a hot plate to check if the sample performs consistently over time. As shown in Figure 49, the reflectivity spectra remain stable for more than 12 hours at 180°C. Moreover, the extrema of the reflectivity spectra have a standard deviation of less than 1nm.

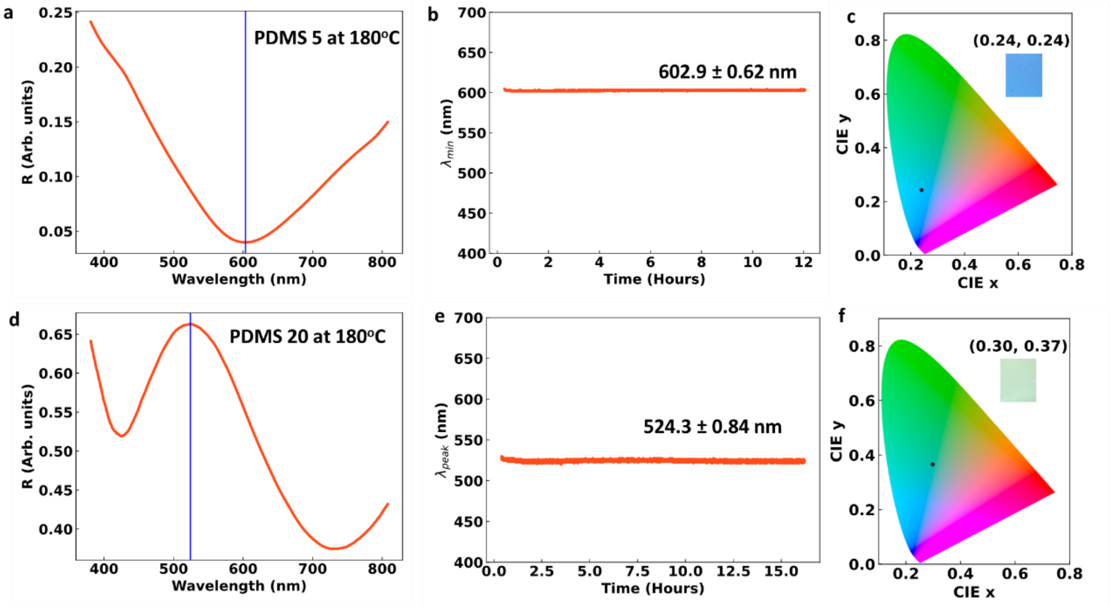

Figure 49: Stability of un-encapsulated sample against high temperature for hours. a, Reflectivity of PDMS 5 at 180°C. The blue vertical line represents the wavelength location of reflectivity extremum b, The extremum wavelength of PDMS 5 (shown in blue in Figure a) as a function of time. The extremum wavelength value deviates by less than 0.62nm from its mean. c, CIE coordinates of the sample. The inset shows the image of the sample PDMS 5. d, Reflectivity of PDMS 20 at 180°C. e, The extremum wavelength of PDMS 20 (shown in blue in Figure c) as a function of time. The peak wavelength deviates by less than 0.85nm from its mean. f, CIE coordinates of the sample. The inset shows the image of the sample PDMS 20.

5. **Stability of stretched sample**:

   The reflectivity spectrum of 51% stretched Ga-deposited PDMS 10 (see Figure 50a) shows a consistency in the spectral features up to a standard deviation of 1.21 nm. This implies durability of the samples and stability against continuous strain for at least 30 hours.

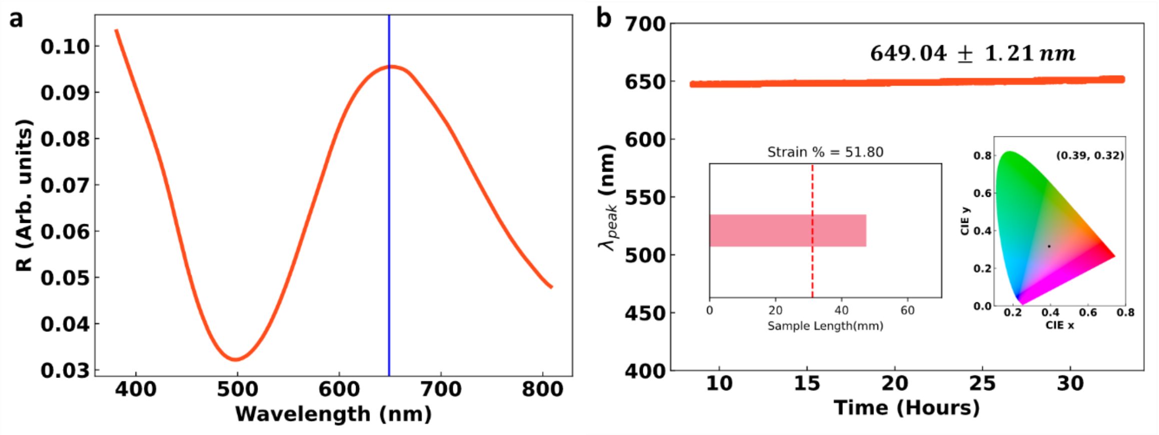

Figure 50: Stability of stretched sample. a Reflectivity at 50% stretch. The blue vertical line represents the wavelength location of reflectivity extremum b, and the extremum wavelength of PDMS 10 (shown in blue in Figure a) as a function of time. The extremum wavelength value deviates by less than 2nm from its mean. (Inset, left) The strain of the sample during the spectrum acquisition was 51.8%. (Inset, right) The chromaticity coordinates of the stretched sample.

6. **Stability against physical contact:** In the submitted SI videos, we expose the samples to harsh physical contact, like poking with a tweezer (Supplementary Video 7), helical twisting (Supplementary Video 8), and tying a knot (Supplementary Video 9), and all showing stability against physical contact.
   - **Supplementary Video 7:** Point Sensing: In this procedure, we employ the tip of a tweezer to exert a localized force on a small region, typically of the order of $1mm^2$.
   - **Supplementary Video 8**: Helical twist: Here, we twist the fabricated sample along its longitudinal length, exposing the sample to physical contact with itself.
   - **Supplementary Video 9**: Knot: Here, we loop the fabricated sample, in which the samples get rubbed against each other while tightening the knot.
   - **Supplementary Video 11**: Stability in harsh environmental conditions: Encapsulation with a PDMS superstrate enables preservation of Ga nanostructure even in the case of toluene treatment, as shown in Figure 47.

4. Color characterization and other applications

4.1 CIE coordinates from Reflectivity Spectra

The codes for determining the CIE coordinates from reflectivity spectra can be found in https://github.com/RenuRamanSahu/ColorConversionCodes_ReflectivityToCIE. The following steps are followed to obtain the CIE x and y coordinates for a given reflectivity spectrum [19].

1. A reflectivity spectrum obtained in arbitrary units and normalized with a known reference (such as a silver mirror) is needed as an input.
2. We have the illuminant D65 spectrum as the source of light that determines illumination. The response of human eyes to different wavelengths is encoded in terms of three functions called the color-matching functions, denoted by $\bar{x}, \bar{y}$ and $\bar{z}$.

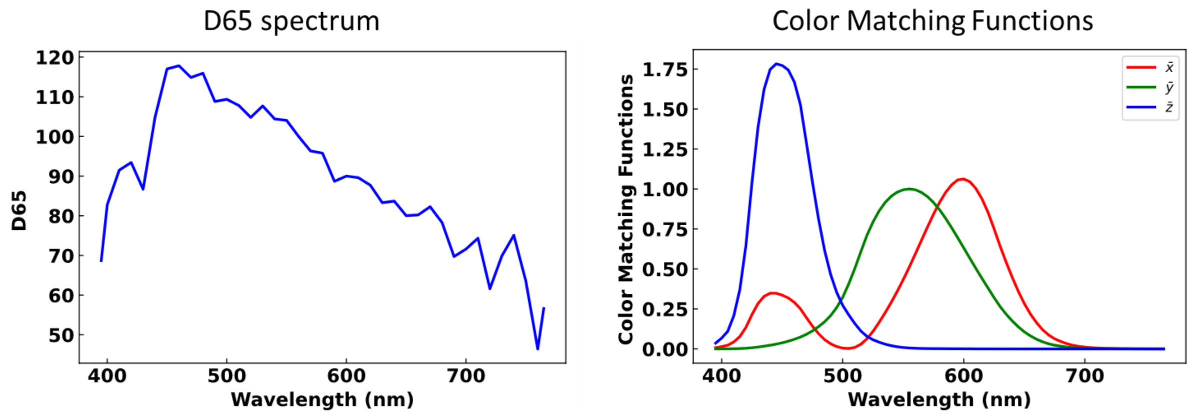

Figure 51: Data that encapsulates the illuminant and response of eyes to a spectrum. a, Spectrum of illuminant D65. b, Color matching functions.

3. The tristimulus for white light (D65) is obtained by,

$$X_{D65} = \frac{\sum \bar{x}.D65}{\sum \bar{y}.D65} = 0.950$$

$$Y_{D65} = \frac{\sum \bar{y}.D65}{\sum \bar{y}.D65} = 1.000$$

$$Z_{D65} = \frac{\sum \bar{z}.D65}{\sum \bar{y}.D65} = 1.088$$

where the dot (.) operation gives another array with each element as obtained from the elementwise multiplication of the two arrays. The expression $\sum \bar{x}.D65$ refers to the sum of the elements in the array obtained by elementwise multiplication of $\bar{x}$ and $D65$.

4. The white point is given by,

$$x_{D65} = \frac{X_{D65}}{X_{D65} + Y_{D65} + Z_{D65}} = 0.313$$

$$y_{D65} = \frac{Y_{D65}}{X_{D65} + Y_{D65} + Z_{D65}} = 0.329$$

5. Then we calculate S by elementwise multiplication of the reflectivity spectrum and $D65$,

$$S = R \cdot D65$$

6. The tristimulus values are then obtained for S,

$$X = \frac{\sum \bar{x}.S}{\sum \bar{y}.D65}$$

$$Y = \frac{\sum \bar{y}.S}{\sum \bar{y}.D65}$$

$$Z = \frac{\sum \bar{z}.S}{\sum \bar{y}.D65}$$

7. The CIE x and y coordinates can be obtained from the tristimuli values as follows,

$$x = \frac{X}{X + Y + Z}$$

$$y = \frac{Y}{X + Y + Z}$$

The CIE chromaticity coordinates are given by x and y, while the value of Y denotes the object's brightness from which the reflectivity spectrum is measured.

RGB and Hue from CIE xyY coordinates

For a given color specified by CIE x, y and Y, one can obtain the RGB values by following steps:

1. We obtain z by,

$$z = 1 - x - y$$

2. The rest of the tristimuli values are determined as,

$$X = \frac{xY}{y}$$

$$Z = \frac{zY}{y}$$

3. We obtain $R', G', and\ B'$ values from the following matrix operation,

$$\begin{pmatrix} R' \\ G' \\ B' \end{pmatrix} = \begin{pmatrix} 3.2404542 & -1.5371385 & -0.4985314 \\ -0.9692660 & 1.8760108 & 0.0415560 \\ 0.0556434 & -0.2040259 & 1.0572252 \end{pmatrix} \begin{pmatrix} X \\ Y \\ Z \end{pmatrix}$$

4. Let S' and S belong to the corresponding values in (R', G', B') and (R, G, B) respectively.
5. The values of R, G and B are obtained by the following conditional operation

If $S' < 0.0031308$,

$$S = 12.92\, S'$$

Else,

$$S = 1.055 \quad S'^{(1.0/2.4)} - 0.055$$

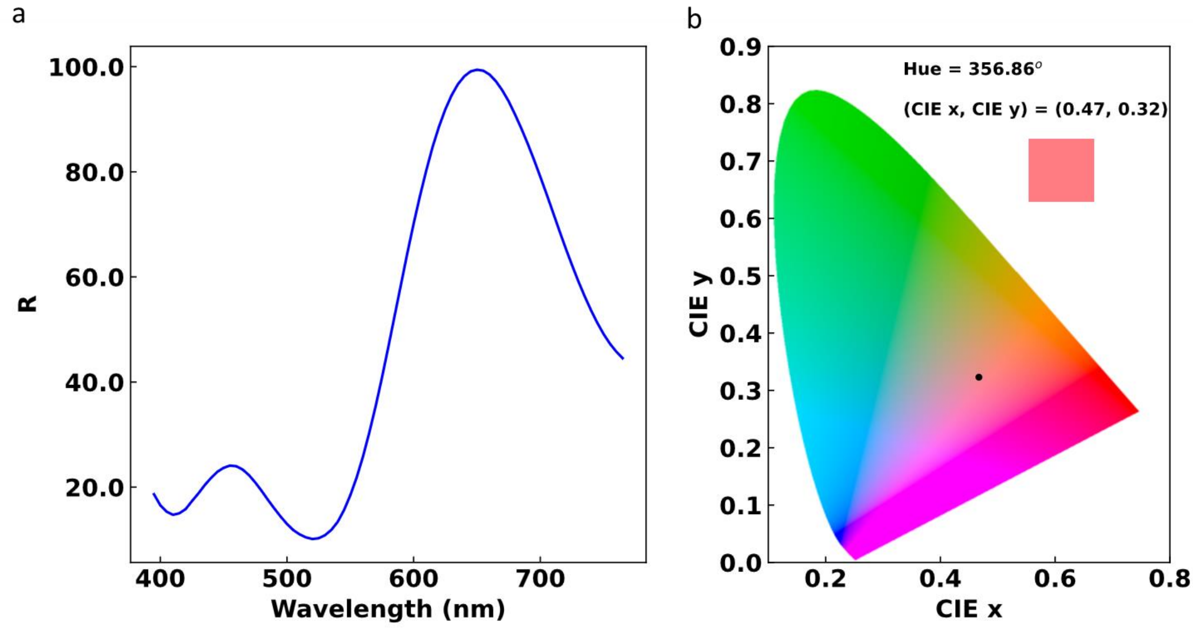

Figure 52: Reflectivity plot and corresponding color coordinates. a, Reflectivity spectrum obtained from spectrophotometer and normalized with respect to the reference. b, The CIE x and y coordinates and hue corresponding to the reflectivity spectrum.

6. Having obtained the values of R, G, B, we obtain hue by the following procedure:
   a. We evaluate $C_{max}, C_{min},$ and Δ as,

$$C_{max} = max(R, G, B)$$

$$C_{min} = min(R, G, B)$$

$$\Delta = C_{max} - C_{min}$$

   b. If $\Delta = 0$, the value of the hue is $H = 0$
   c. If $C_{max} = R$, the value of the hue is $H = 60 \times \left(\frac{G-B}{\Delta}\right)$
   d. If $C_{max} = G$, the value of the hue is $H = 60 \times \left(\frac{B-R}{\Delta} + 2\right)$
   e. If $C_{max} = B$, the value of the hue is $H = 60 \times \left(\frac{R-G}{\Delta} + 4\right)$

7. The hue range obtained from the above procedure is from 0 to 360 degrees. In cases where the hue values are reported more than 360 degrees in the main text, the hue is to be wrapped to the range mentioned above by a modular division by 360. For example, hue value of 400 degrees is equivalent to 40 degrees (= 400 mod 360).

4.2 Determination of Curvature from the image.

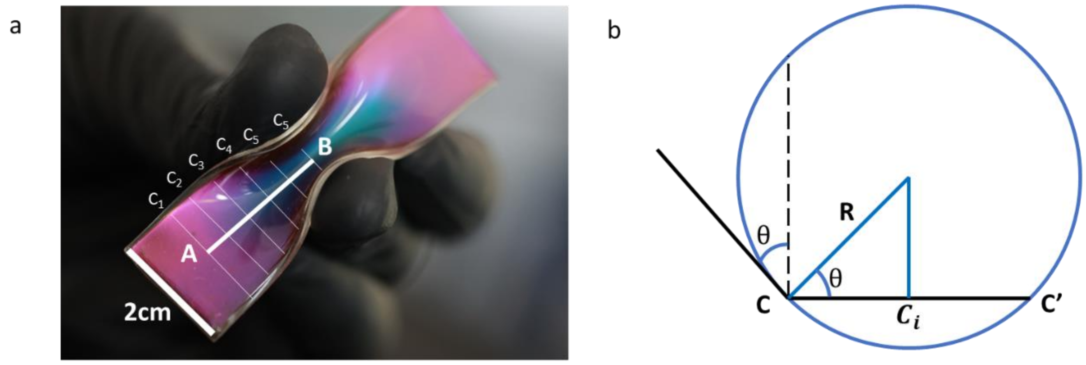

Figure 53: Determination of the radius of curvature from the image along the line AB. a, Optical image of the sample showing a color change with varying curvature. b, The geometrical construction used to determine the radius of curvature for chord length C_i.

To obtain the curvature from point A to B along the line AB we assume that linear variation of the edge angle from 0 to 90 degrees. The chord lengths C_i (= CC') are determined to vary from 2cm to 0.47 cm at intermediate intervals along AB. The geometry (Figure 53b) is used to determine the curvature using the above data.

The corresponding hue can be computed by the abovementioned method after determining the RGB values using image processing software (ImageJ FIJI).

$$R = \frac{C_i}{2\, cos\, \theta}$$

5. Novelty of the work

5.1 Novelty in the material processing: Active Substrate

While there are several methods to fabricate the structural colors like spin coating[20–25], 3D printing[26–36], rubbing[37], etc., most of them are fabricated on rigid substrates, particularly for display applications. Incorporating mechanochromic properties requires the usage and smart design of structures on flexible and deformable materials. The general fabrication strategy of mechanochromic structures is to employ a flexible substrate or polymer, in which a pre-synthesized active material is incorporated. In these cases, the role played by the substrate is to support, or embed the active media, and incorporate the functionality of mechanical flexibility. Herein, we employ PDMS as the substrate. Although previously reported mechanochromic structures have employed PDMS [38–42], their emphasis has been on the efficient synthesis and smart design of the active media that incorporates color change (Table 3). Herein, we exploit the fluidic nature of the oligomers infused in the PDMS substrate for controlling the structure. In this work, we demonstrate, for the first time, the active participation of the substrate in determining the nanostructure, allowing the simultaneous fabrication of multiple structural colors.

Sl No.	Processing	Substrate / Elastomeric matrix	Material	Optical Mechanism	Mechanochromicity / Mechanism	Cyclic stability	Reference
1	Lipmann Photography technique to print bragg reflectors	Elastomeric Photopolymer C-RT20	1D periodic refractive index structure (Distributed Bragg Reflectors)	1D band gap	Yes, Photonic band-gap tuning	atleast 1000 cycles	[1]
2	Metal-Insulator-Metal structure	Parylene	Silver, SiO_2	Fabry-perot interference	Yes, interference	1000 cycles	[2]
3	Sequential mixing of water, Hydroxypropyl cellulose, carbon black, gelatin to form liquid crystals	Gelatin	Hydroxypropyl cellulose, gelatin	Liquid-crystal	Yes, tuning liquid crystal orientation	Not-shown	[3]
4	A liquid precursor of Stober-silica particles in ethanol and di(ethylene glycol) ethyl ether acrylate (DEGEEA)	DEGEEA	Silica microspheres	Photonic crystal in matrix of DEGEEA	Yes, Photonic band-gap tuning	Not-shown	[43]
5	Silica dispersed in photocurable PEGPEA polymer matrix, casting the resultant colloid between glass-slides to form mechanochromic film	Photocured - PEGPEA	Non-closed packed silica microspheres	3D photonic crystal	Yes, Photonic band-gap tuning	atleast 20 cycles	[5]
6	poly(butyl arylate) (PBA) spheres bearing 2-ethylhexyl acrylate which helps to bind the PBA microspheres with Polyacrylamide	UV-cured polyacrylamide	poly(butyl acrylate) (PBA) spheres, 2-ethylhexyl acrylate(EHA), Polyacrylamide(PAM)	3D photonic crystal	Yes, Photonic band-gap tuning	Not-shown	[6]
7	Triblock copolymer, linear polymethyl PMMA chains and a bottlebrush-like PDMS segment	PDMS	Microphase separated blocks of copolymer	Scattering	Yes	Not-shown	[7]

**8**	Gold nanoparticle preparation, Pickering Emulsification of Au on polyethoxyorthosilicate (PEOS), coating substrate with the emulsion	PVA	Pickering emulsion of Gold NP on Silica microspheres	Plasmonic-based scattering	Yes, Plasmonic Shift	Not-shown	[8]
**9**	Preparation of Gold Nanoparticles (NP), Treatment of PDMS 10 with plasma for surface functionalisation, Growth of NP by dipping PDMS in Au solution	PDMS	Gold Nanoparticles	Gap-Plasmons	Yes, Plasmonic shift in absorption spectra	Not-shown	[9]
**10**	Preparation of Gold Nanoparticles, Transfer onto PDMS by horizontal lifting	PDMS	Gold Nanoparticles	Gap-Plasmons	Yes, Plasmonic Shift	Not-shown	[10]
**11**	Preparation of Gold Nanoparticles, Transfer onto PDMS by chemical absorption	PDMS	Gold Nanoparticles	Gap-Plasmons	Yes, Plasmonic Shift	Not-shown	[11]
**12**	PreparationSilver capped Polystyrene spheres, transfer onto PDMS	PDMS	Silver-capped Polystyrene spherers	Gap-Plasmons	Yes, Plasmonic Shift	Not-shown	[12]
**13**	This paper: Thermal Evaporation of Ga onto PDMS to form Non-coalescent Ga nanospheres due to liquid-liquid interaction	PDMS	Ga	Gap-Plasmons	Yes, Plasmonic Shift	atleast 1000 cycles	Novelty: (Active participation of substrate): Exploiting the fluidic property of oligomers in PDMS for formation of the chromogenic structure

Table 3 | Comparison of our work with existing mechanochromic structural colors.

5.2 Novelty in the material processing: Tuneable Plasmon-coupled Ga nanostructure

Gallium has been established as a plasmonic material[44–48], at par with gold and silver, the usually employed plasmonic nanoparticles. Overcoming the high surface energy of Gallium poses a challenge to fabricating monodispersed Ga nanodroplets on a thin film by physical methods, therefore to the best of our knowledge, no such work has yet been reported. There are attempts to reach the sub-100nm size regime, but it has been limited mostly to chemical methods, and the resultant nanoparticles formed are in a liquid medium[49–52]. On the other hand, the thin film-based plasmonic Ga nanostructures are on hard substrates like Si[45,47], anodized Aluminium,[46] SiO_2[48] etc. (Table 4). Herein, we have not only exploited the fluidic property of Ga for nanodroplet formation on an elastomeric substrate, a feat achieved for the first time by a physical method, but also obtained the plasmonic-coupling between the closely-spaced nanodroplets in a single step, which requires multiple fabrication steps in previously reported plasmon-coupled structures. Previously reported Ga-on-PDMS structures have been limited to flexible electronics, and microfluidics applications, whereas our research has opened doors for flexible-photonic applications of Gallium nanostructures. From an application perspective, it allows for structural color fabrication and paves the way for tuneable plasmonic research.

Sl No.	Processing	Type	Flexible Film	Liquid Metal	Shape/Feature size	Reference
1	Gallium Effusion Cell	Thin film, substrate: SiN	No	Ga	Spherical caps, 14nm - 200nm diameter	44
2	Thermal evaporation	Thin film, substrate: templated Anodised Aluminium	No	Ga	Spherical caps, 100nm diameter	46
3	Molecular Beam Epitaxy	Thin film: substrate: Si(111)	No	Ga	Thin film, thickness-23nm	47
4	Molecular Beam Epitaxy	SiO_2 on Si	No	Ga	Spherical caps, 40nm-100nm diameter	48
5	Thermal evaporation	Si(100)	No	Ga	Spherical caps, 27nm-400nm diameter	45
5	Thermal decomposition of Ga alkylamides	Liquid medium, 1-octadecene as solvent	No	Ga	Spheres, 12nm-46 nm diameter	51
6	Probe Sonication	Liquid medium, Suspension in Ethanol	No	EGaIn	Spheres, 50nm-150nm diameter	52
7	Redox reaction between solid metal Zn NPs and $GaCl_3$ solution	Liquid medium: Solvents	No	Ga	Spheres, 118nm diameter	50
8	Hot injection of $GaCl_3$	Liquid medium: phosphate-buffered saline	No	Ga	Spheres 10-20nm diameter	49
	This work:					
9	Thermal evaporation	Thin film: PDMS substrate, flexible	Yes	Ga	Controlled by substrate property, sub-100nm can be obtained	**Novelty: Application of plasmonic coupling in Ga nanoparticles for mechanochromicity.**

Table 4| Comparison of our work with existing Ga-based nanostructures.

The salient novelty accompanying the fabrication process as well as the properties of the sample, summarised from above discussion are as follows:

1) **An advancement in processing:** We are now harnessing the principles of fluidic interactions to create nanostructures and produce structural colors, marking a departure from the previous reliance on intricate chemical and lithographic methods.

2) **Single-step fabrication of multiple chromogenic structures:** The only processing step entails the thermal evaporation of Gallium to PDMS substrates, thus making the whole process a single step, scalable and cost-effective.

3) **Static relative positions of Gallium nanodroplets for a given mechanical configuration:** Unless an external deformation is applied, the Ga nanodroplets do not change their relative position with respect to each other. Despite multiple cycles of periodic strain, one retains the initial color, indicating the robustness of the sample towards structural degradation.

4) **Non material-intensivity:** Only the substrate and the material to be deposited take part in the fabrication, whereas other techniques require multiple chemical steps involving solvent-based precursors before the structural color gets fabricated.

5) **A physical method to leverage high-surface energy of Gallium:** Fabrication of nano-sized Ga particles on thin-film by a physical method demonstrating a Reversible tunability of the inter-droplet gaps via external mechanical deformation.

6) **Advancements in Optical Materials:** Liquid plasmonic materials, such as gallium (and related alloys), can now be utilized in the visible spectrum for the first time. Precisely controlled and adjustable gaps between separate nanodroplets are employed to generate gap-plasmons, enabling dynamic control over the spectral characteristics across the entire visible spectrum.

Data Availability:

The data for figures of the manuscripts are available in

https://github.com/RenuRamanSahu/Manuscript1_Data

Code Availability:

1. Code for getting CIE from reflectivity data:
RenuRamanSahu/ColorConversionCodes_ReflectivityToCIE: To obtain CIE color coordinates from reflectivity spectra of a sample. (github.com)

   https://github.com/RenuRamanSahu/ColorConversionCodes_ReflectivityToCIE

2. Code for real-time acquisition of reflectivity spectra:
RenuRamanSahu/RealTimeSpectralAcquisition: Take data from Ocean Optics Spectrometer and show the reflectivity, CIE color coordinates and the peak wavelength vs. time. (github.com)

   https://github.com/RenuRamanSahu/RealTimeSpectralAcquisition

6. References

1. Okatenko, V. *et al.* The Native Oxide Skin of Liquid Metal Ga Nanoparticles Prevents Their Rapid Coalescence during Electrocatalysis. *J Am Chem Soc* **144**, 10053–10063 (2022).

2. Joshipura, I. D. *et al.* An atomically smooth container: Can the native oxide promote supercooling of liquid gallium? *iScience* **26**, (2023).

3. Style, R. W. & Dufresne, E. R. Static wetting on deformable substrates, from liquids to soft solids. *Soft Matter* **8**, (2012).

4. Ravazzoli, P. D., González, A. G., Diez, J. A. & Stone, H. A. Buoyancy and capillary effects on floating liquid lenses. *Phys Rev Fluids* **5**, (2020).

5. Smith, J. D. *et al.* Droplet mobility on lubricant-impregnated surfaces. *Soft Matter* **9**, 1772–1780 (2013).

6. Cai, Z., Skabeev, A., Morozova, S. & Pham, J. T. Fluid separation and network deformation in wetting of soft and swollen surfaces. *Commun Mater* **2**, (2021).

7. Lenham, A. P. The optical constants of gallium. *Proceedings of the Physical Society* **82**, (1963).

8. Moulder, J. F., Stickle, W. F., Sobol, P. E. & Bomben, K. D. Handbook of X-ray Photoelectron Spectroscopy Edited by. *Google Scholar* (1993).

9. Chen, C. Y., Chien, C. Y., Wang, C. M., Lin, R. S. & Chen, I. C. Plasmon Tuning of Liquid Gallium Nanoparticles through Surface Anodization. *Materials* **15**, (2022).

10. Rebien, M., Henrion, W., Hong, M., Mannaerts, J. P. & Fleischer, M. Optical properties of gallium oxide thin films. *Appl Phys Lett* **81**, 250–252 (2002).

11. Kuzmenko, A. B. Kramers-Kronig constrained variational analysis of optical spectra. *Review of Scientific Instruments* **76**, 1–9 (2005).

12. Miller, B. H., Liu, H. & Kolle, M. Scalable optical manufacture of dynamic structural colour in stretchable materials. *Nat Mater* **21**, 1014–1018 (2022).

13. Ordinario, D. D. *et al.* Stretchable Structural Color Filters Based on a Metal–Insulator–Metal Structure. *Adv Opt Mater* **6**, (2018).

14. Lee, G. H. *et al.* Chameleon-Inspired Mechanochromic Photonic Films Composed of Non-Close-Packed Colloidal Arrays. *ACS Nano* **11**, (2017).

15. Wei, J. *et al.* Full-color persistent room temperature phosphorescent elastomers with robust optical properties. *Nat Commun* **14**, (2023).

16. Raisch, M., Maftuhin, W., Walter, M. & Sommer, M. A mechanochromic donor-acceptor torsional spring. *Nat Commun* **12**, (2021).

17. Burel, C. A. S. *et al.* Plasmonic-Based Mechanochromic Microcapsules as Strain Sensors. *Small* **13**, (2017).

18. Geng, Y., Kizhakidathazhath, R. & Lagerwall, J. P. F. Robust cholesteric liquid crystal elastomer fibres for mechanochromic textiles. *Nat Mater* **21**, (2022).

19. McCarley, J. E., Green, C. E. & Horowitz, K. H. Digital System for Converting Spectrophotometric Data to CIE Coordinates, Dominant Wavelength, and Excitation Purity. *J Opt Soc Am* **55**, (1965).

20. Kim, S.-U. *et al.* Generation of intensity-tunable structural color from helical photonic crystals for full color reflective-type display. *Opt Express* **26**, (2018).

21. Sai, T., Froufe-Pérez, L. S., Scheffold, F., Wilts, B. D. & Dufresne, E. R. Efficient structural color from pigment-loaded nanostructures. (2023).

22. Qin, G. *et al.* Recombinant reflectin-based optical materials. *Journal of Polymer Science, Part B: Polymer Physics* vol. 51 Preprint at https://doi.org/10.1002/polb.23204 (2013).

23. Li, Q. *et al.* Sub-micron silk fibroin film with high humidity sensibility through color changing. *RSC Adv* **7**, (2017).

24. Hu, X., Zhang, X., Chen, X. & Luo, M. Solution route to large area all-TiO2 one-dimensional photonic crystals with high reflectivity and different structural colors. *Nanotechnology* **31**, (2020).

25. Daqiqeh Rezaei, S. *et al.* Tunable, Cost-Effective, and Scalable Structural Colors for Sensing and Consumer Products. *Adv Opt Mater* **7**, (2019).

26. Sol, J. A. H. P., Smits, L. G., Schenning, A. P. H. J. & Debije, M. G. Direct Ink Writing of 4D Structural Colors. *Adv Funct Mater* **32**, (2022).

27. Del Pozo, M. *et al.* Direct Laser Writing of Four-Dimensional Structural Color Microactuators Using a Photonic Photoresist. *ACS Nano* **14**, (2020).

28. Boyle, B. M., French, T. A., Pearson, R. M., McCarthy, B. G. & Miyake, G. M. Structural Color for Additive Manufacturing: 3D-Printed Photonic Crystals from Block Copolymers. *ACS Nano* **11**, (2017).

29. Liu, H. *et al.* High-Order Photonic Cavity Modes Enabled 3D Structural Colors. *ACS Nano* (2022) doi:10.1021/acsnano.2c01999.

30. Siegwardt, L. & Gallei, M. Complex 3D-Printed Mechanochromic Materials with Iridescent Structural Colors Based on Core–Shell Particles. *Adv Funct Mater* **33**, (2023).

31. Liu, Y. *et al.* Structural color three-dimensional printing by shrinking photonic crystals. *Nat Commun* **10**, (2019).

32. Fang, Y., Fei, W., Shen, X., Guo, J. & Wang, C. Magneto-sensitive photonic crystal ink for quick printing of smart devices with structural colors. *Mater Horiz* **8**, (2021).

33. Demirörs, A. F. *et al.* Three-dimensional printing of photonic colloidal glasses into objects with isotropic structural color. *Nat Commun* **13**, (2022).

34. Patel, B. B. *et al.* Tunable structural color of bottlebrush block copolymers through direct-write 3D printing from solution. *Sci Adv* **6**, (2020).

35. Tan, A. T. L. *et al.* In-Plane Direct-Write Assembly of Iridescent Colloidal Crystals. *Small* **16**, (2020).

36. Tan, A. T. L., Beroz, J., Kolle, M. & Hart, A. J. Direct-Write Freeform Colloidal Assembly. *Advanced Materials* **30**, (2018).

37. Park, C., Koh, K. & Jeong, U. Structural Color Painting by Rubbing Particle Powder. *Sci Rep* **5**, (2015).

38. Zhu, X., Shi, L., Liu, X., Zi, J. & Wang, Z. A mechanically tunable plasmonic structure composed of a monolayer array of metal-capped colloidal spheres on an elastomeric substrate. *Nano Res* **3**, (2010).

39. Chiang, Y. L. *et al.* Mechanically tunable surface plasmon resonance based on gold nanoparticles and elastic membrane polydimethylsiloxane composite. *Appl Phys Lett* **96**, (2010).

40. Millyard, M. G. *et al.* Stretch-induced plasmonic anisotropy of self-assembled gold nanoparticle mats. *Appl Phys Lett* **100**, (2012).

41. Cataldi, U. *et al.* Growing gold nanoparticles on a flexible substrate to enable simple mechanical control of their plasmonic coupling. *J Mater Chem C Mater* **2**, (2014).

42. Vatankhah-Varnosfaderani, M. *et al.* Chameleon-like elastomers with molecularly encoded strain-adaptive stiffening and coloration. *Science (1979)* **359**, (2018).

43. Hu, Y., Yang, D., Ma, D. & Huang, S. Extremely sensitive mechanochromic photonic crystals with broad tuning range of photonic bandgap and fast responsive speed for high-resolution multicolor display applications. *Chemical Engineering Journal* **429**, (2022).

44. Horák, M., Čalkovský, V., Mach, J., Křápek, V. & Šikola, T. Plasmonic Properties of Individual Gallium Nanoparticles. *Journal of Physical Chemistry Letters* **14**, (2023).

45. Catalán-Gómez, S., Redondo-Cubero, A., Palomares, F. J., Nucciarelli, F. & Pau, J. L. Tunable plasmonic resonance of gallium nanoparticles by thermal oxidation at low temperatures. *Nanotechnology* **28**, (2017).

46. Catalán-Gómez, S. *et al.* Plasmonic coupling in closed-packed ordered gallium nanoparticles. *Sci Rep* **10**, (2020).

47. Gutiérrez, Y. *et al.* Polymorphic gallium for active resonance tuning in photonic nanostructures: From bulk gallium to two-dimensional (2D) gallenene. *Nanophotonics* vol. 9 Preprint at https://doi.org/10.1515/nanoph-2020-0314 (2020).

48. Knight, M. W. *et al.* Gallium plasmonics: Deep subwavelength spectroscopic imaging of single and interacting gallium nanoparticles. *ACS Nano* **9**, 2049–2060 (2015).

49. Limantoro, C. *et al.* Synthesis of Antimicrobial Gallium Nanoparticles Using the Hot Injection Method. *ACS Materials Au* (2022) doi:10.1021/acsmaterialsau.2c00078.

50. Gao, X., Fan, X. & Zhang, J. Tunable plasmonic gallium nano liquid metal from facile and controllable synthesis. *Mater Horiz* **8**, (2021).

51. Yarema, M. *et al.* Monodisperse colloidal gallium nanoparticles: Synthesis, low temperature crystallization, surface plasmon resonance and Li-ion storage. *J Am Chem Soc* **136**, (2014).

52. Reineck, P. *et al.* UV plasmonic properties of colloidal liquid-metal eutectic gallium-indium alloy nanoparticles. *Sci Rep* **9**, 1–7 (2019).
